# Molecular Determinants Underlying Binding Specificities of the ABL Kinase Inhibitors: Combining Alanine Scanning of Binding Hot Spots with Network Analysis of Residue Interactions and Coevolution

**DOI:** 10.1371/journal.pone.0130203

**Published:** 2015-06-15

**Authors:** Amanda Tse, Gennady M. Verkhivker

**Affiliations:** 1 Graduate Program in Computational and Data Sciences, Schmid College of Science and Technology, Chapman University, Orange, California, United States of America; 2 Chapman University School of Pharmacy, Irvine, California, United States of America; University Paris Diderot-Paris 7, FRANCE

## Abstract

Quantifying binding specificity and drug resistance of protein kinase inhibitors is of fundamental importance and remains highly challenging due to complex interplay of structural and thermodynamic factors. In this work, molecular simulations and computational alanine scanning are combined with the network-based approaches to characterize molecular determinants underlying binding specificities of the ABL kinase inhibitors. The proposed theoretical framework unveiled a relationship between ligand binding and inhibitor-mediated changes in the residue interaction networks. By using topological parameters, we have described the organization of the residue interaction networks and networks of coevolving residues in the ABL kinase structures. This analysis has shown that functionally critical regulatory residues can simultaneously embody strong coevolutionary signal and high network centrality with a propensity to be energetic hot spots for drug binding. We have found that selective (Nilotinib) and promiscuous (Bosutinib, Dasatinib) kinase inhibitors can use their energetic hot spots to differentially modulate stability of the residue interaction networks, thus inhibiting or promoting conformational equilibrium between inactive and active states. According to our results, Nilotinib binding may induce a significant network-bridging effect and enhance centrality of the hot spot residues that stabilize structural environment favored by the specific kinase form. In contrast, Bosutinib and Dasatinib can incur modest changes in the residue interaction network in which ligand binding is primarily coupled only with the identity of the gate-keeper residue. These factors may promote structural adaptability of the active kinase states in binding with these promiscuous inhibitors. Our results have related ligand-induced changes in the residue interaction networks with drug resistance effects, showing that network robustness may be compromised by targeted mutations of key mediating residues. This study has outlined mechanisms by which inhibitor binding could modulate resilience and efficiency of allosteric interactions in the kinase structures, while preserving structural topology required for catalytic activity and regulation.

## Introduction

Protein kinases act as dynamic molecular switches in cellular signaling and their functional activity is essential for the integrity and viability of signaling pathways involved in cell cycle control, organism development, and stress response [1–12]. The human protein kinases represent one of the largest protein families that orchestrate functional processes in cellular networks and comprise an important class of therapeutic targets, owing to the presence of a highly conserved ATP binding pocket that can be exploited by small molecule inhibitors [13–17]. Due to evolutionary conservation of the ATP binding site and structural similarity of the protein kinase folds, most ATP-competitive kinase inhibitors can promiscuously inhibit multiple kinases. Understanding of the molecular determinants underlying binding specificities of the kinase inhibitors and the development of selective and multi-target kinase drugs with a desirable activity profile are of fundamental and practical importance and remain to be highly challenging. The continuously growing body of structural and functional studies has revealed that protein kinase activity and binding can be regulated via a dynamic equilibrium between distinct functional states: active, inactive, and Src-like inactive conformations [18–24]. A diverse repertoire of crystallographic conformations has also indicated that molecular switching mechanism of protein kinases may not necessarily imply an on–off binary operation (from inactive to active), but could rather represent a continuous dynamic process in which kinases may adopt a wide spectrum of inactive-like and active-like conformations exhibiting a range of activity levels. Conformational transitions between kinase states are orchestrated by three conserved structural motifs in the catalytic domain: the αC-helix, the DFG-Asp motif (DFG-Asp in, active; DFG-Asp out, inactive), and the activation loop (A-loop open, active; A-loop closed, inactive). The conserved His-Arg-Asp (HRD) motif in the catalytic loop and the DFG motif are coupled with the αC-helix to form conserved intramolecular networks termed regulatory spine (R-spine) and catalytic spine (C-spine) whose assembly and stabilization are intimately linked with the conformational transformations and kinase activation [25,26].

The equilibrium between functional kinase states can be modulated and often redistributed by activation mutations, posttranslational modifications, protein interactions, and binding of small molecule inhibitors. On the basis of the molecular mechanism of action, one can distinguish three major classes of kinase inhibitors (types 1, 2 and 3) [14–17]. Type 1 inhibitors target the catalytically competent active (DFG-in) conformation of the kinase domain, while type 2 inhibitors recognize the inactive DFG-out kinase conformation. It has been long assumed that type 1 inhibitors are less specific than type 2 inhibitors because the active conformation is very similar in most kinases. Type 3 allosteric inhibitors do not compete with ATP and tend to be more selective than types 1 and 2 inhibitors by binding to the regulatory sites outside of the ATP binding site. The discovery of Imatinib, a highly selective type 2 inhibitor targeting ABL, KIT, and PDGFR kinases by recognizing a specific inactive conformation, launched a new era in the development of the tyrosine kinase inhibitors and galvanized a stream of structure-functional studies of ABL kinase regulation [27–30]. Nilotinib is a second generation type 2 inhibitor and a 20-fold more potent ABL inhibitor than Imatinib, but both Imatinib and Nilotinib share high specificity towards ABL kinase and bind to a specific inactive conformation (DFG-out/αC-helix-in, A-loop closed) [31,32]. Ponatinib is a third-generation type 2 inhibitor that circumvents drug resistance against the T315I mutation and is more potent and less selective inhibitor than both Imatinib and Nilotinib [33, 34]. Despite structural similarities of the ABL kinase complexes with the type 2 inhibitors, Ponatinib inhibits activity of multiple kinases including members of the VEGFR, PDGFR, FGFR, EPH receptor and SRC families of kinases, KIT, RET, TIE2, and FLT3. Dasatinib is a type 1 inhibitor that targets the catalytically competent active ABL conformation [23] and has a broad spectrum of activity against the SRC, CSK, TEC, and EPH families of tyrosine kinases. Large-scale profiling studies have revealed a significant promiscuity of Dasatinib that can target over 30 different tyrosine and Ser/Thr kinases [35–39]. Crystal structures of Dasatinib complexes with ABL [23], SRC [40], LYN [41], EPHA4 [42], BMX [43], and BTK kinases [44] have supported the notion that functional promiscuity of this inhibitor may be associated with conformational tolerance to multiple kinase forms. Structural plasticity of protein kinase conformations in complexes with Dasatinib ranged from fully active DFG-in states (in ABL, SRC, LYN, EPHA4, and BTK kinases) to an inactive DFG-out conformation of BMX [43], and an intermediate DFG-out form of BTK kinase [44]. NMR studies of Dasatinib binding with ABL kinase [45] have reassured structural preferences of the inhibitor to bind an active DFG-in form, but this conformational state may not be mandatory for a productive inhibitor binding. Recent studies have revealed that Dasatinib binding is phosphorylation state-independent and can be compatible with the DFG-out inactive ABL conformation, as the thermodynamic preferences for association with the active kinase state appeared to be only marginal [46]. Bosutinib is another type 1 inhibitor with a broad spectrum of activity against SRC and Ser/Thr kinases [47]. The binding modes of Bosutinib and Dasatinib in complexes with ABL kinase are similar, but the kinase domain can adopt a specific conformation in response to Bosutinib binding with an inactive DFG-out conformation and an open, active conformation of the A-loop [48]. Structural and spectroscopic analysis showed that binding affinities of Bosutinib with the phosphorylated and unphosphorylated ABL kinase were virtually indistinguishable, suggesting that this promiscuous inhibitor is also conformationally permissive and can bind to both DFG-in and DFG-out kinase conformations [48]. Recently, the crystal structure of Bosutinib complex with SRC kinase revealed an active DFG-in protein conformation and a similar inhibitor binding mode to that observed in the ABL complex [49].

Comprehensive analyses of kinase inhibitor selectivity probed the interactions of many known kinase inhibitors against panels of kinase targets representing more than 80% of the human protein kinome [50–52]. A complex interaction pattern has unveiled a wide spectrum of promiscuity in which off-target interactions could often occur with a different kinase subfamily than the subfamily of the intended kinase target. In the kinase inhibitors developed against tyrosine kinases a spectrum of binding promiscuity was less broad, with ~24% of off-targets representing Ser/Thr kinases [52]. A mass spectrometry-based proteomic approach identified the direct targets and downstream signaling effect of Imatinib, Nilotinib, Dasatinib, and Bosutinib in epidermoid carcinoma cells [53]. This study confirmed similarities in the binding promiscuity of Dasatinib and Bosutinib that displayed a broad target spectrum (19 and 18 tyrosine kinases, respectively) including members of the SRC kinase family (e.g., YES, LCK, LYN, and FRK) and members of the ephrin receptor family (EPHA2, EPHB2, EPHB3, and EPHB4 kinases). A systematic analysis of type 2 inhibitors has recently shown that high selectivity and strong sensitivity towards the unphosphorylated form of ABL kinase is not a universal characteristic of type 2 inhibitors that bind to the DFG-out conformation [54]. A survey of type 2 kinase inhibitors has concluded that many kinases may be inherently predisposed for binding with this class of small molecules since the DFG-out conformation may be thermodynamically native state for some kinases [55]. Although type 2 inhibitors are generally more selective than type 1 inhibitors, selectivity may be also achieved with a type 1 binding mode and is not guaranteed with a type 2 binding mode.

A number of biochemical and clinical studies have investigated the activity, frequency, clinical relevance, and the conformational changes induced by drug resistant mutations in the ABL kinase [56–65]. These studies have determined that 15 major point mutations (T315I, Y253F/H, E255K/V, M351T, G250E, F359C/V, H396R/P, M244V, E355G, F317L, M237I, Q252H/R, D276G, L248V, and F486S) that account for 85% of recorded substitutions. In patients who have developed Nilotinib resistance, frequently detected mutations include P-loop mutations (Q252H, Y253H/F, E255K/V), V299L, T315I/A, F317L, and F359C/V [56–59]. Dasatinib is active against most of the ABL mutations known to confer resistance to Imatinib, except for the T315I/A, V299L, F317L/V/I/C, F359V mutations [60, 61]. Ponatinib retains potency against many Imatinib-resistant mutants, including T315I variant of the gate-keeper residue [62–66]. Although no single point mutation has shown high resistance against Ponatinib, a few clinically reported double mutants, such as G250E/T315I, E255K/T315I, and E255V/T315I have shown an increased level of drug resistance. Clinical studies have also validated and confirmed a range of mutations for which changes in the binding affinity and inhibition constants could strongly correlate with the degree of resistance to Imatinib, Nilotinib, and Dasatinib [67–69].

Computational studies have investigated molecular mechanisms of protein kinases and structural effects of drug resistance mutations [70–81]. Molecular dynamics (MD) simulations and free energy calculations have shown that conformational selection and the stability difference between the inactive DFG-out and active DFG-in conformations may be the primary determinant underlying Imatinib specificity [76,77]. Integration of modeling approaches and isothermal titration calorimetry experiments have quantified conformational dynamics and stability of the ABL conformations that could define the mechanism of Imatinib selectivity [78]. The absolute binding free energies of Imatinib binding with ABL and SRC kinases were computed in a large-scale simulation study [79] and scrutinized at atomic details the proposed mechanisms of Imatinib selectivity, suggesting that conformational selection mechanism and subtle differences in the interactions with the binding site residues may be collectively responsible for binding specificity. MD simulations have also explored thermodynamic preferences of Dasatinib for binding to the active ABL state, suggesting that favorable inhibitor interactions with the assembled R-spine may contribute to the stability of the active complex [82]. Mechanisms of Dasatinib and Ponatinib resistance to clinically relevant ABL polymutants were recently investigated using a combination of simulations and biochemical assays [83], showing that Ponatinib can maintain binding affinity not only for most single ABL mutants but also for some of the polymutants detected in the studied patient pool. Recently published MD simulations of Ponatinib binding with a large panel of Imatinib-resistant ABL mutants have reached similar conclusions [84]. The thermodynamic factors underlying kinase inhibitor specificities and drug resistant profiles can be evaluated by combining biophysical simulations and free energy simulations with alanine scanning mutagenesis. Computational alanine scanning approach pioneered by late Peter Kollman and colleagues [85,86] can estimate the energetic contribution of each residue to the total binding energy through systematic alanine modifications by employing the molecular mechanics (MM) force field [87] combined with the generalized Born and solvent accessible surface area (GB/SA) solvation model [88,89]. This protocol assumes that mutations would not cause significant conformational changes and global rearrangements in the residue interactions.

Complex changes in the kinase-inhibitor interactions obtained from X-ray crystallography, NMR studies, and large-scale computer simulations often reflect not only local variations at the binding interfaces, but also subtle yet important changes in the residue interaction networks. A graph-based representation of protein structures yields a convenient description of residue interaction networks [90–93], providing a robust framework for understanding allosteric communications in protein systems. Structure-based network models of protein structures often employ various measures of node centrality (degree, closeness, and betweenness) to characterize local and global connectivity in the residue interaction networks. The network centrality parameters have been robust predictors of functional residues that regulate protein-protein interactions [94, 95], ligand binding [96, 97], enzyme catalysis [98] and allosteric signaling [99]. Functional sites that mediate stability of the residue interaction networks are also involved in the networks of coevolving protein residues. Statistical coupling analysis (SCA), mutual information (MI) model and related covariance-based approaches have employed sequence-based analysis of residue coevolution in homologous families to show that functional residues are connected via strong coevolutionary relationships [100–107]. Coevolution of protein residues can reflect a coordinated involvement of these sites in mediating residue-residue contacts [108] thus promoting protein folding [109], facilitating protein recognition and allosteric signaling in multi-protein complexes [110], controlling an enzymatic activity and progression of disease-associated phenotypes and drug resistant variants [111,112]. Coevolving residues tend to be spatially coupled and correspond to functionally important sites exhibiting correlated and compensatory mutations in homologous proteins [113]. These residues could also form networks with connections corresponding to coevolutionary interaction strengths between nodes, where the underlying small-world topology of such networks is similar to the structure-based residue interaction networks [114–116]. Furthermore, coevolving residues can assemble into structurally stable and quasi-independent modules of physically interacting residues termed ‘protein sectors’ [103,104]. Integrated analysis of residue coevolution networks and residue interaction networks can capture the emergence of independent modules of functional residues that cooperatively mediate structural stability and conformational transitions required for diverse functions in the dynamic protein environment [117–123].

In the current work, we present a theoretical framework for rationalizing binding specificities of the ABL kinase inhibitors by dissecting a relationship between ligand binding and residue interaction networks in the kinase structures. The primary hypothesis addressed in this study is that kinase inhibitors could effectively intervene into organization of the residue interaction networks through binding hot spots that also function as global mediating sites in protein kinase structures. We characterize the residue interaction networks and networks of coevolving residues in the kinase structures, revealing a selected group of high centrality residues that may be involved in kinase activity, regulation and binding. MD simulations and MM-GBSA alanine scanning of binding interactions are combined with the structure-based network analysis to probe ligand-induced changes in the residue interaction networks. This analysis demonstrates that binding specificity and drug resistance effects may be adequately described by the unique networking signatures of the kinase complexes. We show that binding sensitivities of the kinase inhibitors may be exposed through energetic coupling with the hot spot residues that regulate conformational equilibrium and can modulate stability of the kinase states. Our study offers a systems-based perspective on drug design by investigating how selective and promiscuous inhibitor binding can be interrelated with the efficiency and robustness of the residue interaction networks in the kinase structures.

## Results and Discussion

### Conformational Dynamics of the Protein Kinase Complexes

**Fig 1:**
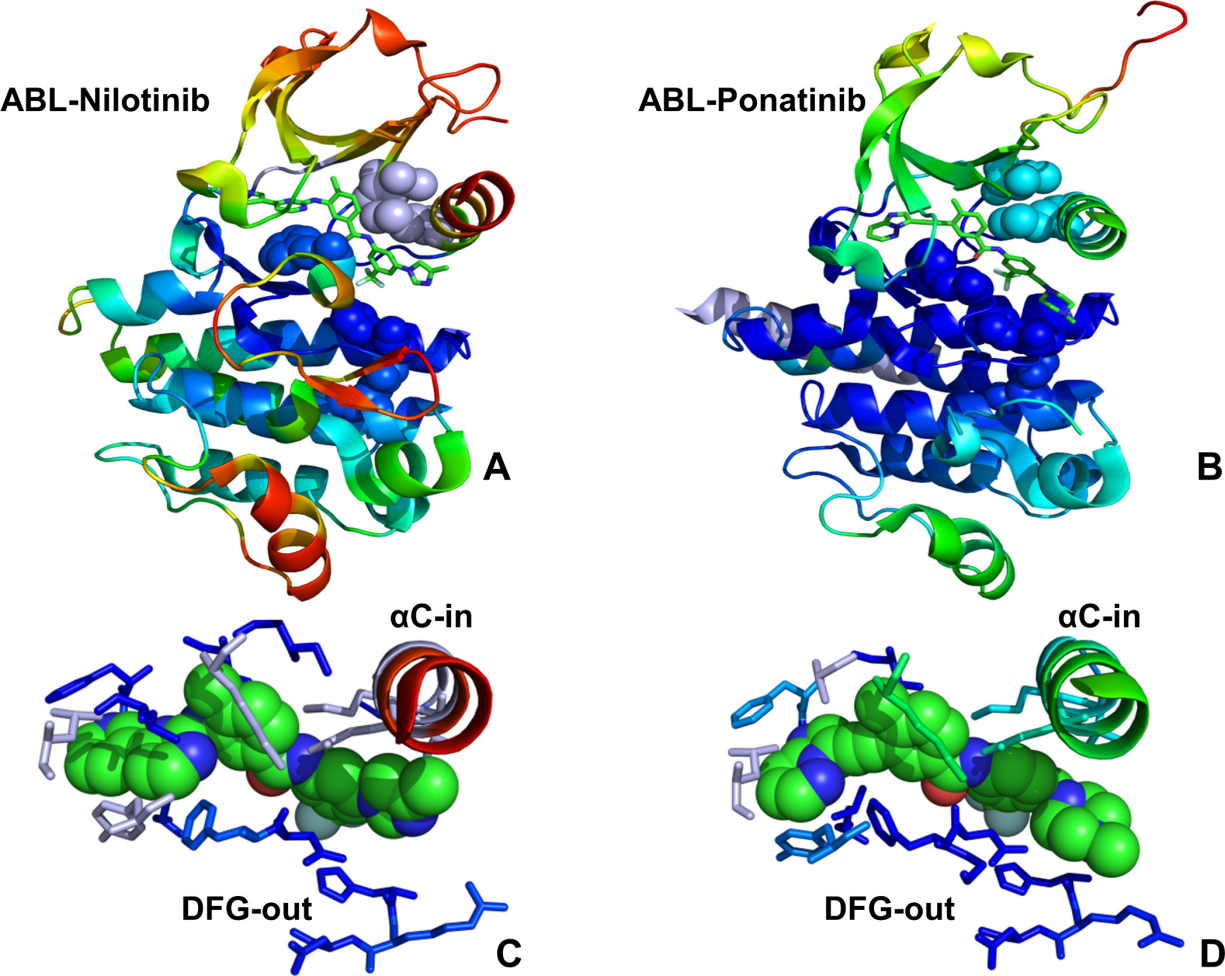
Conformational Dynamics of the ABL Complexes with Type 2 Inhibitors. Conformational dynamics profiles are shown for the crystal structures of the ABL complexes with type 2 inhibitors Nilotinib (pdb id 3CS9, panel A) and Ponatinib (pdb id 3OXZ, panel B). Conformational dynamics profiles were computed by projecting MD trajectories onto the space of three lowest frequency modes. The color gradient from blue to red indicates the decreasing structural rigidity (or increasing conformational mobility) of the protein residues and refers to an average value over the backbone atoms in each residue. The R-spine residues are annotated in spheres and colored according to their degree of structural stability. A partially disjointed architecture of the R-spine is characteristic of the inactive ABL conformation in the crystal structures. The inhibitors are shown in sticks and atom-based color-coded. The inhibitor binding modes and binding site residues are shown for Nilotinib (C) and Ponatinib (D). The highlighted αC-helix position (αC-in) and DFG-out conformation are characteristic of the inactive ABL conformation in the complexes with type 2 inhibitors.

**Fig 2:**
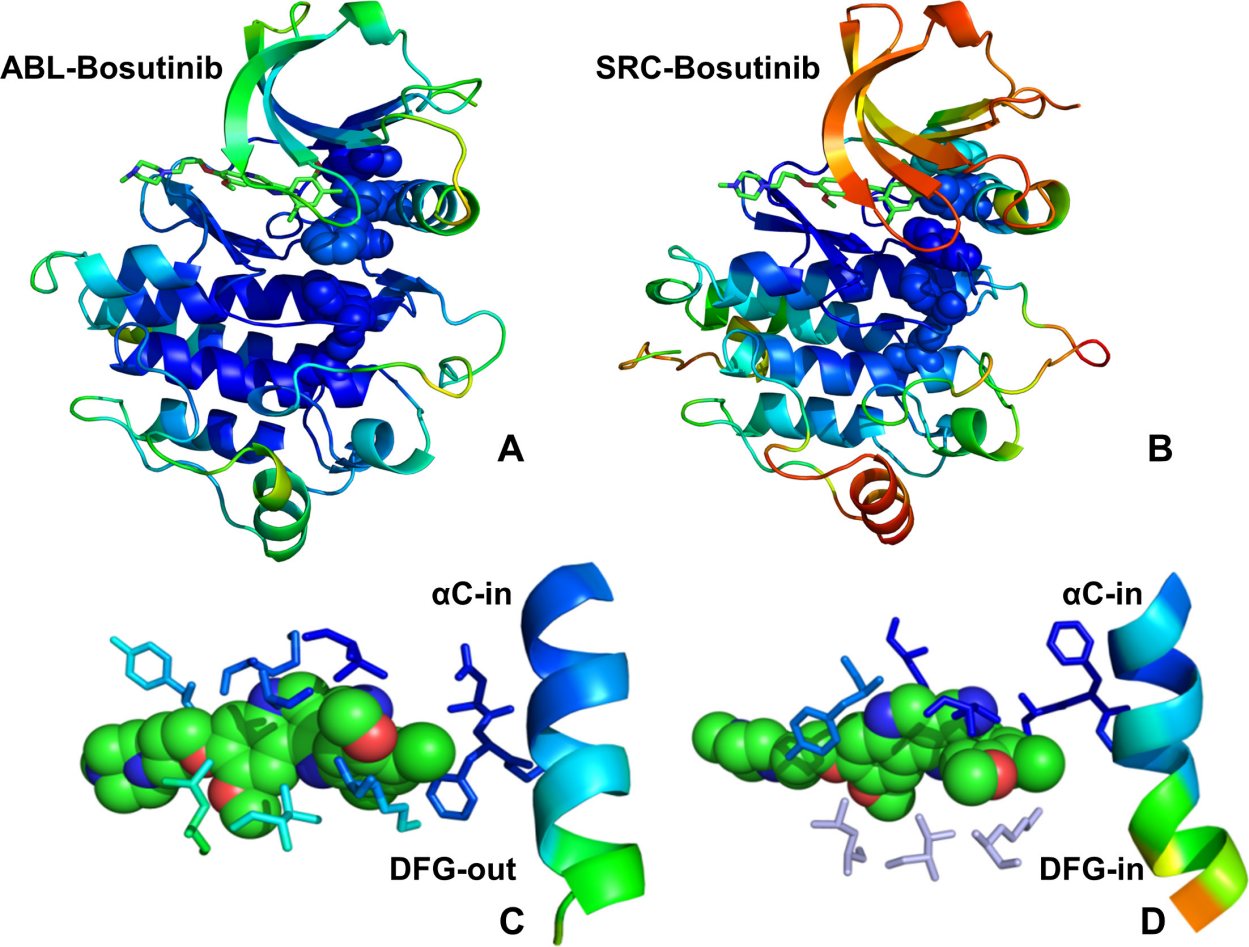
Conformational Dynamics of the ABL and SRC Complexes with Type 1 Inhibitors. Conformational dynamics profiles are shown for the crystal structures of Bosutinib complexes with ABL (pdb id 3UE4, panel A) and SRC kinases (pdb id 4MXO, panel B). Conformational dynamics profiles were computed by projecting MD trajectories onto the space of three lowest frequency modes. The color gradient from blue to red indicates the decreasing structural rigidity (or increasing conformational mobility) of the protein residues and refers to an average value over the backbone atoms in each residue. The R-spine residues are annotated in spheres and colored according to their degree of structural stability. A fully assembled architecture of the R-spine is characteristic of the active kinase conformations in the crystal structures. The inhibitors are shown in sticks and atom-based color-coded. A close up of the inhibitor binding mode and interacting residues is shown for ABL kinase (C) and SRC kinase (D). The different orientation of the regulatory kinase motifs in otherwise similar complexes with Bosutinib is highlighted: DFG-out/αC-helix-in conformation in ABL kinase (C) and DFG-in/αC-helix-in conformation in SRC kinase (D).

Using MD simulations, we first characterized conformational dynamics of the ABL kinase complexes with the type 2 inhibitors (Nilotinib, Ponatinib) and type 1 inhibitor Bosutinib. Although Nilotinib and Ponatinib share a similar binding mode targeting a specific inactive ABL conformation (DFG-out/αC-helix-in, A-loop closed) (Fig 1), these drugs have a drastically different kinase specificity profiles and binding sensitivities towards Imatinib-resistant ABL mutants. In the ABL kinase complex with the type 1 inhibitor Bosutinib, the kinase domain revealed an uncharacteristic inactive position of the DFG motif (DFG-out/αC-helix-in) but retained an open active conformation of the A-loop that is similar to that observed in the active kinases (Fig 2A). However, in the Src kinase complex with Bosutinib, the kinase domain adopts an active DFG-in/αC-helix-in conformation and an open A-loop conformation that are structurally similar to Dasatinib complexes with ABL and SRC kinases (Fig 2B). In the course of simulations, we tracked conformational variations of the αC-helix and the A-loop regions, particularly focusing on structural stability and ligand-induced changes in the R-spine and C-spine intramolecular networks. The R-spine in ABL consists of M290 from the C-terminal end of the αC-helix, L301 from the β4-strand, F382 of the DFG motif in the beginning of the A-loop, H361 of the HRD motif in the catalytic loop, and D421 of the αF-helix (Figs 1 and 2). The C-spine is comprised of hydrophobic residues (V256, A269, L323, C369, L370, V317, L438, and I432) that connect the kinase lobes anchoring catalytically important sites to the hydrophobic C-terminus of the αF-helix (L438, I432).

**Fig 3:**
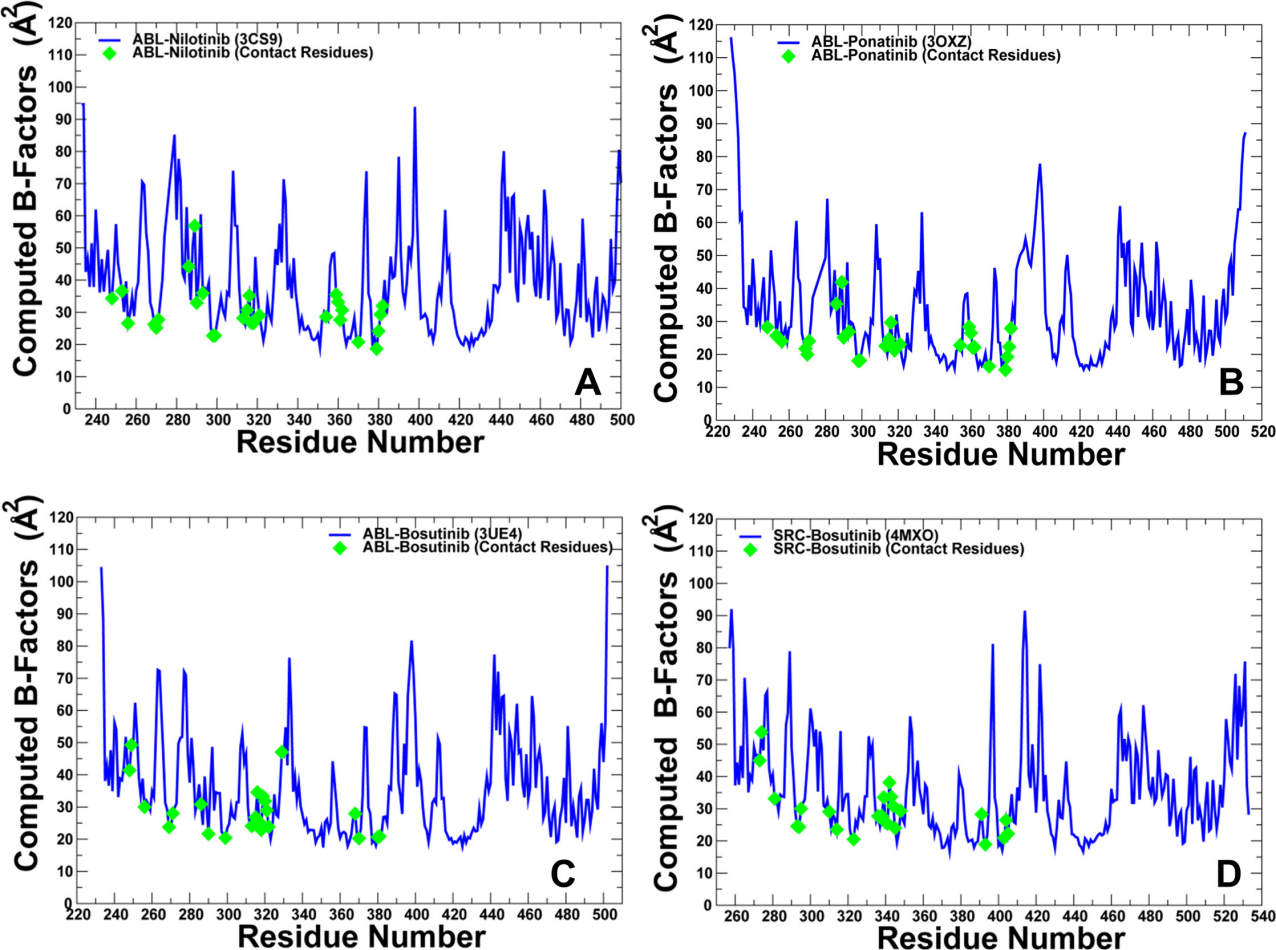
MD Simulations of the Kinase-Inhibitor Complexes: Equilibrium Fluctuations of Protein Residues. The computed B-factors obtained from MD simulations of the ABL complexes with Nilotinib (A), Ponatinib (B), Bosutinib (C) and SRC complex with Bosutinib (D). The fluctuations of the inhibitor-interacting residues are highlighted (green diamonds) indicating stability of the binding site residues in simulations.

To characterize functional motions of the kinase complexes we determined conformational mobility profiles using principal component analysis (PCA) [124]. In the current study, we performed PCA of protein conformational dynamics based on the backbone heavy atoms (N, Cα, Cβ, C, O) and the Cα atoms only, both producing very similar profiles. The conformational mobility profiles were computed along the three low frequency modes revealing conservation of the kinase dynamics as well as structural stability of the catalytic core and the R-spine residues. Although the R-spine was partially disassembled in the ABL complexes with Nilotinib (Fig 1A), Ponatinib (Fig 1B) and Bosutinib (Fig 2A), such structural arrangement of the intramolecular network remained stable during simulations of the kinase complexes. This indicated that both types of kinase inhibitors may be compatible with the DFG-out conformation and a disjointed structure of the R-spine. Furthermore, similar functional dynamics of Bosutinib complexes with ABL (DFG-out conformation) and SRC kinases (DFG-in conformation) supported the notion that this type 1 inhibitor may be dynamically adaptable and relatively insensitive to the R-spine architecture (Fig 2). The conformational fluctuations based on the computed B-factors of the backbone residues were generally similar in all complexes (Fig 3). While the P-loop (residues 248–255) displayed small fluctuations in the ABL complexes with Nilotinib and Ponatinib (Fig 3A and 3B), the greater conformational flexibility of this motif was observed in the Bosutinib complexes (Fig 3C and 3D). A partially increased mobility of the P-loop in the ABL-Bosutinib complex may reflect coupling between the P-loop (Y253) and DFG motif (F382) that maintains the DFG-out conformation (Fig 3C). In the SRC-Bosutinib complex, the conformational flexibility of the P-loop (residues 273–279) further increased, while the αC-helix (residues 303–317), and A-loop (residues 403–430) displayed similar mobility to that seen in the ABL-Bosutinib complex (Fig 3D).

We also simulated conformational dynamics of Dasatinib binding using crystal structures of Dasatinib complexes with ABL [23], SRC [40], LYN [41], EPHA4 [42], BMX [43], and BTK [44], and P38 kinases. The projection of MD trajectories onto the essential space of low frequency modes revealed characteristic similarities in the distribution of structurally rigid and conformationally flexible regions of the kinase core (S1 Fig). The computed fluctuations of the backbone residues indicated that the binding site residues interacting with Dasatinib were stable (S2 Fig). Conformational dynamics of Dasatinib binding with kinase targets was consistent with the HX-MS experiments of the inhibitor binding with ABL kinase that revealed only small dynamic changes localized near the ATP binding site, while other regions in the kinase domain remain mostly unaffected [125]. Structural alignment of the ensemble-average positions for the binding site residues revealed their considerable structural conservation, which was particularly pronounced for T315, F317, M318, and L370 (S3 Fig). Structural rigidity of these residues may be contrasted with some positional variability of the DFG motif. In Dasatinib complexes with the DFG-out conformations of BMX and BTK kinases the αC-helix moved away from the catalytically competent position and the characteristic salt bridge was broken (S4 Fig). Nonetheless, the binding mode of Dasatinib was virtually unchanged in the respective crystal structures and remained stable during simulations.

### Computational Alanine Scanning of the Binding Site Residues: Energetic Hot Spots of the Type 1 and Type 2 Kinase Inhibitors

**Fig 4:**
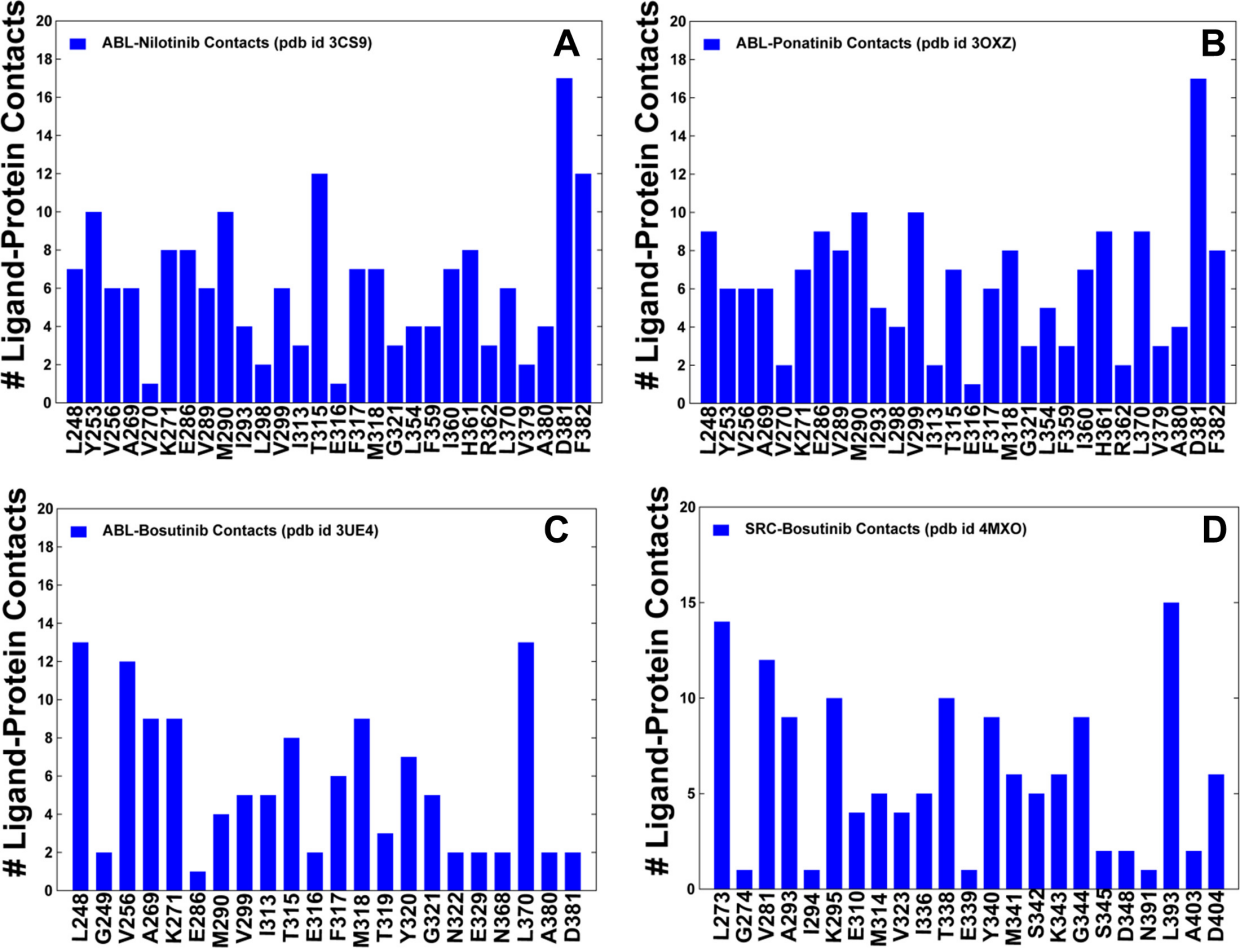
The Ensemble-Based Distributions of the Intermolecular Contacts in the ABL and SRC Complexes with Type 1 and Type 2 Inhibitors. The ensemble-based averages of the intermolecular contacts in the ABL and SRC complexes are obtained from MD trajectories. The number of the intermolecular contacts formed by the inhibitors with the binding site residues is shown (blue filled bars) for the ABL-Nilotinib complex (A), ABL-Ponatinib complex (B), ABL-Bosutinib complex (C), and SRC-Bosutinib complex (D). The intermolecular contacts were evaluated by applying the LPC program to the MD-based ensemble of structures and using a LPC-based classification scheme that includes 8 classes of atom types to define interactions: hydrophilic, hydrophobic, aromatic, acceptor, donor, neutral, neutral-donor, neutral-acceptor. The computed intermolecular contacts accounted for protein kinase residues and ligand atoms that interact through hydrogen bonds, hydrophobic contacts, aromatic-aromatic, and hydrophilic-hydrophobic interactions.

**Fig 5:**
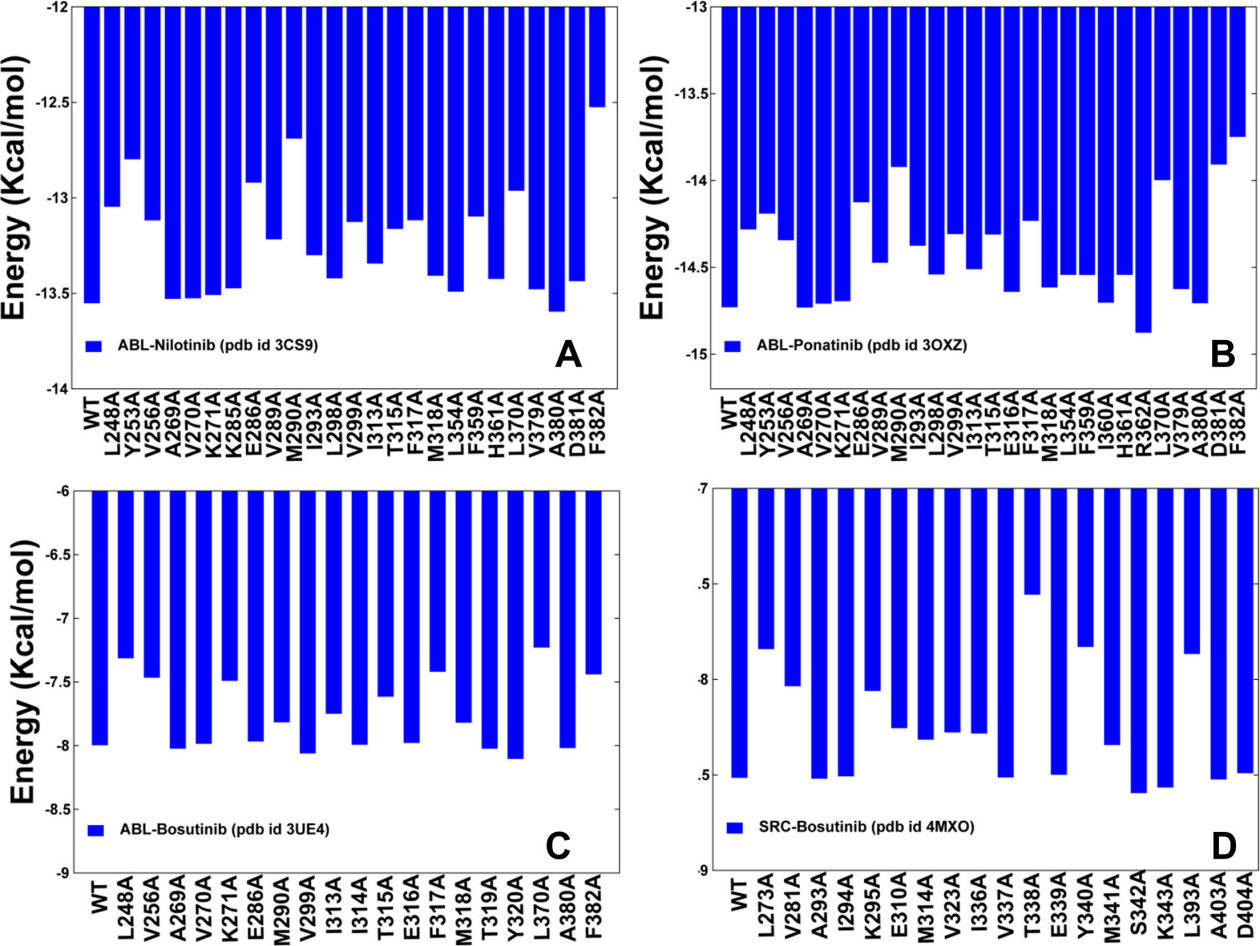
Computational Alanine Scanning of the Binding Site Residues in the ABL and SRC Complexes with Type 1 and Type 2 Inhibitors. Binding free energies and alanine scanning of the binding site residues for the ABL-Nilotinib complex (A), ABL-Ponatinib complex (B), ABL-Bosutinib complex (C), and SRC-Bosutinib complex (D). Computational alanine scanning evaluated the effect of mutations in the active site residues on binding affinity using MD trajectories of the wild type (WT) complexes and MM-GBSA calculations. The protocol involved a systematic modification of the inhibitor-interacting residues to alanine by eliminating side-chain atoms beyond C_β_, and measuring the effect of each mutation on binding affinity.

MD simulations were employed to perform binding free energy calculations followed by a systematic alanine scanning of the binding site residues in the studied complexes. The central objective of this analysis was to determine key functional residues and identify energetic hot spots that may contribute to the binding preferences of the inhibitors. We first evaluated the equilibrium distributions of the intermolecular contacts in simulated complexes using the Ligand Protein Contact (LPC) program [126]. Structural similarity of Nilotinib and Ponatinib binding modes produced nearly identical profiles of the inhibitor-kinase contacts (Fig 4A and 4B). Both Nilotinib and Ponatinib could form a number of stable intermolecular contacts with the P-loop residue Y253, catalytic residues K271, E286, the R-spine residue M290, the gate-keeper residue T315, the catalytic HRD triad (H361, R362), and the DFG motif (D381, F382). In the ABL-Nilotinib complex, the P-loop folds over the adenine pocket in the kinked conformation, and a group of hydrophobic residues (L248, Y253, F317, L370) encloses the pyridine and pyrimidine groups of the inhibitor. Binding free energies and alanine scanning confirmed that specific Nilotinib binding may be primarily associated with the favorable interactions made with Y253, M290, T315, F317, F359, L370, and F382 (Fig 5A). Structural coupling between these functional sites from the P-loop, αC-β4-loop, A-loop, HRD and DFG motifs is important to ensure thermodynamic stability of the inactive ABL conformation structure and thus may contribute to the binding specificity of Nilotinib. It is worth stressing that some of the energetic hot spots anchoring specific Nilotinib binding included sites of drug resistant mutations Y253, T315, and F317. In patients who have developed Nilotinib resistance, frequently detected mutations included Q252H, Y253H, E255K/V, V299L, T315I/A, F317L, and F359C/V [56–58]. An important feature of Ponatinib binding is a network of hydrophobic contacts formed by the trifluoromethyl substituent on the phenyl ring with the I293, L298, V299, M290, V359, V379, A380, H361, and L354 residues (Fig 4B). The energetic analysis suggested that multiple contacts formed by Ponatinib could provide a more balanced distribution of binding interactions since the individual alanine substitutions resulted in a moderate loss of binding energies (Fig 5B). Consistent with the experiments [33, 34], we determined that mutations of Ponatinib-interacting residues may produce only a moderate reduction in binding affinity. In particular, a relatively minor change in the binding energetics could be seen for T315A, which is consistent with the fact that Ponatinib is capable of escaping mutations at the critical gate-keeper position. At the same time, a few residues including E286, M290, L370, F317, D381, and F382 caused a larger change in the binding interactions, suggesting that these sites may act as the energetic anchors of Ponatinib binding to ABL kinase. These residues are the integral elements of the R-spine (M290, F382) and C-spine (L370) subnetworks. Accordingly, the energetic hot spots of these type 2 inhibitors corresponded to the key functional residues that define structural architecture of the specific ABL conformation. Nonetheless, binding sensitivities of Ponatinib may not be immediately apparent from alanine scanning and analysis of local binding interactions. Based on this evidence, we suggested that Ponatinib binding may induce global changes in the residue interaction networks.

For type 1 inhibitor Bosutinib, the total number of the intermolecular contacts with ABL was relatively moderate (Fig 4C). The favorable inhibitor interactions were primarily made with L248, C-spine residues (V256, A269, L370), and the hinge residues T315, M318. Interestingly, this type 1 inhibitor maintained only a limited number of contacts with the DFG-out motif and the A-loop during simulations. Despite a different orientation of the DFG motif (DFG-in) in the SRC complex, the distribution of the intermolecular contacts was mostly unchanged, with the most favorable interactions formed by the hinge residues (T338, Y340) and C-spine residues V281, A293, L393 (Fig 4D). Hence, binding preferences of Bosutinib may be relatively insensitive to the DFG conformation and therefore better tolerate conformational fluctuations of the kinase domain. Computational alanine scanning confirmed that Bosutinib binding may be primarily determined by contact residues L248, T315, F317, and L370 (Fig 5C and 5D). Importantly, these residues serve as energetic hot spots for both type 1 and type 2 inhibitors. These findings were also consistent with the recent experiments in which L248R and T315V showed resistance to both type 1 (Imatinib, Nilotinib, Ponatinib) and type 2 inhibitors (Bosutinib, Dasatinib) [64]. In addition, F317R mutant was moderately resistant to Imatinib and Nilotinib, but highly resistant to Dasatinib, Bosutinib and Ponatinib.

**Fig 6:**
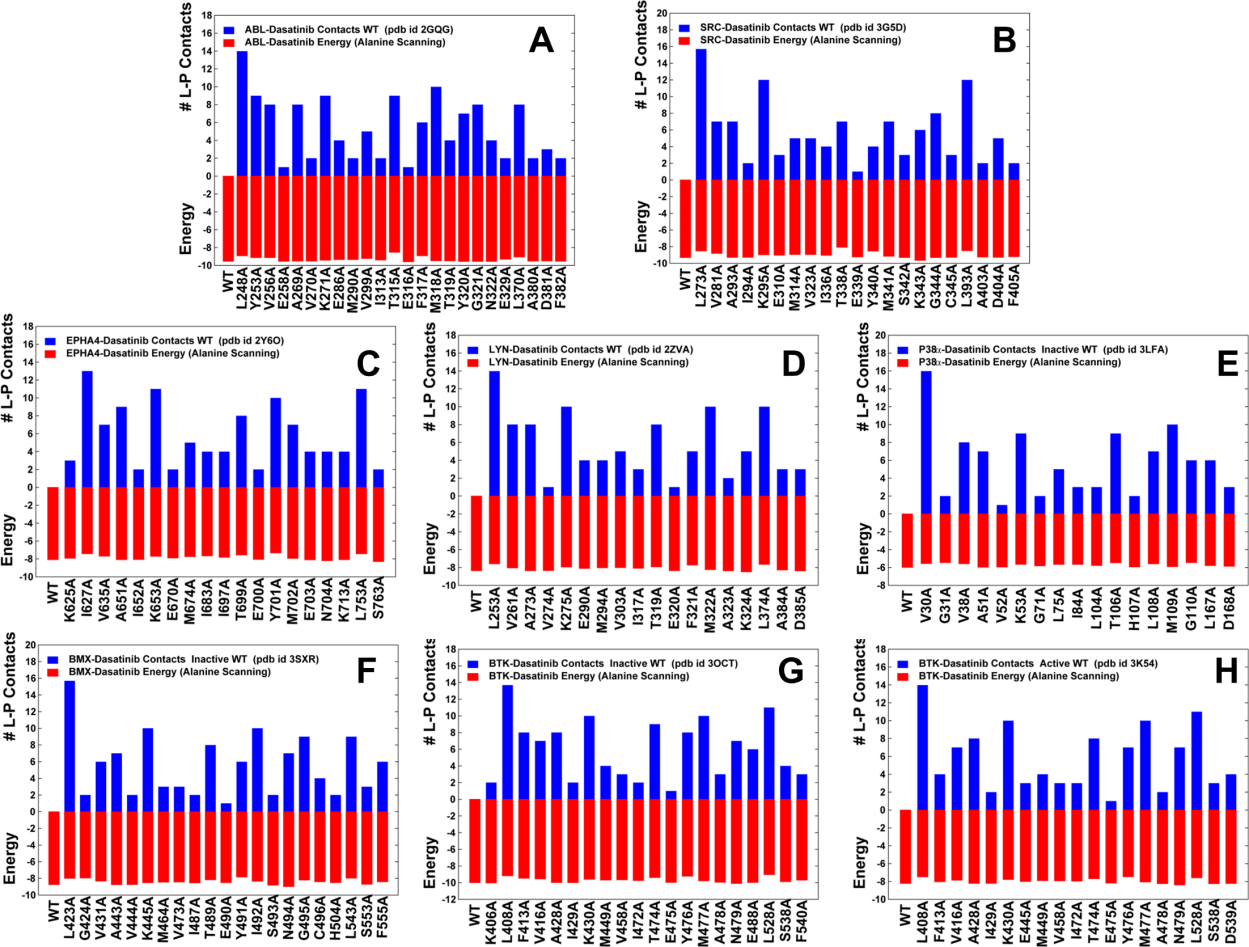
The Ensemble-Based Distributions of the Intermolecular Contacts and Alanine Scanning of the Binding Site Residues in Dasatinib-Kinase Complexes. The ensemble-based numbers of the intermolecular contacts formed by Dasatinib with the binding site residues are shown (as blue filled bars) for complexes with ABL (A), SRC (B), EPHA4 (C), LYN (D), P38 (E), BMX (F), and BTK kinases (G,H). An active DFG-in conformation is adopted Dasatinib complexes with ABL (pdb id 2GQG), SRC (pdb 3G5D), EPHA4 (pdb id 2Y6O), LYN (pdb id 2ZVA), P38 (pdb id 3LFA), and BTK kinases (pdb id 3K54). Dasatinib binds to a DFG-out conformation of the inactive BMX kinase (pdb id 3SXR). In the complex with an inactive nonphosphorylated BTK conformation (pdb id 3OCT) an intermediate DFG position is adopted which is between the fully DFG-in and DFG-out conformations. Binding free energies and alanine scanning results in Dasatinib complexes are shown on the same graphs in filled red bars.

The distribution of Dasatinib-kinase contacts was fairly consistent across kinase targets, dominated by the favorable binding interactions with the hinge residues. The primary residues involved in stable ABL interactions with Dasatinib included L248, K271 (catalytic salt bridge), T315 (gate-keeper), M318 (hinge), L370 (C-spine) (Fig 6A). In general, alanine scanning of the binding site residues across the Dasatinib complexes resulted in similarly small changes of the binding energies (Fig 6), which is markedly different from selective Nilotinib binding. The noticeable changes were only observed upon substitutions of the gate-keeper residue (T315 in ABL) that is conserved in all crystal structures and may function as a primary hot spot of Dasatinib binding. In addition, mutations of L248, K271, F317, M318, and L370 residues appeared to detrimentally affect binding energetics of Dasatinib to ABL kinase. Some of these residues (K271, M318, and L370) are important for the kinase activity, while mutations in other residues (L248, T315, and F317) may be associated with Dasatinib resistance [59–61, 64]. Importantly, alanine mutations of the corresponding residues in other kinases led to similar appreciable reductions in the binding affinities (Fig 6). We found that Dasatinib binding to kinase targets may be primarily associated with the following energetic hot spots: I627, T669, Y470, L753 (EPHA4); L253, T319, F321, L374 (LYN); L423, T489, Y491, L543 (BMX); L408, T474, Y476, L528 (BTK). In the course of simulations, Dasatinib binding pose remained stable in all kinase complexes, confirming that the inhibitor binding energetics may be relatively insensitive to variations of the αC-helix, A-loop, the DFG motif and the R-spine residues (S4 Fig). These factors may promote conformational tolerance and encourage structural adaptability of the active kinase states in formation complexes with Bosutinib and Dasatinib.

### The Residue Interaction Networks of the Kinase Structures and Binding Sensitivities of the ABL Kinase Inhibitors

Our results indicated that computational alanine scanning experiments may primarily reflect the effect of local binding interactions. However, functional role of the binding hot spots may be also linked with their global centrality in the residue interaction networks. Mutations of these residues may simultaneously alter multiple interactions, causing reorganizations in the global network and triggering shifts in the conformational equilibrium of kinase states. To probe binding sensitivities of the kinase inhibitors, we combined computational alanine scanning with the structure-based network analysis of the kinase-inhibitor complexes. We evaluated ligand-induced changes in the residue interaction networks to determine a mechanism by which selective and promiscuous inhibitors can use energetic hot spots to modulate stability of the residue interaction networks. Residue betweenness which is a global centrality measure was used as a stability probe in evaluating residue interaction networks in kinase structures. The betweenness of a node is defined as the number of shortest paths that pass through that node in the network, representing a global measure of the node contribution to the communication within the network. The residue centrality can characterize and differentiate highly connected residues that mediate stable interaction networks and allosteric communications in protein structures [94–99]. We also probed local residue environment using an energetics-based evaluation of relative solvent accessibility (RSA) [127,128]. A residue-specific RSA measure is defined as the ratio of the observed solvent-accessible surface area for a residue to the expected unfolded state value for the respective amino acid type [129].

**Fig 7:**
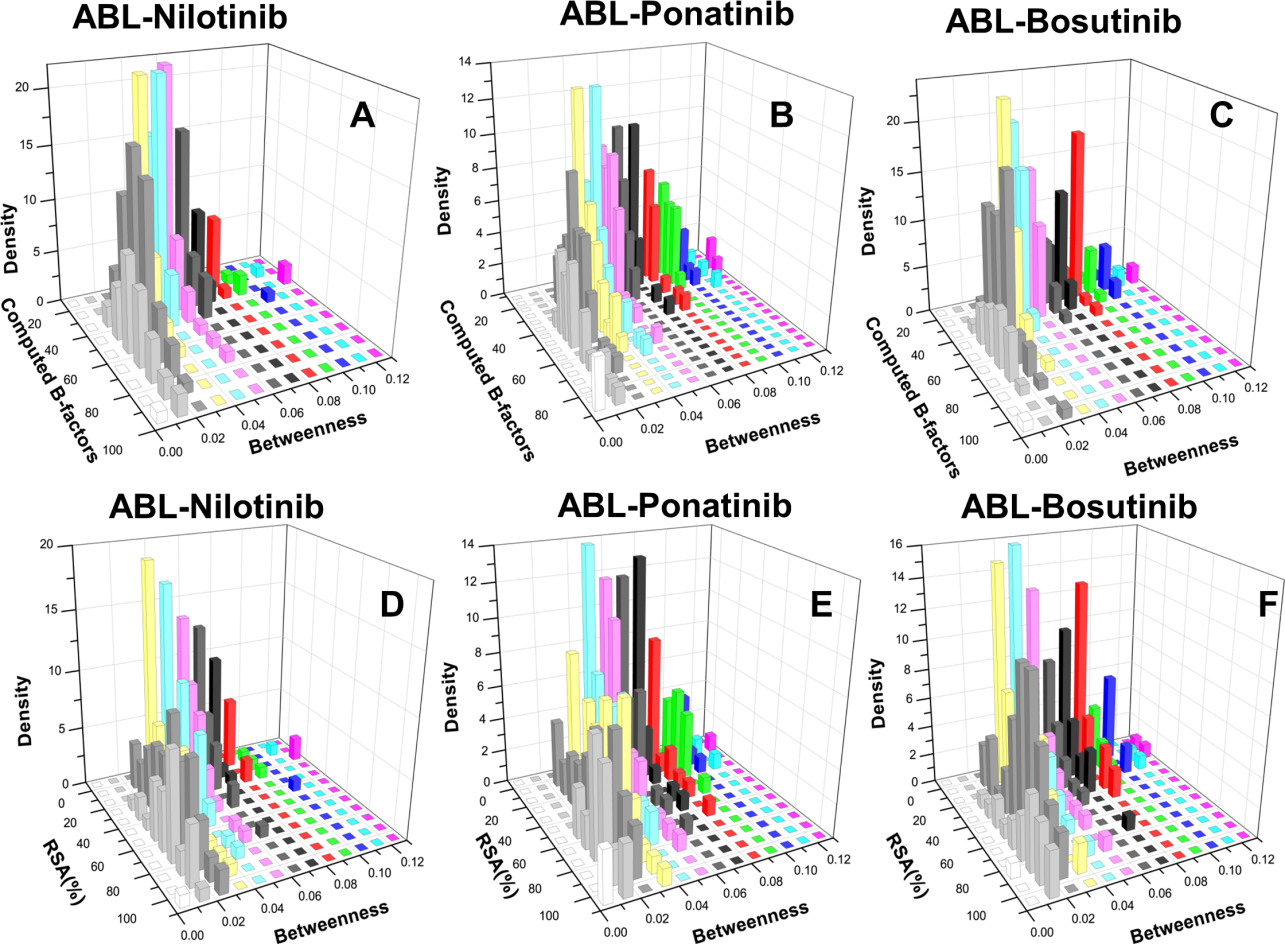
The Distributions of Residue Centrality in ABL Complexes. The joint distributions of residue-based centrality and computed B-factors are shown in the upper panel for ABL-Nilotinib complex (A), ABL-Ponatinib complex (B) and ABL-Bosutinib complex (C). In the lower panel (D-F), the joint distributions of residue centrality and relative solvent accessibility are respectively depicted for these kinase complexes.

We first examined the residue interaction networks of the ABL complexes with Nilotinib, Ponatinib and Bosutinib. For this analysis, we constructed joint distributions of the global residue betweenness and local residue flexibility measured by the computed B-factors (Fig 7A–7C), and residue-based RSA values (Fig 7D–7F). It could be seen that locally stable residues may display a broad range of centrality values (0.4–0.12), suggesting that only a small fraction of stable residues in the kinase core may serve as global hot spots mediating stability and allosteric interactions in the ABL kinase complexes. For the ABL-Nilotinib complex, we detected a small “isolated” cluster of high centrality residues that seemed to be separated from other residues (Fig 7D). This indicated that selective Nilotinib binding may be coupled with a small number of mediating residues. In some contrast, the residue interaction network of the ABL-Ponatinib complex revealed a denser distribution (Fig 7E). More specifically, Ponatinib binding may render a network where a significant number of residues (both buried and solvent-exposed) exhibited moderate-to-high centrality. This may render a more balanced connectivity of binding site residues that can reduce the inhibitor dependence on interactions with a few specific residues and thus promote a greater binding promiscuity. Although the crystal structures of Ponatinib and Bosutinib complexes with ABL kinase are radically different, the residue centrality distributions of these complexes were quite similar (Fig 7E and 7F). This indicated that common functional traits that dictate similar binding promiscuity of these inhibitors may be connected with the global networking properties.

**Fig 8:**
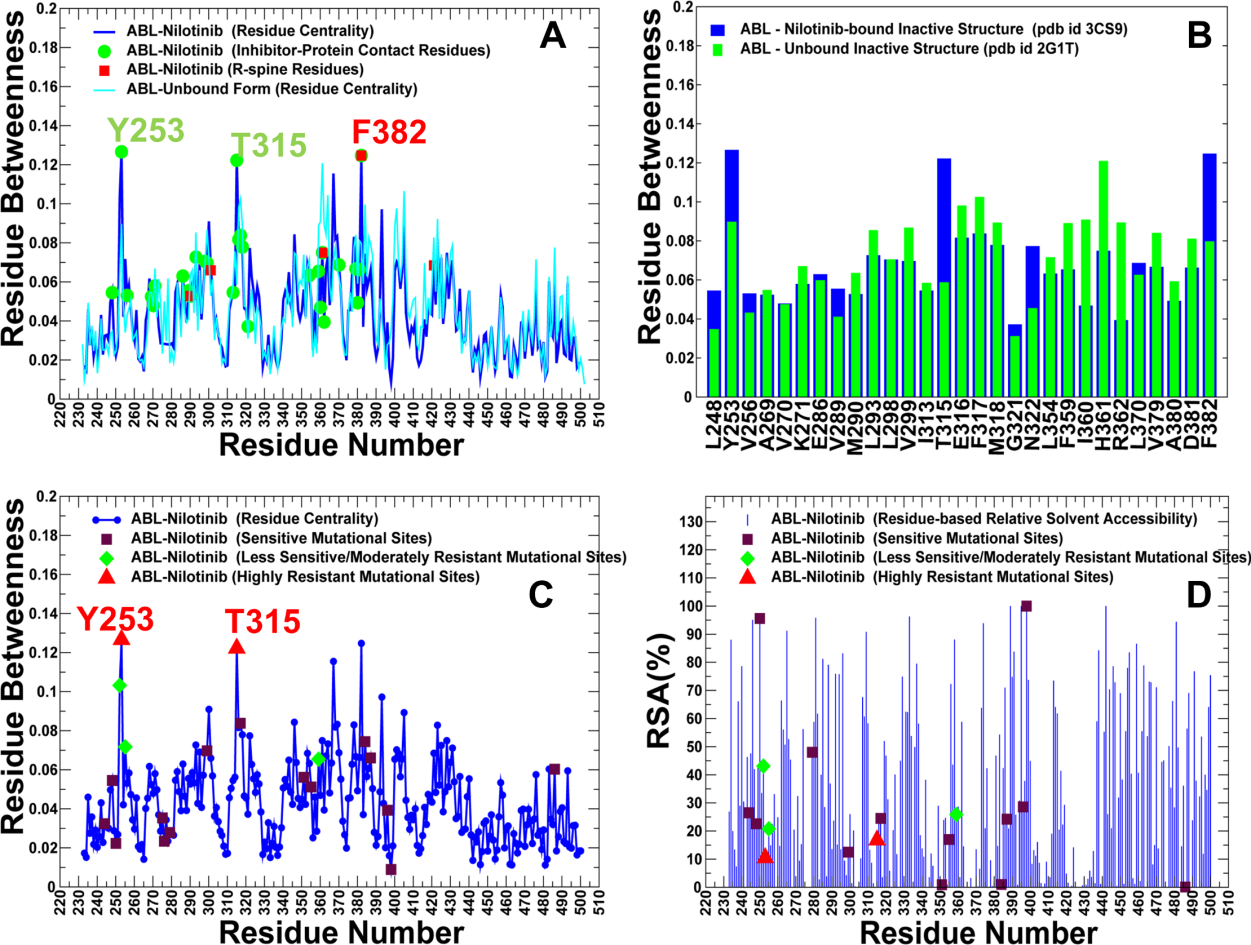
Analysis of the Residue Interaction Networks in the ABL-Nilotinib Complex. (A) The residue centrality profiles are shown for the apo ABL (in cyan) and inhibitor-bound inactive kinase form (in blue). The Nilotinib-interacting residues are shown in green circles and the R-spine residues are indicated by red squares. (B) A close-up of the ligand-induced changes in residue centrality of the binding site residues. The residue centrality values are shown for the unbound ABL structure (green filled bars) and for the Nilotinib-bound form (blue filled bars). A significant network-bridging effect of Nilotinib binding can be seen for Y253, T315, N322, L370, F382 residues that mediate stability of the inactive ABL structure. (C) Mapping of the Nilotinib-associated mutational sites on the residue centrality profile. These residues included positions of highly resistant Nilotinib mutations (Y253H/F, T315I/A), moderately resistant mutations (Q252H, E255K/V, F317L, F359C/V) and sensitive (non-resistant) mutations (M244V, L248V, G250E, D276G, E279K, V299L, M351T, E355G, L384M, H396R/P, G398R, F486S). The positions of highly resistant mutations are annotated in red upper triangles, moderately resistant mutations in green diamonds and non-resistant in maroon squares. (D) Mapping of the Nilotinib-associated mutational sites on the residue-based RSA profile. The annotation of mutational positions is the same as in (C).

To directly evaluate ligand-induced effects on the residue interaction networks, we examined the residue centrality profiles of the kinase structures in their apo and inhibitor-bound forms. For the ABL-Nilotinib complex, the binding site and R-spine residues corresponded to local maxima of the distribution, suggesting that these functional residues tend to have distinct networking characteristics (Fig 8A). We found that Nilotinib binding induced a sharply increased centrality for a subset of functional residues: Y253 (P-loop), T315 (gate-keeper), D363 (HRD motif), L370 (C-spine), R367, Y393 (phosphorylation site in the A-loop), and F382 (DFG motif) (Fig 8B). Some of these residues (R367, Y393) are not involved in direct contacts with the inhibitor, but are important for regulation and stability of the inactive kinase conformation. Importantly, several globally connected residues (Y253, T315, and F382) are also among energetic hot spots of Nilotinib binding (Y253, M290, T315, F317, F359, L370, and F382). In this mechanism, through direct contacts with the globally connected energetic hot spots, Nilotinib binding may affect allosteric interactions in the kinase domain and modulate stability of the residue interaction network in the inactive structure. According to our model, the enhanced centrality of these residues would strengthen cooperative interactions between the kinked P-loop (Y253), catalytic core (D393) and the unphosphorylated A-loop (Y393), that are central in promoting stabilization of the specific inactive state [54]. Selective Nilotinib binding could also enhance the stability and mediating capabilities of the R-spine (H361, F382) and C-spine residues (C369, L370, V371), which may additionally optimize allosteric communications between the N-terminal and C-terminal lobes in the inactive conformation. Overall, a pronounced network-bridging effect exerted by this selective inhibitor could stabilize structural environment favored by the inactive kinase form and thus shift thermodynamic preferences towards the specific kinase conformation.

We used the experimental data [67–69] that quantified changes in the inhibition constants of kinase drugs (Imatinib, Nilotinib, Ponatinib, Dasatinib, Bosutinib) against ABL kinase upon drug resistant mutations (S5 Fig) in our comparison with the computational results. In this analysis, we employed the nomenclature of resistant, moderately resistant and sensitive (non-resistant) mutations based on the experimental data and proposed classification [56]. A rigorous analysis of drug resistant effects would require conducting independent MD simulations for all studied mutants and their complexes. In the context of this study, we simplified the fundamental problem by suggesting that direct mapping of drug resistant sites onto the centrality profiles may help to differentiate the severity of mutations and provide a robust metric for assessing molecular source of resistance.

By mapping Nilotinib resistant sites onto the centrality profile, we found that this network parameter can distinguish between positions of highly resistant mutations (Y253H, T315I) and less sensitive or moderately resistant positions (Q252H, E255K/V, F359C/V) [56] (Fig 8C). In fact, highly resistant sites of Nilotinib binding corresponded to global mediating residues whose exceptionally high centrality is induced by the inhibitor binding. As a result, targeted mutations of these high centrality sites could disrupt allosteric coupling between functional regions, leading to the weakening and fragmentation of the residue interaction network. A strong network dependency on high centrality residues in the specific complex may explain vulnerability of Nilotinib binding to their targeted mutations and the emergence of drug resistance. It is worth noting that mutational mapping of the residue-based RSA profile (Fig 8D) was unable to distinguish between resistant and sensitive residues. In general, our results suggested that the severity of drug resistance mutations may be associated with the global mediating role of targeted residues.

**Fig 9:**
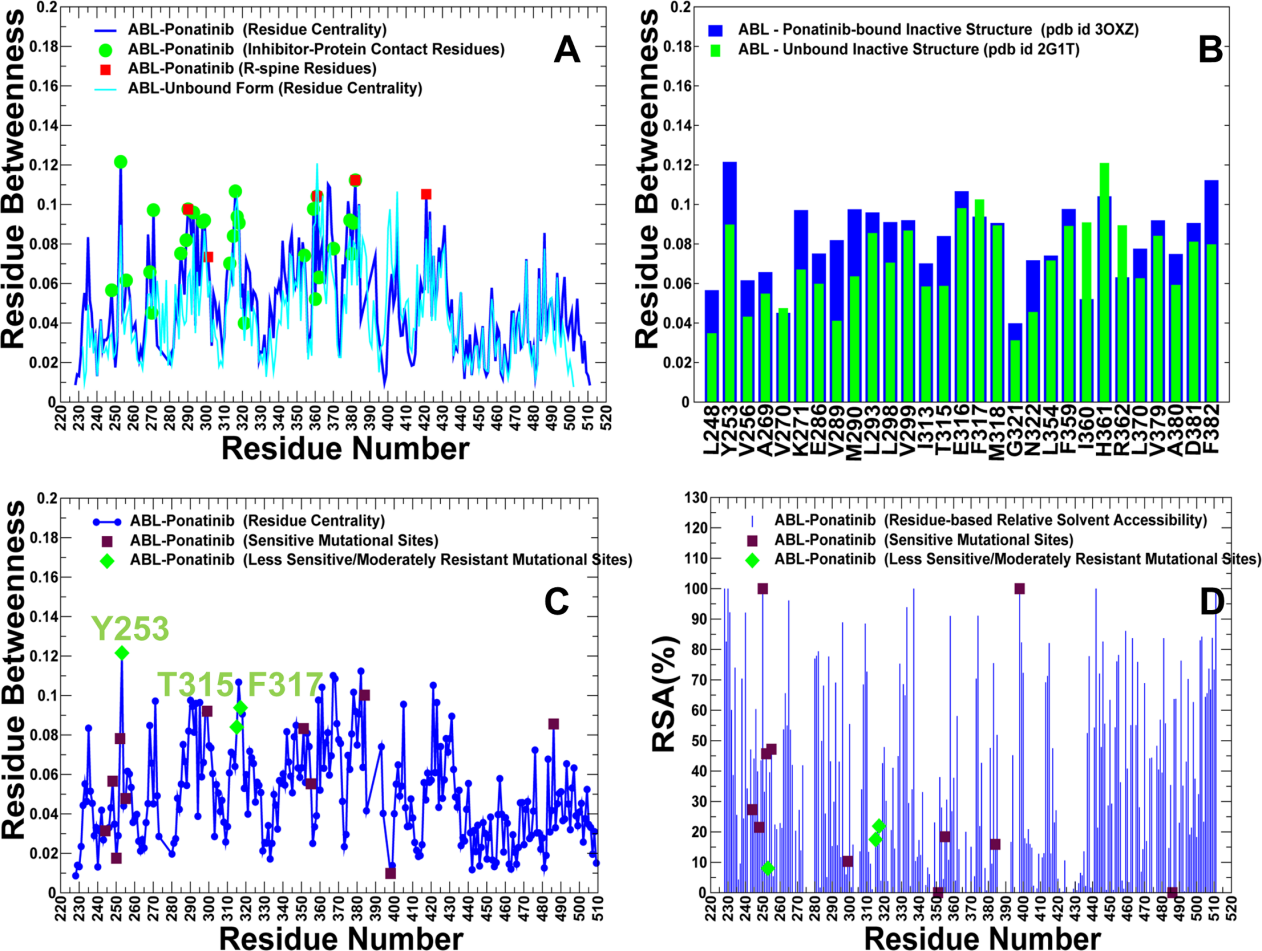
Analysis of the Residue Interaction Networks in the ABL-Ponatinib Complex. (A) The residue centrality profiles are shown for the apo ABL (in cyan) and inhibitor-bound inactive kinase form (in blue). The Ponatinib-interacting residues are shown in green circles and the R-spine residues are indicated by red squares. (B) A close-up of the ligand-induced changes in residue centrality of the binding site residues. The residue centrality values are shown for the unbound ABL structure (green filled bars) and for the Ponatinib-bound form (blue filled bars). (C) Mapping of the Ponatinib-associated mutational sites on the residue centrality profile. These residues included positions of moderately resistant Ponatinib mutations (Y253H/F, T315A, F317L), and sensitive (non-resistant) mutations (M244V, L248V, G250E, Q252H, E255K/V, V299L, M351T, E355G, L384M, G398R, F486S). The positions of moderately resistant mutations in green diamonds and non-resistant in maroon squares. The nomenclature of mutations is based on the experimental data and classification from [56]. (D) Mapping of the Ponatinib-associated mutational sites on the residue-based RSA profile.

Despite similar crystal structures and nearly identical inhibitor binding modes, the residue interaction networks in the ABL complexes with Nilotinib and Ponatinib complexes are different. The Ponatinib-induced changes in the residue centrality profile manifested in the increased betweenness for a significant number of kinase residues (Fig 9A). Most notably, the majority of the Ponatinib-interacting residues acquired higher centrality upon binding, leading to a broadly distributed and more uniform allocation of global mediating sites. The network-bridging effect of Ponatinib resulted in the increased centrality of many functional residues including Y253, K271, E286, M290, L298, V299, T315, F317, M318, D381, and F382 (Fig 9B). Accordingly, Ponatinib-induced reorganization of the interaction network could produce clusters of highly connected hot spots. Several distinct peaks of the centrality distribution (Y253, T315, and F317) also corresponded to the ABL residues associated with moderate Ponatinib resistance (Fig 9C). Other peaks represented functionally important residues interacting with Ponatinib that can preserve tumorigenic potential of the oncogenic protein [65]. In particular, mutations of E286, M318, and D381 can abolish kinase activity and compromise protein stability. Hence, the network organization of ABL-Ponatinib complex revealed the increased number and the broader connectivity of high centrality residues. These network signatures may reduce Ponatinib dependence on interactions with specific hot spot residues and facilitate greater promiscuity in binding with other kinase targets.

**Fig 10:**
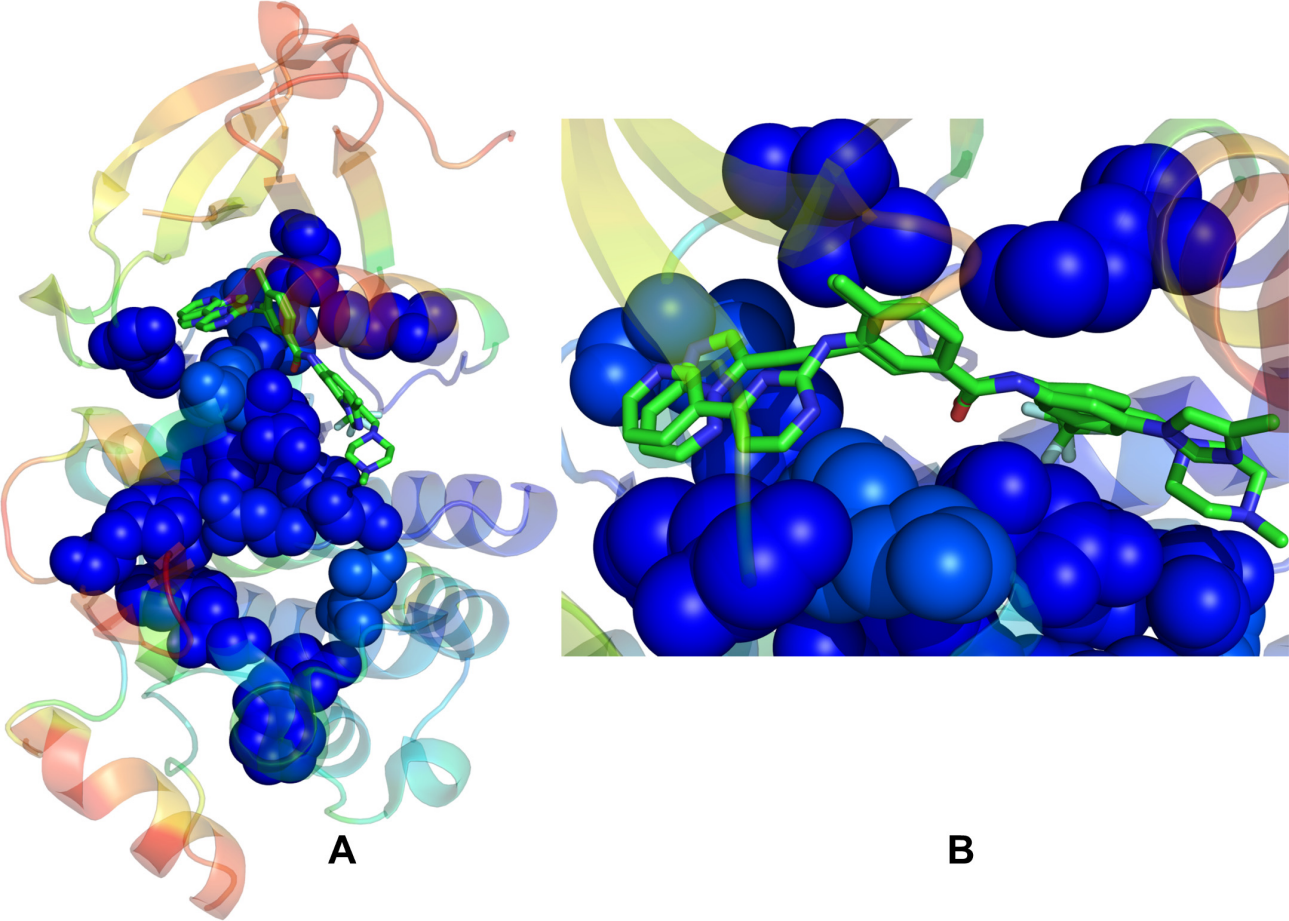
Structural Mapping of the Residue Interaction Networks in the ABL Complexes with Type 2 Inhibitors. (A) Structural mapping of residues corresponding to the peaks in the centrality profiles onto the inactive ABL structure (shown in blue spheres). The crystallographic binding modes of Nilotinib and Ponatinib are shown in sticks. The θ-like shape of the interaction network points to two lines of allosteric communication between the ATP binding site and the substrate binding regions. (B) A close-up of the inhibitor binding modes shows coupling of the type 2 inhibitors to high centrality sites in the specific inactive structure.

To illustrate how binding of type 2 inhibitors is coupled with the residue interaction networks, we mapped residues corresponding to the peaks of the centrality profile onto the inactive ABL structure (Fig 10). The θ-like shape of the interaction network provided two lines of communication that connected the ATP binding site and the substrate binding regions (Fig 10A). Strikingly, the C-spine and R-spine represent subnetworks of the global interaction network of high centrality sites. Indeed, one side of the network could be traced from the active site to the αD-helix through the C-spine residues to the integral αF-helix and the substrate binding region. Another line of communication linked the ATP binding site with the αC-helix and via the R-spine residues to the substrate region. The θ-shaped residue network is rested on allosteric coupling between two lines of communications that is provided by structurally rigid and strategically positioned H361, D363, and Y393 residues. High centrality residues that mediate structural stability in the kinase structures often correspond to functionally important sites involved in the kinase activity, regulation and binding.

We observed that selective Nilotinib binding tends to maximize complementarity and allosteric coupling with the global interaction network (Fig 10B). Nilotinib binding is coupled with the global interaction network via direct contacts with the energetic hot spots T315, M318, L370, D381, F382, and H361 residues. The interaction network of these high centrality sites may also act as a direct and optimal path for transmitting allosteric signals between functional kinase regions. By targeting the energetic hot spots and increasing centrality of these global mediating residues, Nilotinib binding may optimize the residue interaction networks and induce further stabilization of the inactive ABL conformation. Remarkably, relatively minor structural deviations in the binding mode of Ponatinib may trigger global changes in the ligand coupling with the residue interaction network that alter the binding specificity profile. Our analysis detected a weakened coupling of Ponatinib to key mediating residues T315 and F382 that control access to the hinge region and conformational preferences of the DFG regulatory motif (Fig 10B). Collectively, these subtle changes may alleviate inhibitor dependency on the specific kinase conformation of ABL kinase and switch inhibitor preferences towards more promiscuous binding.

**Fig 11:**
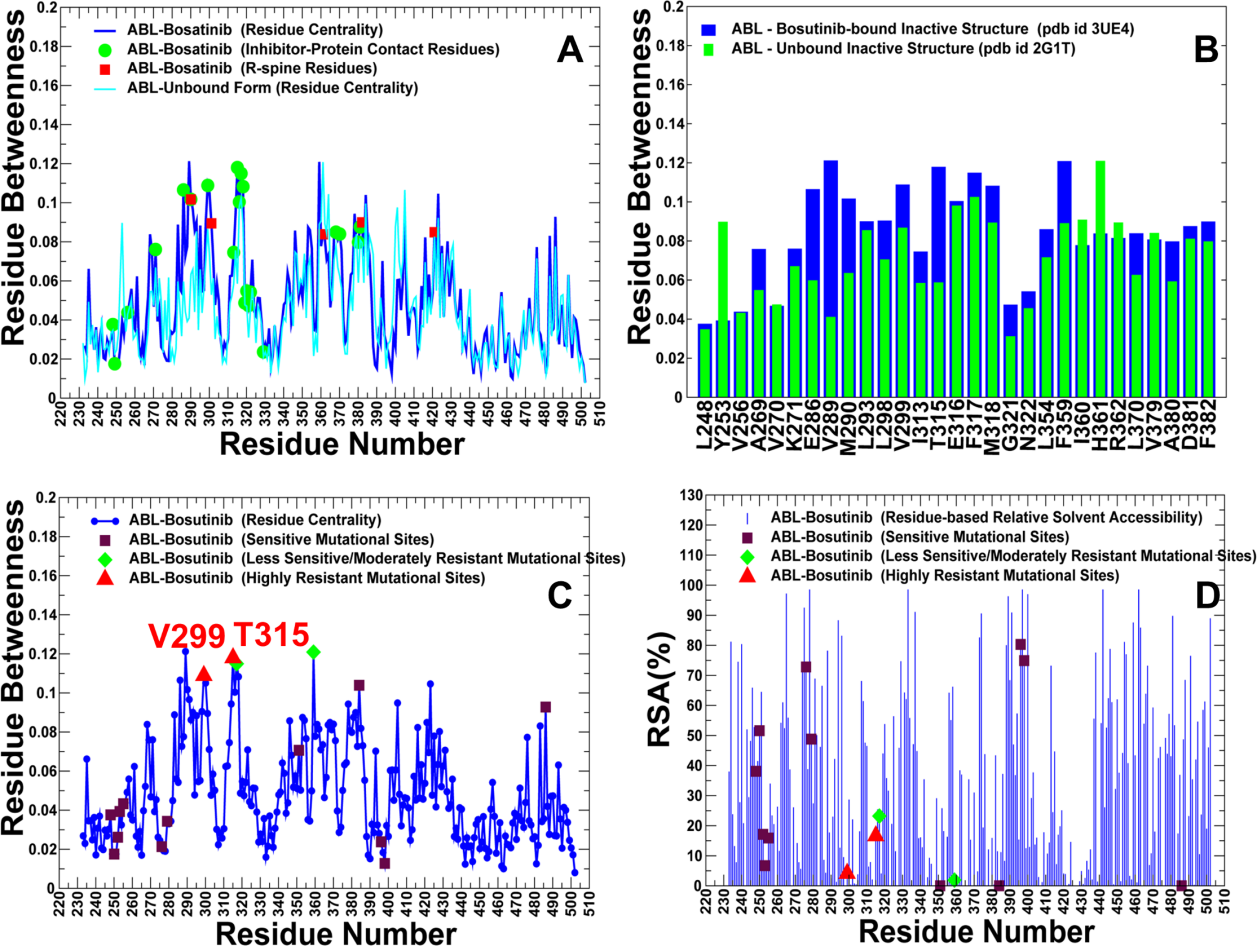
Analysis of the Residue Interaction Networks in the ABL-Bosutinib Complex. (A) The residue centrality profiles are shown for the apo ABL (in cyan) and inhibitor-bound DFG-out kinase form (in blue). The Bosutinib-interacting residues are shown in green circles and the R-spine residues are indicated by red squares. (B) A close-up of the ligand-induced changes in residue centrality of the binding site residues. The residue centrality values are shown for the unbound ABL structure (green filled bars) and for the Bosutinib-bound form (blue filled bars). A network-bridging effect of Bosutinib binding is noticeable for residues in the αC-helix region (E286, V289, M290), and hinge region (T315, F317, M318). (C) Mapping of the Bosutinib-associated mutational sites on the residue centrality profile. These residues included positions of highly resistant Bosutinib mutations (V299L, T315I), moderately resistant mutations (F317L, F359V), and sensitive (non-resistant) mutations (L248V, G250E, Q252H, Y253F/H, E255K/V, D276G, E279K, M351T, L384M, H396R/P, G398R, F486S). The positions of highly resistant mutations are annotated in red upper triangles, moderately resistant mutations in green diamonds and non-resistant in maroon squares. The nomenclature of mutations is based on the experimental data and classification from [56]. (D) Mapping of the Bosutinib-associated mutational sites on the residue-based RSA profile. The annotation of mutational positions is the same as in (C).

We also examined ligand-induced changes in the centrality profiles for the kinase complexes with type 1 inhibitors. The betweenness profile of the Bosutinib-ABL complex revealed the increased centrality of the αC-β4-loop/αC-helix region and hinge residues that correspond to the major distribution peaks (Fig 11A). The network bridging effect appeared to be significant only in these sites, which is consistent with the notion that structural stability of the αC-β4-loop/αC-helix (E286, V299, M290) and the hinge region (T315, F317) are vital for the active ABL conformation. The close-up of the centrality profile further highlighted the network-bridging effect in E286, V289, M290, V299, T315, and F359 residues, indicating that the assembly of the R-spine and active position of the αC-helix residues are central in mediating stability of the Bosutinib complex (Fig 11B). These residues also included Bosutinib resistant sites (V299, T315, F317, and F359), showing that residue centrality could differentiate between inhibitor-resistant and inhibitor-sensitive residues (Fig 11C). Hence, ligand-induced network bridging effects may be linked with binding sensitivities of Bosutinib towards mutational variants of ABL kinase.

**Fig 12:**
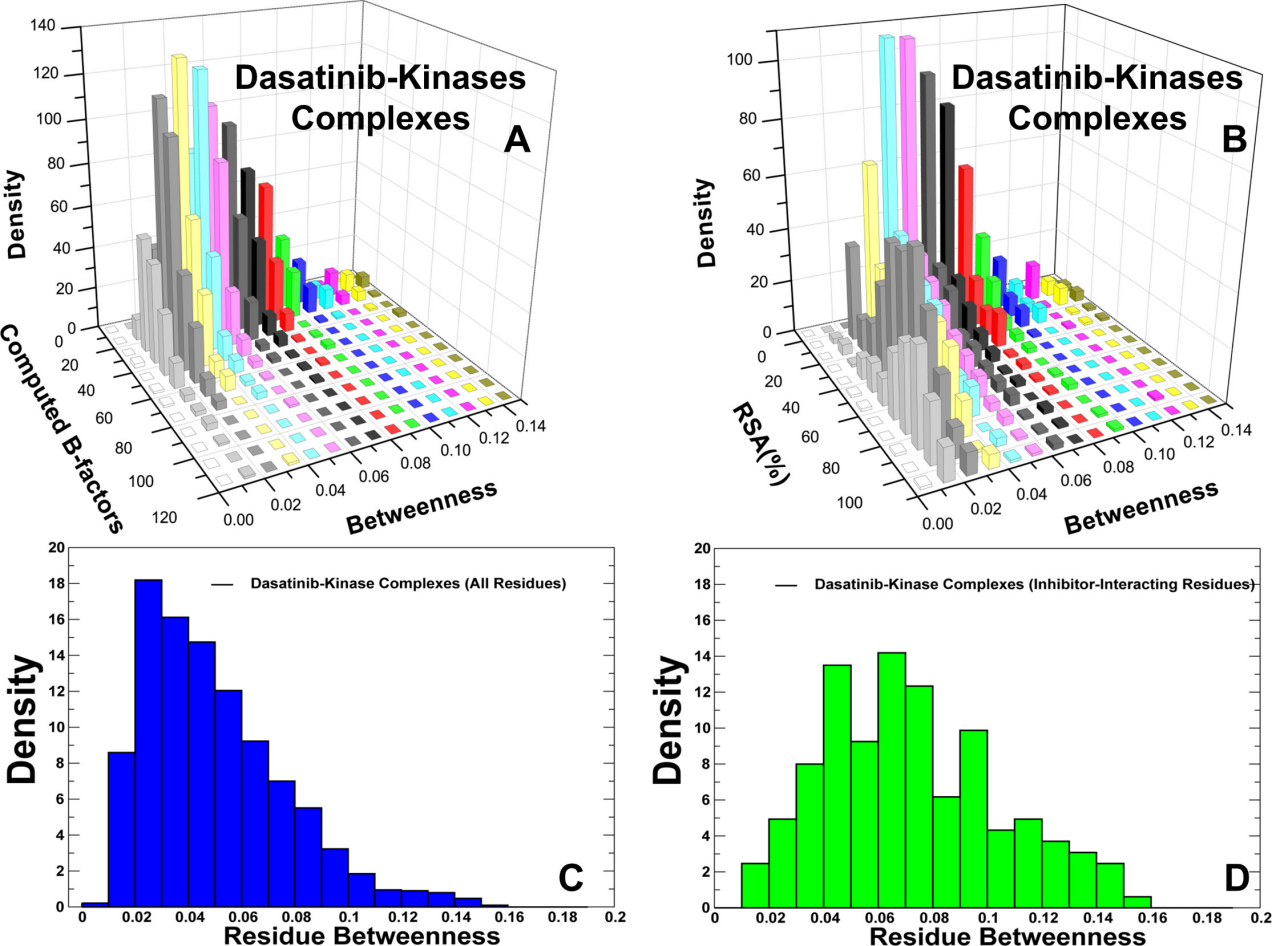
Analysis of the Residue Interaction Networks in Dasatinib-Kinase Complexes. (A) The joint distributions of residue-based centrality and computed B-factors. (B) The joint distributions of residue centrality and relative solvent accessibility. (C) The probability density function of residue centrality (betweenness) values obtained from MD simulations of Dasatinib-kinase complexes. (D) The probability density function of residue centrality (betweenness) values for Dasatinib-interacting residues.

Using MD simulations of Dasatinib complexes with different kinases, we aggregated the results of network analysis and analyzed coupling between residue betweenness and local structural parameters (Fig 12A and 12B). While low centrality residues are typically flexible and solvent-exposed, we detected a dense area of the medium-betweenness residues that have a similar degree of local mobility as the high centrality sites. The centrality distribution density revealed the characteristic small-world topology, in which the high centrality residues in Dasatinib complexes formed a relatively small population (Fig 12C). The number of highly connected central nodes seemed to exponentially decay as the residue centrality increased. In agreement with network-based studies of protein structures [93], the distribution of global mediating residues may obey the Poisson model, as the residue nodes that significantly deviated from the average degree were rare. We also mapped out the centrality distribution of the Dasatinib-interacting residues that is shallow and shifted towards the low and medium betweenness values (Fig 12D). It implied that the network-bridging effect of Dasatinib binding was relatively minor since most of the binding site residues displayed an average betweenness similar to the rest of the kinase molecule. The observed effect was uniform across all Dasatinib-kinase complexes (S6 Fig). Notably, the only energetic hot spots with high centrality were the conserved residues in the hinge region (T315 and M318 in ABL) and C-spine (L370 in ABL). Hence, binding sensitivities of Dasatinib may be primarily controlled by the identity and structural conservation of the energetic hot spots in the hinge region (gate-keeper T315 and M318), and could be also coupled to the C-spine network (via interactions with L370). The results are consistent with recent observations pointing to strong evolutionary conservation of the gate-keeper residue in the kinases that bind Dasatinib, since among 94 kinases with the conserved threonine at the gatekeeper position ~60 kinases could be targeted by Dasatinib [39].

**Fig 13:**
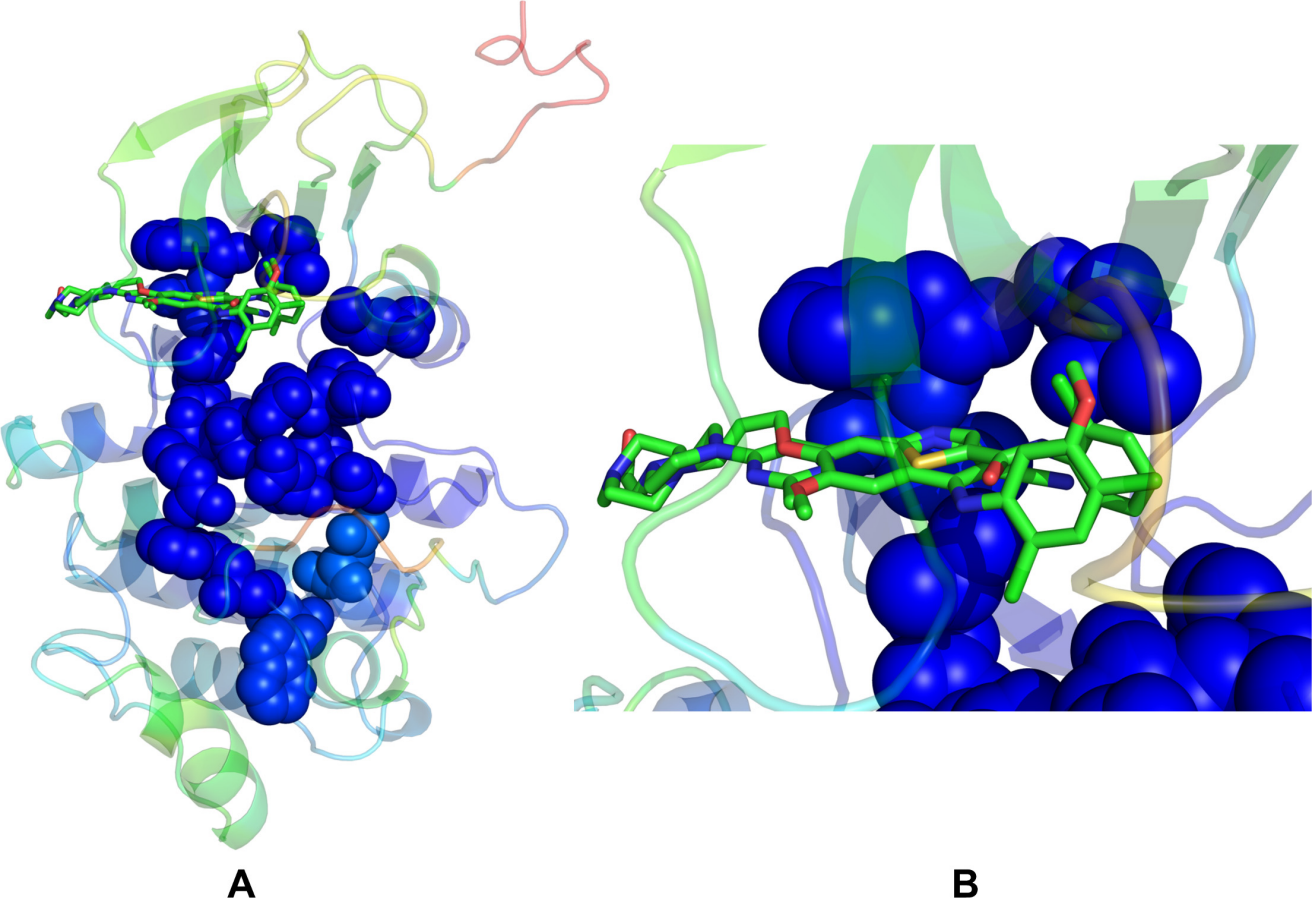
Structural Mapping of the Residue Interaction Networks in the Kinase Complexes with Type 1 Inhibitors. (A) Structural mapping of residues corresponding to the high centrality sites in Bosutinib and Dasatinib complexes onto the active ABL structure (blue spheres). The crystallographic binding modes of Bosutinib and Dasatinib are shown in sticks. (B) A close-up of the binding modes shows coupling of the type 1 inhibitors to high centrality sites in the active kinase structure.

We also mapped residues corresponding to the centrality peaks in the complexes with type 1 inhibitors onto the active ABL conformation (Fig 13). The key functional residues occupy strategic central positions in this network including catalytic residues (K271 and E286), T315, M318 (hinge region), HRD and DFG motifs as well as the substrate binding motif 405-WTAPE-409. The θ-like shape of the residue interaction network appeared to be a conserved feature of the inactive and active kinase structures. In both cases, the C-spine and R-spine residues contribute to the global interaction network. Noteworthy is coupling of Dasatinib binding with the subnetwork that links the binding site via the C-spine to the αF-helix, and the substrate binding region (Fig 13). At the same time, Dasatinib binding may be more tolerant to conformational variations in the other communication line that connects the active site with the R-spine and DFG residues. Hence, binding of type 1 inhibitors can incur relatively moderate network changes and allow for a greater conformational tolerance of the active kinase states.

### The Mutual Information Network of Coevolving Residues and Binding Sensitivities of the Kinase Inhibitors

**Fig 14:**
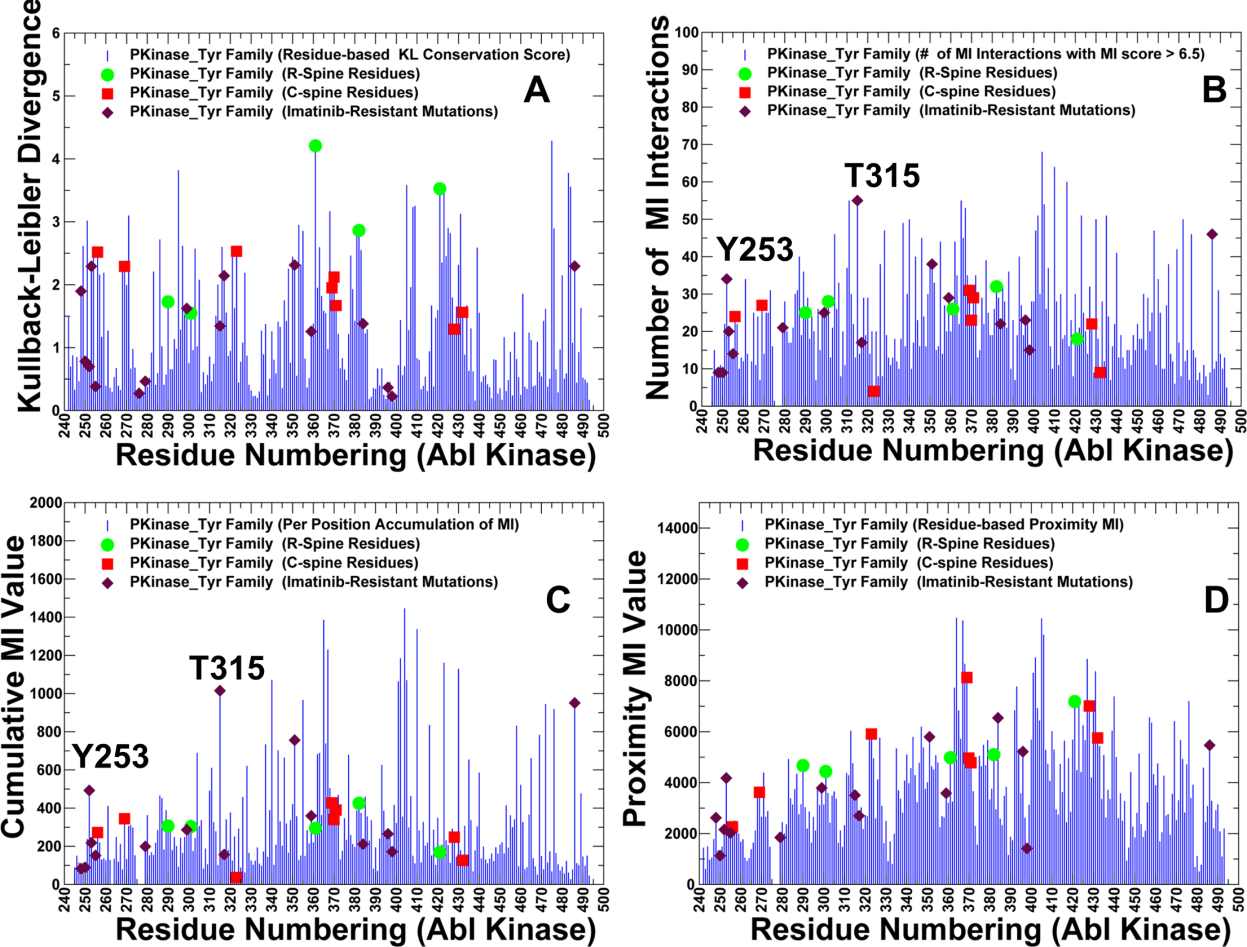
Analysis of Coevolving Residues in Protein Tyrosine Kinases. (A) The residue-based Kullback-Leibler conservation score; (B) The number of coevolving residue interactions per residue; (C) The residue-based cMI score; (D) The residue-based pMI score. The coevolutionary parameters were calculated using MISTIC approach [130]. The coevolutionary parameters were mapped onto ABL kinase and residue annotation is consistent with residue numbering in ABL complexes. The coevolutionary parameters are highlighted for the R-spine residues (green circles), C-spine residues (red squares), and Imatinib-resistant mutations (maroon diamonds).

In this section we investigated how binding preferences of the kinase inhibitors may be linked with the networks of coevolving residues in the kinase structures. Prediction of strongly coevolving residue pairs can help to clarify molecular mechanisms underlying kinase function and binding. We characterize coevolved residue positions that are associated with conformational stability, catalytic and ligand binding function. The underlying premise of this analysis is that strongly coevolving kinase residues are often located near active sites and coevolve with many other residues, thus forming interaction clusters that modulate stability of the kinase conformations. We constructed mutual information networks of coevolving residues in protein tyrosine kinases and mapped residues with the high coevolutionary signal onto ABL kinase structures. In this analysis, we compared the organization and topology of the coevolutionary residue network and the residue interaction network. According to our hypothesis, a subset of kinase residues exhibiting strong coevolutionary signal may be also associated with high centrality and correspond to energetic hot spots of kinase inhibitors. By using mutual information (MI) approach [130] we evaluated coevolutionary relationships between position pairs in the tyrosine kinase family. This provided a sufficient accuracy of the sequence analysis and identified positional correlations that guided subsequent structural mapping onto the kinase conformations and analysis of functionally important residues. We computed residue-based MI score that characterizes the extent of mutual information shared by a given residue with other protein residues. Based on MI scores and using MISTIC server [130], a network of coevolutionary residues was constructed where nodes are residues and links between nodes represent a coevolutionary signal between residue pairs. To analyze the network of coevolving residues, we explored the following parameters: the Kullback-Leibler conservation score (Fig 14A), the number of coevolving residue interactions per residue (Fig 14B), a cumulative mutual information score (cMI) for each residue (Fig 14C), and the proximity mutual information score (pMI), which measures the mutual information shared by the residues in the proximity of a given residue within a certain distance threshold (Fig 14D).

**Fig 15:**
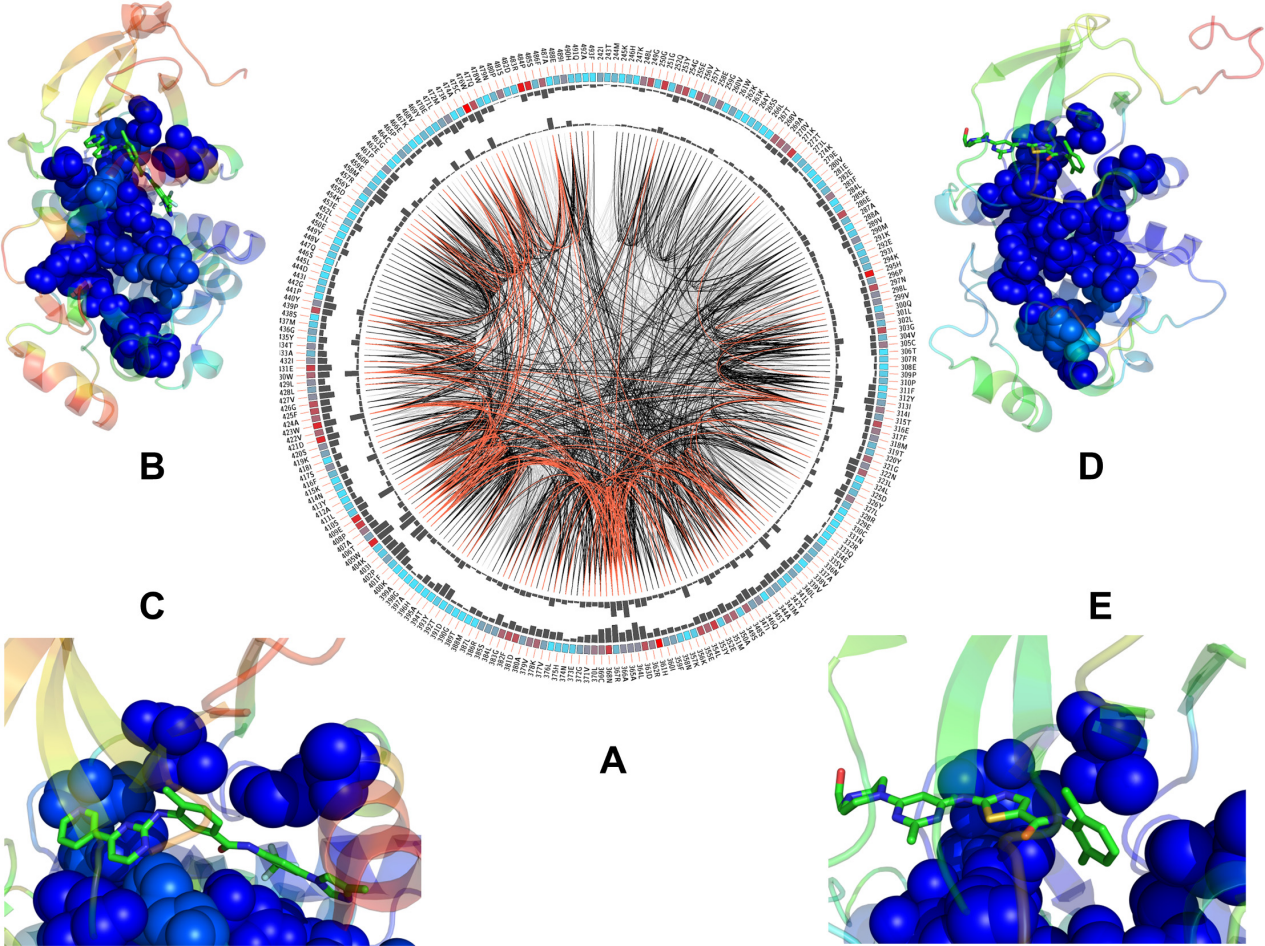
The Network Analysis of Coevolving Residues: Residue Connectivity and Structural Mapping onto Kinase Structures. The network connectivity of coevolving residue pairs is shown by a sequential circular representation of the MSA. The labels in the first (outer) circle indicate the alignment position and the amino acid code of the reference sequence. The colored square boxes of the second circle indicate the MSA position conservation (highly conserved positions are in red, while less conserved ones are in blue).The third and fourth circles show the pMI (pMI) and cMI values as histograms, facing inwards and outwards respectively. Each node represents a position in the MSA and lines between nodes in the circle connect pairs of positions with MI > 6.5 as defined by MISTIC program [130]. Node color represents site conservation: red for highly conserved sites and blue for less conserved sites.

The conservation score (Fig 14A) and cMI profile (Fig 14C) were fairly similar, both revealing that highly coevolving residues were primarily localized in the catalytic loop (residues 357–372 in ABL) and substrate binding site (residues 399–416 in ABL). This loop includes the critical 361-HRD-363 motif that is involved in kinase activity and regulation. The catalytic loop sequence 363-DLAARN-368 is also highly conserved throughout the majority of protein tyrosine kinases and includes a critically important for kinase activity R367 [131,132]. A strong correspondence between coevolving residue positions and conserved residues in the catalytic and substrate binding sites is consistent with the previous studies [112–123]. The pMI profile evaluated the propensity to form modules of coevolving residues, which revealed that dense clusters tend to be formed in the proximity of the catalytic and substrate binding sites (Fig 14D). Hence, residues that are functionally critical for kinase activity coevolve and coevolutionary relationships often include pairs of residues from allosterically communicating active sites. Moreover, catalytic residues and their structural neighbors tend to coevolve with each other and form independent structural modules stabilized by the physical interactions [120,121]. To facilitate functional analysis of coevolving residues, we also mapped positions of the R-spine and C-spine residues onto MI profiles (Fig 14). The network connectivity of the coevolving residue pairs is illustrated by a sequential circular representation of the MI scores (Fig 15). The connections in the center of the circle link pairs of positions with significant MI score. We also mapped highly coevolving groups of residues onto the inactive and active kinase structures to explore a relationship between organization of these networks and inhibitor binding (Fig 15).

The rigid R-spine residues H361 (HRD), D382 (DFG), and D421 (αF-helix) represented conserved and highly coevolving positions in all four distributions (Fig 14). Similarly, highly coevolving C-spine residues are either proximal to the catalytic region (L370, V371) or belong to the highly conserved αF-helix (L428, I432). The high conservation of the αF-helix stems from its central role in anchoring the R-and C-Spines subnetworks with the rest of the kinase core. Notably, the decisive role of the R-spine residues was especially striking in the conservation score (Fig 14A) and pMI distributions (Fig 14D), showing that modules of highly coevolving residues can link the highly conserved and rigid catalytic residues with the more flexible residues (such as R-spine M290 and L301 from the αC-helix) that facilitate conformational changes in the kinase domain. The pMI profile revealed clusters of coevolving residues in the ATP binding site, catalytic loop, the αF-helix, and the substrate binding site that have distinguishable network signatures and may have evolved independently (Fig 14D). This finding is consistent with the notion that most of the coevolving residues are spatially proximal in the protein structures and tend to form relatively independent protein sectors of functional residues [103,104,117,118]. However, the dense network connectivity demonstrated that coevolved clusters could emerge at different distance regimes, including coupling between distant functional modules such as the ATP binding site (hinge residues 313–322), αC-helix (residues 279–292), and A-loop (residues 378–415) (Fig 15). The appearance of coevolutionary clusters that include mobile residues in the αC-helix and A-loop is likely associated with dynamic requirements for global rearrangements. In particular, we observed that modules of coevolving residues can connect the ATP binding site residues with the αC-β4-loop/αC-helix hinge region and DFG motif of the A-loop. The formation and disruption of residue interaction networks near the hinge site is a significant driver of conformational transitions between the kinase states.

The network of coevolving residues presented a characteristic θ-shaped topology, when projected onto the kinase structures, with two routes linking the ATP binding sites and the substrate binding via the R-spine and C-spine (Fig 15). We observed that high centrality and highly coevolving kinase residues often coincide and correspond to the conserved functional sites mediating stability and allosteric communications in the kinase domain. These residues included the gate-keeper T315 from the ATP site, HRD motif of the catalytic loop, and substrate binding motif 405-WTAPE-409 in the C-lobe. Most notably, the coevolutionary distributions pointed out to a group of residues that may simultaneously display a strong coevolutionary signal, high centrality and present a binding hot spot for the ABL kinase inhibitors (Figs 14 and 15). In particular, high centrality Y253 and T315 residues showed high conservation and cMI score as well as a large number of coevolving interactions with other residues in different regions (Fig 14A–14C). Several other global mediating residues (H361, F382) exhibited similarly strong coevolutionary signatures. The integrating role of these residues in coevolutionary networks and residue interaction networks necessitates their high rigidity, which is determined by catalytic and structural stability requirements. Hence, binding preferences of Nilotinib may arise from energetic coupling with rigid networks of these highly connected residues that define stability of the specific kinase states (Fig 15B and 15C). Among energetic hot spots of Dasatinib binding, the gate-keeper T315 was the only functional residue that displayed a markedly high centrality in interaction networks and exceptionally high coevolutionary propensity (Fig 14). Accordingly, Dasatinib coupling with the networks of coevolving and interacting kinase residues may be almost exclusively controlled by the identity and structural stability of the gate-keeper residue. As a result, structural stability requirements for productive Dasatinib binding may be relatively relaxed and rely primarily on rigidity of the hinge region near the gate-keeper residue and integrity of the C-spine architecture. Mapping of coevolutionary residues onto the active ABL structure illustrated how Dasatinib can communicate with this network (Fig 15D and 15E). The inhibitor is coupled with the hinge region and a subnetwork that links the ATP binding site with the substrate region via the C-spine. We observed that important drug resistant sites could be identified based on high coevolutionary scores and a large number of coevolving residue interactions. Of special interest, the emergence of Y253 and T315 residues as highly coevolving and high centrality positions in the network distributions. This confirms the unique role of the P-loop and the gate-keeper residue in modulating kinase activity and binding. The vulnerability of the interaction networks in the ABL complexes to targeted perturbations of these residues may explain why T315I mutation and P-loop mutations could leverage a local perturbation to switch the global conformational equilibrium away from the specific inactive conformation and stabilize the active form [133].

## Discussion

In this work, we have introduced a computational framework for explaining binding preferences of the ABL kinase inhibitors by unraveling a relationship between energetic hot spots of ligand binding and organization of the residue interaction networks in various kinase states. The approach is rooted in the energy landscape theory and invokes conformational selection mechanism, according to which the relative populations of preexisting kinase states can be modulated by inhibitor binding through selection and preferential stabilization of a particular functional form [134–136]. The main assertion of this study was that kinase inhibitors could intervene into organization of the residue interaction networks through binding hot spots that also function as global mediating sites in protein kinase structures. In this mechanism, by using local perturbations in the binding site, selective inhibitors may facilitate a global population shift of conformational ensembles towards a specific bound form. Structure-based network analysis has recapitulated various experimental observations by linking residue centrality and structural stability of the residue interaction networks with kinase-specific binding preferences to drug binding.

Modeling of the residue interaction networks and networks of coevolving residues in the ABL-drug complexes has determined a group of conserved functional sites that can simultaneously embody strong coevolutionary signal, high centrality, and the propensity to be energetic hot spots of inhibitor binding. These residues corresponded to functionally critical sites that regulate kinase activity, determine protein structure stability and control inhibitor binding. By probing ligand-induced changes in the residue interaction networks, we have found that inhibitor binding can change the local connectivity of the binding hot spot residues and rewire the global interaction network in the kinase complexes. In this mechanism, binding of selective inhibitors, such as Imatinib and Nilotinib, could enhance centrality of the hot spot residues that mediate structural environment favored by the specific inactive state. More promiscuous inhibitors Bosutinib and Dasatinib appeared to impose modest changes in the network organization, in which ligand binding is strongly coupled only with the identity and stability of the gate-keeper residue. These findings may have interesting implications, particularly in light of recent experiments suggesting that selectivity of kinase inhibitors is not associated with a specific type but may rather result from targeted exploitation of the kinase conformational landscape [55].

The results of this study may have a particular relevance in relating differences in the network organization of kinase complexes with drug resistance effects. A strong relationship between residue positions subjected to drug resistant mutations and their unique topological signatures emerged from the analysis of the residue interaction networks and networks of coevolving residues. We have found that residue centrality may be used as a robust metric for assessing severity of drug resistance mutations and differentiating between highly resistant and moderately resistant positions. Although many highly coevolving kinase residues can be associated with drug resistance, coevolutionary relationships could often hinder subtle differences in mutational sensitivities of binding site residues in response to drug binding. A consensus analysis that identifies energetic hot spots exhibiting both high coevolutionary and high centrality scores in the residue networks may present a simple and potentially more useful strategy to quantify drug resistant and disease-associated positions in protein kinases.

A combination of molecular simulations and network approaches could provide a useful perspective on structure and energetics of ligand binding via lenses of network efficiency and robustness to external perturbations and mutations. Systems properties such as modularity, bow-tie architectures, degeneracy and other topological features are often associated with robustness of protein systems [137]. Robustness is also supported through functional redundancy and response diversity. Our study has shown that selective and promiscuous inhibitor binding can be interrelated with the robustness of the residue networks in kinase structures. A resilient protein structure network should be able to cope with random and targeted attacks by providing efficient connectivity and interaction cooperativity required for proper function and signaling. According to our findings, binding selectivity in the ABL kinase complexes may be associated with the emergence of specific high betweenness nodes that can rapidly transmit allosteric signal in the network. However, this may result in the reduced robustness of the system and the increased dependency on a small number of critical mediating residues, making specific drug binding vulnerable to targeted mutations. In network terms, drug resistant mutations in ABL may be regarded as a protein response to regain its robustness against the drug through diversity of mutations that maintain the activity of tumor cells [137].

Robustness of complex systems may be drastically improved by either rewiring a fraction of the edges or adding a modest number of new edges at the suitable location of the network [138–140]. It has been discovered that a limited rewiring can create robust networks in which highly connected nodes are localized in the core surrounded by interconnected layers of less connected nodes [141]. This can improve network resilience by redistributing the network load among many high betweenness nodes without compromising the network topology and decreasing robustness against random failures [141,142]. Considering protein targets as robust interaction networks exhibiting points of fragility may provide insights for the development of new drugs using systems-based approaches [143,144]. The association of network properties with kinase regulation and binding suggests that residue interaction networks may be reorganized and specifically tailored through therapeutic intervention that is informed by knowledge of high centrality residue nodes. Structure-based network approach may thus provide a useful framework for rational design of therapies that target less essential nodes to increase synergetic performance and decrease side effects [145,146].

System-based approaches to drug discovery, such as network pharmacology, have exploited advances in chemical biology and network science to develop strategies for multitarget drugs and targeted drug combinations in the context of structural and biological networks [147–149]. These combinatorial therapies are considered as a promising alternative to combat drug resistance and side effects that arise from compensatory mechanisms and robustness of cellular systems to external perturbations. Rational design of drug combinations and multi-target drugs requires development of novel theoretical and experimental approaches that could bridge microscopic analysis of protein structure networks with macroscopic modeling of cellular networks and signaling pathways [137]. Dissecting the relationships between protein robustness, specificity, and drug binding within a unified framework of protein structure networks may prove to be a useful step in this direction.

## Materials and Methods

### MD Simulations and PCA Computations

Combinations of 200 ns MD simulations were carried out for the crystal structures of the kinase-inhibitor complexes studied in this work. For each structure, starting from the same initial conditions, we performed a single 200 ns simulation and 4 independent 50 ns simulations. The collected statistics used in the computational analysis was based on all MD trajectories. The following crystal structures were simulated: the inactive ABL structure bound with Nilotinib (pdb id 3CS9) and Ponatinib (pdb id 3OXZ); the active-like ABL structure bound with Bosutinib (3UE4), the active SRC structure bound with Bosutinib (pdb id 4NMXO); Dasatinib complexed with the active structures of EPHA4 (pdb id 2Y6O); LYN (pdb id 2ZVA), P38 (pdb id 3LFA), BTK (pdb id 3K54); Dasatinib binds with the inactive structures of BMX (pdb id 3SXR) and BTK kinases (pdb id 3OCT). An active DFG-in conformation is adopted in Dasatinib complexes with ABL, SRC, EPHA4, LYN, P38, and BTK kinases (pdb id 3K54). Dasatinib binds to a DFG-out conformation of the inactive BMX kinase (pdb id 3SXR). In the complex with an inactive BTK conformation (pdb id 3OCT) an intermediate DFG position is adopted which is between the fully DFG-in and DFG-out conformations. All crystal structures were obtained from the Protein Data Bank (RCSB PDB www.rcsb.org) [150]. All crystallographic water molecules were removed and missing hydrogen atoms of the protein were added. All ionizable residues were considered in the standard ionization state at neutral pH condition. The missing residues, unresolved structural segments and disordered loops were modeled and evaluated with the ModLoop server [151,152] and the ArchPRED server [153]. The unresolved portions of the P-loop and A-loop in the kinase complexes were assembled and energetically refined using the ArchPRED server.

MD simulations were carried out using NAMD 2.6 [154] with the CHARMM27 force field [155,156] and the explicit TIP3P water model. The employed MD protocol is consistent with the overall setup described in details in our earlier studies [157]. The initial structures were solvated in a water box with the buffering distance of 10 Å. MD simulations were run in the NPT ensemble at 1atm and 300K using extended system pressure algorithm [158] and Nosé-Hoover thermostat [159, 160]. Long-range nonbonded van der Waals interactions were treated using an atom-based cutoff of 12Å with switching van der Waals potential beginning at 10Å. The smooth particle mesh Ewald (PME) method [161] was employed to treat the long-range electrostatics. Numerical integration was performed using the leap-frog Verlet algorithm with 2fs time step [162]. Covalent bond lengths involving hydrogen were constrained using the SHAKE algorithm [163].The following protocol preceded the production stage of MD simulations. All atoms of the complex were first restrained at their crystal structure positions with a force constant of 10 Kcal mol^-1^ Å^-2^. The system was subjected to the following simulation annealing to ensure the proper equilibration. The temperature was increased from 0K to 500K at a rate of 1K per 1ps and was kept at 500K for 500ps. The temperature was then decreased from 500 K to 300K at a rate of 1K per 1ps and was kept at 300K for additional 500ps. An NPT production simulation was then run on each of the equilibrated structures keeping the temperature at 300 K and constant pressure of 1 atm.

To obtain charge parameters for the studied kinase inhibitors, the conformations of Nilotinib, Ponatinib, Bosutinib and Dasatinib were extracted from the respective crystal structures of the ABL complexes and then fully optimized at RHF/6-31++ level using the Gaussian 03 package [164]. The initial charge parameters for ligand atoms were obtained using the restrained electrostatic charge fitting procedure [165] from the electrostatic potential of RHF/6-31++ single point calculations on the optimized ligand structures. To ensure consistency of the ligand parameters with the CHARMM force field methodology, we also employed a VMD (Visual Molecular Dynamics) [166] plugin, ffTK (http://www.ks.uiuc.edu/Research/vmd/plugins/fftk/) that facilitates generation of CHARMM-compatible parameters for small molecules [167]. In this protocol and according to best CHARMM force field practices, partial atomic charges were optimized from water-interaction profiles to reproduce QM interactions with a TIP3P water molecule [168]. The adopted procedure follows an original approach for derivation of small molecule parameters in the CHARMM General Force Field (CGENFF) that covers a range of chemical groups in drug molecules, including heterocyclic scaffolds present in the studied ligands [169–172]. The bond lengths, bond angles, dihedral and van der Waals parameters along with the associated force constants were also consistent with a detailed force field parameterization of Imatinib [76].

PCA of the MD conformational ensembles was based on the set of backbone heavy atoms (N, Cα, Cβ, C, O) and on the Cα atoms only to determine the essential dynamics of the protein systems. The calculations were performed using the CARMA package [173].The frames are saved every 5 ps, and a total of 10,000 frames were used to compute the correlation matrices for each simulation. For comparison, we also employed the elastic network model (ENM) and computed ENM-based lowest normal modes using the Anisotropic Network Model web server [174].

### Binding Free Energy Calculations

The binding free energy if inhibitor-kinase bindin*g* was calculated using MM-GBSA approach [87–89]. In this approach the binding free energy Δ*G*_*bind*_ is written as the sum of the gas phase contribution Δ*G*_*MM*_, the solvation free energy Δ*G*_*solv*_, and an entropic contribution –*T*Δ*S*

$$\Delta G_{bind}=<\Delta G_{MM}>+<\Delta G_{solv}>-<T\Delta S>$$ (1)

The brackets <> denote an average of these contributions calculated over the MD trajectories. The gas-phase contribution < Δ*G*_*MM*_ > to the binding free energy is the difference in the molecular mechanics energy of the complex and the isolated protein and ligand. This contribution is the sum of the differences in the internal energies Δ*E*_intra_, the van der Waals interaction energy Δ*E*_*vdw*_, and the electrostatic interaction energy Δ*E*_*elec*_:

$$<\Delta G_{MM}>=\Delta E_{\text{intra}}+\Delta E_{vdw}+\Delta E_{elec}$$ (2)

$$E_{\text{intra}}=E_{bond}+E_{vdw}+E_{elec}$$ (3)

where *E*_*bond*_ is the energy of the bonded terms (bonds, angles, dihedral angles, and improper angles) of a given molecule; *E*_*vdw*_ is the van der Waals energy of the molecule; and *E*_*elec*_ is the electrostatic energy of the molecule. These contributions are calculated according to the CHARMM22 molecular mechanics force field.

The solvation free energy Δ*G*_*solv*_ is the difference between the solvation energy of the complex and solvation free energies of the isolated protein and ligand:

$$\Delta G_{solv}=\Delta G_{solv}^{complex}-\Delta G_{solv}^{protein}-\Delta G_{solv}^{ligand}$$ (4)

$$\Delta G_{solv}=\Delta G_{solv}^{np}+\Delta G_{solv}^{elec}$$ (5)

The solvation free energy of a molecule is given as the sum of nonpolar and polar contributions. The nonpolar contribution is computed using the solvent accessible surface are (SASA) model and give as $\Delta G_{solv}^{np}=\sigma *SASA$ where the parameter *σ* = 0.0072 kcal/ (mol*Å^2^). The electrostatic contribution to the solvation free energy $\Delta G_{solv}^{elec}$ was calculated using the analytical generalized Born (GB) model implemented in CHARMM. This model is known to accurately reproduce the solvation free energies calculated by solving the Poisson equations. All energy terms were calculated for 10,000 frames regularly separated by 20 ps along the 200ns trajectory performed for the complex.

The entropy contribution consists of translational Δ*S*_*trans*_, rotational Δ*S*_*rot*_ and vibrational Δ*S*_*vib*_ components:

$$\Delta S=\Delta S_{trans}+\Delta S_{rot}+\Delta S_{vib}$$ (6)

The vibrational entropy terms were computed using normal mode analysis that yields better convergence than the quasiharmomic analysis from MD trajectories. The VIBRAN module of the CHARMM program was used to calculate and diagonalize the force constant matrix for the normal mode vectors and frequencies determination. The normal modes were calculated on minimized average structures obtained from MD simulations. The minimization was performed using the Newton–Raphson minimization algorithm, using the same cutoff scheme and constraints as for the normal mode calculations. Since the normal mode analysis is computationally demanding, −*T*Δ*S* was averaged over only 500 frames extracted from the 200 ns MD trajectories. All energy terms are calculated using single 200 ns trajectories of the kinase-inhibitor complexes. This is followed by separation of the complexes into isolated protein and ligand structures and subsequent minimization of the isolated molecules without conducting additional simulations of the individual protein. With this simplified approach, the difference in the internal energy of the isolated molecules upon complexation is neglected and generally yields better convergence of binding free energies.

### Computational Alanine Scanning

Computational alanine scanning was performed by replacing the side chain of a given residue by an alanine and recalculating the absolute binding free energy for the mutated system [85, 86]. The difference in the binding free energy of the wild type and alanine mutant ΔΔ*G*_*bind*_ may be evaluated as follows:

$$\Delta \Delta G_{bind}=\Delta G_{bind}^{mut}-\Delta G_{bind}^{WT}$$ (7)

$$<\Delta \Delta G_{bind}>=<\Delta E_{vdw}>+<\Delta E_{elec}>+<\Delta \Delta G_{solv}^{np}>+<\Delta \Delta G_{solv}^{elec}>-T<\Delta \Delta S>$$ (8)

The binding free energy of the alanine mutant is calculated using the MM-GBSA approach using the snapshots obtained from MD simulations of the WT complex. The side chain of the residue under investigation is truncated, replacing the Cγ atom by hydrogen. All energy terms were calculated for 10,000 snapshots along the 200ns trajectory performed for the WT complex. For each of these snapshots of the WT complex, the mutated side chain was minimized under the fixed position of the remaining system using 1000 steps of steepest decent and Newton–Raphson minimization before calculating the energy terms. A central assumption of computational alanine scanning approach is that mutations would introduce only local structural perturbations of the system that are sufficiently moderate that the effect on the binding free energy may be gleaned from MD simulations of the WT system. Due to computational cost, the contribution of the entropy term to the binding free energies upon alanine mutations was neglected. Although it is possible to run separate MD trajectories on both WT and mutant complexes, these simulations should be more warranted when the crystal structure of the alanine mutant is available [175]. In this case, the absolute Δ*G* values can be calculated for each complex by averaging the results over separate MD trajectories and their difference can be effectively considered as ΔΔ*G*_*bind*_. These simulations performed for WT and mutant complexes should represent global structural changes and describe a more rigorous effect of a give residue on binding affinity. Given a significant number of studied complexes and an attempt to estimate local energetic changes and determine energetic hot spots, we opted out for the former approach of using only MD simulations of the WT complexes.

### The Residue Interaction Networks and Topological Parameters

A graph-based representation of proteins was used in the protein structure network analysis, where amino acid residues were considered as nodes and edges correspond to the nonbonding residue-residue interactions. The pair of residues with the interaction strength *I*_*ij*_ greater than a user-defined cut-off (*I*_min_) are connected by edges and produce a protein structure network graph for a given interaction strength *I*_min_. The strength of interaction between two amino acid side chains is evaluated as follows:

$$I_{ij}=\frac{n_{ij}}{\sqrt{\left(N_{i}\times N_{j}\right)}}\times 100$$ (9)

where *n*_*ij*_ is number of distinct atom pairs between the side chains of amino acid residues *i* and *j* that lie within a distance of 4.5 Å. *N*_*i*_ and *N*_*j*_ are the normalization factors for residues *i* and *j* respectively [176,177]. The number of interaction pairs including main-chain and side-chain made by residue type *i* with all its surrounding residues in a protein *k* is evaluated. Similar to the arguments presented in our earlier studies [157], we considered any pair of residues to be connected if *I*_min_ was greater than 3.0%. We treat protein–ligand complexes as interaction networks in which the nodes of the network are formed by both amino acid residues and ligand atoms. In the network model, the binding of a ligand introduces new edges in the protein network. A weighted network representation of the protein structure is adopted that includes non-covalent connectivity of side chains and residue cross-correlation fluctuation matrix [178]. In this model of a protein network, the weight *w*_*ij*_ of an edge between nodes *i* and *j* is measured as *w*_*ij*_ = − log(|*C*_*ij*_|) where *C*_*ij*_ is the element of the covariance matrix measuring the cross-correlation between fluctuations of residues is *i* and *j* obtained from MD simulations. The shortest paths between two residues are determined using the Floyd–Warshall algorithm [179]. Network calculations were performed using the python module NetworkX (http://networkx.github.io/).

Using the constructed protein structure networks, we computed the residue-based betweenness parameter. The betweenness of residue *i* is defined to be the sum of the fraction of shortest paths between all pairs of residues that pass through residue *i*:

$$C_{b}(n_{i})=\sum_{j<k}^{N}\frac{g_{jk}(i)}{g_{jk}}$$ (10)

where *g*_*jk*_ denotes the number of shortest geodesics paths connecting *j* and *k*, and *g*_*jk*_ (*i*) is the number of shortest paths between residues *j* and *k* passing through the node *n*_*i*_. Residues with high occurrence in the shortest paths connecting all residue pairs have a higher betweenness values. For each node *n*, *the* betweenness value is normalized by the number of node pairs excluding *n* given as (*N* – 1)(*N* – 2) / 2, where *N* is the total number of nodes in the connected component that node *n* belongs to. The normalized betweenness of residue *i* can be expressed as follows:

$$C_{b}(n_{i})=\frac{1}{\left(N-1)(N-2\right)}\sum_{\begin{array}{l} j<k\\ j\neq i\neq k \end{array}}^{N}\frac{g_{jk}(i)}{g_{jk}}$$ (11)

*g*_*jk*_ is the number of shortest paths between residues *j* and k; *g*_*jk*_ (*i*) is the fraction of these shortest paths that pass through residue *i*.

### Mutual Information Networks of Coevolving Protein Residues

Analysis of coevolving residues was carried out using mutual information (MI) between two positions in the multiple sequence alignment (MSA), which reflects the extent to which knowing the amino acid at one position can predict the amino acid identity at the other position. MI was calculated between pairs of columns in the MSA using MISTIC approach and web server [130]. MI is a nonlinear statistic that measures the information between two random and discrete variables. Given that any two positions in the MSA can be considered random variables *x* and *y*, the MI between is these two positions is given by the relationship:

$$MI(i,j)=\sum_{a,b}P(a_{i},b_{j})*\text{log}(\frac{P(a_{i},b_{j})}{P(a_{i})*P(b_{j})})$$ (12)

where *P*(*a*_*i*_, *b*_*j*)_ is the frequency of amino acid *a* occurring at position *i* and amino acid *b* at position *j* of the same sequence. *P*(*a*_*i*_) is the frequency of amino acid *a* occurring at position *i* and *P*(*b*_*j*_) is the frequency of amino acid *b* at position *j*. The amino acid frequency pair *P*(*a*_*i*_, *b*_*j*)_ is calculated as *N*(*a*_*i*_, *b*_*j*_) / *N* where *N*(*a*_*i*_, *b*_*j*_) is the number of times that an amino acid pair (*a*_*i*_, *b*_*j*_) is observed at positions *i* and *j* respectively. *N* is the total number of sequences in the MSA.

MI networks were defined as graphs where nodes were defined as positions in the MSA of the protein tyrosine kinase family and edges were defined between any pair of nodes of MI. MSA were obtained from Pfam [180–182], a database of protein families that includes their annotations and MSA information generated using hidden Markov models. The human tyrosine-protein kinase ABL1 sequence (UniProtKB accession id P00519) matches Pfam-A entry Pkinase_Tyr (PF007714). The seed alignment of the Pkinase_Tyr family (145 sequences) was used as the curated alignment which contains a set of representative sequences of the tyrosine kinase family, from which a profile hidden Markov model (HMM) is generated using the HMMER3 program (http://hmmer.janelia.org/). Each profile is then searched against a primary sequence database based on UniProtKB [183,184] to create the full MSA profile (24806 sequences). The criteria parameters for generating a MSA are the E-value and the number of columns in the multiple-sequence alignment for which sufficient sequences can be found to infer evolutionary couplings. Based on these criteria, for sequences in the dataset, an E-value of 10^−2^ and a column-inclusion threshold of 80% were used in the MSA generation. All sequences in the full MSA score above curated thresholds are included in the full alignments for that family. A statistically significant and diverse number of sequences along with a high quality of the MSA in the Pfam database is an important prerequisite for the calculation of MI. In the MISTIC approach, MSAs with more than 400 sequences and less than 62% identity typically yield good predictive performance values [105].

In the colevolutionary network analysis, we explored the following parameters: the Kullback-Leibler conservation score, the number of coevolving residue interactions per residue, a cumulative mutual information score (cMI), and the proximity mutual information score (pMI). The Kullback-Leibler (KL) divergence score *KLConsScore* is used for measuring sequence conservation in protein tyrosine kinase sequences. For each column of the MSA, the KL conservation is calculated according to the following formula:

$$KLConsScore_{i}=\sum_{i=1}^{N}\text{ln}\frac{P(i)}{Q(i)}$$ (13)

Here, *P*(*i*) is the frequency of amino acid *i* in that position and *Q*(*i*) is the background frequency of the amino acid in nature calculated using an amino acids background frequency distribution obtained from the UniProtKB database [183,184]. CMI is a mutual information score per residue position that characterizes the extent of mutual information in its physical neighborhood. This sequence-based parameter defines an extent to which a given amino acid residue contributes to a mutual information network. CMI is calculated as the sum of MI values above a certain threshold (MI > 6.5) for every amino acid pair in which a particular residue of interest appears. The MI threshold of 6.5 was shown to be an adequate and reliable lower boundary of mutual information interactions [120,121,130].

$$cMI_{x}=\sum_{y,MI(x,y)>t}MI_{\left(x,y\right)}$$ (14)

pMI score for each position is defined as the average of cMI scores of all the residues within a certain physical distance from a given residue when mapped on the protein kinase structure. The distance between each pair of residues in the structure was calculated as the shortest distance between any two atoms, other than hydrogen atoms, that belong to each of the two positions. The threshold distance of 5 Å defines structural proximity of each residue in defining pMI score [130]:

$$pMI_{x}=\frac{1}{N}\sum_{d(x,y),\text{t}}cMI_{\left(x,y\right)}$$ (15)

A sequential circular representation of the MI network implemented in the MISTIC approach and is provided by the web server [130]. The labels in the first (outer) circle correspond to the alignment position and the amino acid code of the reference sequence. The colored square boxes of the second circle indicate the MSA position conservation (highly conserved positions are in red, while less conserved ones are in blue).The third and fourth circles show the pMI and cMI scores as histograms, facing inwards and outwards respectively. The connected lines in the center of the circle link pairs of positions with MI > 6.5.

## Supporting Information

S1 Fig Conformational Dynamics of Dasatinib-Kinase Complexes. Conformational dynamics profiles are shown for the crystal structures of Dasatinib complexes with EPHA4 (A); LYN (B), P38 (C), BMX (D), BTK (E,F). An active DFG-in conformation is adopted in Dasatinib complexes with EPHA4 (pdb id 2Y6O), LYN (pdb id 2ZVA), P38 (pdb id 3LFA), and BTK kinases (pdb id 3K54). Dasatinib binds with the inactive structures of BMX (pdb id 3SXR) and BTK kinases (pdb id 3OCT). In the complex with an inactive BTK conformation (pdb id 3OCT) an intermediate DFG position is adopted which is between the fully DFG-in and DFG-out conformations. Conformational dynamics profiles were computed by projecting MD trajectories onto the essential space of the three lowest frequency modes. The color gradient from blue to red indicates the decreasing structural rigidity (or increasing conformational mobility) of the protein residues and refers to an average value over the backbone atoms in each residue. The R-spine residues are annotated in spheres and colored according to their degree of structural stability. Dasatinib binding modes are shown in sticks and atom-based color-coded. The Pymol program was used for visualization of the protein kinase structures (The PyMOL Molecular Graphics System, Version 1.2r3pre, Schrödinger, and LLC).
(TIF)

S2 Fig MD Simulations of the Crystal Structures of Dasatinib-Kinase Complexes: Equilibrium Fluctuations of Protein Kinase Residues. The computed B-factors obtained from MD simulations of Dasatinib complexes with EPHA4 (A); LYN (B), P38 (C), BMX (D), BTK (E,F). The fluctuations of the inhibitor-interacting residues are highlighted (green diamonds) indicating stability of the binding site residues in simulations.
(TIF)

S3 Fig Structural Alignment of the Binding Site Residues in Dasatinib-Kinase Complexes. Structural alignment of the ensemble-average positions for the binding site residues are shown for Dasatinib complexes with ABL, SRC, EPHA4, LYN, P38, BMX, and BTK kinases. The annotated residues correspond to the ABL residue numbering in the Dasatinib-ABL complex (pdb id 2GQG). The front view (A) and top view (B) show structural stability of these residues (most notably T315, F317, M318, and L370) across all complexes and indicate some positional variability of the DFG motif.
(TIF)

S4 Fig The Ensemble-Average Conformations of Dasatinib and Functional Kinase Regions: MD simulations of Dasatinib-Kinase Complexes. The ensemble-average binding mode of Dasatinib and functional regions (αC-helix, R-spine, K-E catalytic pair) from MD simulations of Dasatinib complexes with EPHA4 (A); LYN (B), P38 (C), BMX (D), BTK (E,F). In Dasatinib complexes with the DFG-out inactive conformations of BMX (D) and BTK (E), the R-spine is partially disassembled, the αC-helix moved away from the catalytically competent position, and the characteristic salt bridge K-E is broken. The binding mode of Dasatinib remained stable in MD simulations of the crystal structures in all Dasatinib-kinase complexes. The R-spine residues are annotated in spheres, the αC-helix is shown in ribbons, and the binding mode of Dasatinib is shown in sticks. The residues forming a catalytic K-E salt bridge in the active site are shown in sticks.
(TIF)

S5 Fig The Experimental Inhibition Constants of Kinase Inhibitors against ABL Kinase. The experimental IC50 values of kinase inhibitors (Imatinib, Nilotinib, Ponatinib, Dasatinib, Bosutinib) against ABL kinase are collected from biochemical and clinical studies [67–69]. These experimental studies confirmed a range of mutations (shown in different colors) where changes in the inhibition constants correlate with the degree of drug resistance. The logarithmic scale for IC50 values is employed.
(TIF)

S6 Fig The Network Bridging Inhibitor Effect on Residue Centrality Distributions in Dasatinib-Kinase Complexes. The residue-based betweenness distributions of Dasatinib complexes with EPHA4 (A), LYN (B), P38 (C), BMX (D), BTK (E,F). The distributions are obtained by averaging the results of MD simulations. The overall distributions are shown in blue and the distribution corresponding to the inhibitor-interacting residues is shown in green.
(TIF)

## References

[1] Huse M, Kuriyan J. The conformational plasticity of protein kinases. Cell. 2002;109: 275–282. 12015977 10.1016/s0092-8674(02)00741-9

[2] Nolen B, Taylor S, Ghosh G. Regulation of protein kinases; controlling activity through activation segment conformation. Mol Cell. 2004;15: 661–675. 15350212 10.1016/j.molcel.2004.08.024

[3] Taylor SS, Kornev AP. Protein kinases: evolution of dynamic regulatory proteins. Trends Biochem Sci. 2011;36:65–77. 10.1016/j.tibs.2010.09.006 20971646 PMC3084033

[4] Endicott JA, Noble ME, Johnson LN. The structural basis for control of eukaryotic protein kinases. Annu Rev Biochem. 2012;81: 587–613. 10.1146/annurev-biochem-052410-090317 22482904

[5] Taylor SS, Keshwani MM, Steichen JM, Kornev AP. Evolution of the eukaryotic protein kinases as dynamic molecular switches. Philos Trans R Soc Lond B Biol Sci. 2012;367: 2517–2528. 10.1098/rstb.2012.0054 22889904 PMC3415842

[6] Taylor SS, Ilouz R, Zhang P, Kornev AP. Assembly of allosteric macromolecular switches: lessons from PKA. Nat Rev Mol Cell Biol. 2012;13: 646–658. 10.1038/nrm3432 22992589 PMC3985763

[7] Artim SC, Mendrola JM, Lemmon MA. Assessing the range of kinase autoinhibition mechanisms in the insulin receptor family. Biochem J. 2012;448: 213–220. 10.1042/BJ20121365 22992069 PMC3492919

[8] Oruganty K, Kannan N. Design principles underpinning the regulatory diversity of protein kinases. Philos Trans R Soc Lond B Biol Sci. 2012;367: 2529–2539. 10.1098/rstb.2012.0015 22889905 PMC3415841

[9] Taylor SS, Zhang P, Steichen JM, Keshwani MM, Kornev AP. PKA: lessons learned after twenty years. Biochim Biophys Acta, 2013;1834:1271–1278. 10.1016/j.bbapap.2013.03.007 23535202 PMC3763834

[10] Meharena HS, Chang P, Keshwani MM, Oruganty K, Nene AK, Kannan N, et al Deciphering the structural basis of eukaryotic protein kinase regulation. PLoS Biol. 2013;11:e1001680 10.1371/journal.pbio.1001680 24143133 PMC3797032

[11] Lemmon MA, Schlessinger J. Cell signaling by receptor tyrosine kinases. Cell. 2010;141:1117–11134. 10.1016/j.cell.2010.06.011 20602996 PMC2914105

[12] Roskoski R Jr. The ErbB/HER family of protein-tyrosine kinases and cancer. Pharmacol Res. 2014;79:34–74. 10.1016/j.phrs.2013.11.002 24269963

[13] Noble ME, Endicott JA, Johnson LN. Protein kinase inhibitors: insights into drug design from structure. Science. 2004;303:1800–1805. 15031492 10.1126/science.1095920

[14] Zhang J, Yang PL, Gray NS. Targeting cancer with small molecule kinase inhibitors. Nat Rev Cancer. 2009; 9: 28–39. 10.1038/nrc2559 19104514 PMC12406740

[15] Dar AC, Shokat KM. The evolution of protein kinase inhibitors from antagonists to agonists of cellular signaling. Annu Rev Biochem. 2011;80: 769–795. 10.1146/annurev-biochem-090308-173656 21548788

[16] Hantschel O, Grebien F, Superti-Furga G. The growing arsenal of ATP-competitive and allosteric inhibitors of Bcr-Abl. Cancer Res. 2012;72:4890–4895. 10.1158/0008-5472.CAN-12-1276 23002203 PMC3517953

[17] Cowan-Jacob SW, Jahnke W, Knapp S. Novel approaches for targeting kinases: allosteric inhibition, allosteric activation and pseudokinases. Future Med Chem. 2014;6: 541–561. 10.4155/fmc.13.216 24649957

[18] Schindler T, Bornmann W, Pellicena P, Miller WT, Clarkson B, Kuriyan J. Structural mechanism for STI-571 inhibition of Abelson tyrosine kinase. Science. 2000;289: 1938–1942. 10988075 10.1126/science.289.5486.1938

[19] Nagar B, Bornmann WG, Pellicena P, Schindler T, Veach DR, Miller WT, et al Crystal structures of the kinase domain of c- Abl in complex with the small molecule inhibitors PD173955 and Imatinib (STI-571). Cancer Res. 2002;62: 4236–4243. 12154025

[20] Nagar B, Hantschel O, Young MA, Scheffzek K, Veach DR, Bornmann W, et al Structural basis for the autoinhibition of c-Abl tyrosine kinase. Cell. 2003;112:859–871. 12654251 10.1016/s0092-8674(03)00194-6

[21] Levinson NM, Kuchment O, Shen K, Young MA, Koldobskiy M, Karplus M, et al A Src-like inactive conformation in the Abl tyrosine kinase domain. PLoS Biol. 2006;4: e144 16640460 10.1371/journal.pbio.0040144 PMC1450098

[22] Tokarski JS, Newitt JA, Chang CY, Cheng JD, Wittekind M, Kiefer SE, et al The structure of Dasatinib (BMS-354825) bound to activated ABL kinase domain elucidates its inhibitory activity against Imatinib-resistant ABL mutants. Cancer Res. 2006; 66: 5790–5797. 16740718 10.1158/0008-5472.CAN-05-4187

[23] Seeliger MA, Nagar B, Frank F, Cao X, Henderson MN, Kuriyan J. C-Src binds to the cancer drug imatinib with an inactive Abl/c-Kit conformation and a distributed thermodynamic penalty. Structure. 2007; 15:299–311. 17355866 10.1016/j.str.2007.01.015

[24] Seeliger MA, Ranjitkar P, Kasap C, Shan Y, Shaw DE, Shah NP, et al Equally potent inhibition of c-Src and Abl by compounds that recognize inactive kinase conformations. Cancer Res. 2009;69: 2384–2392. 10.1158/0008-5472.CAN-08-3953 19276351 PMC2678021

[25] Kornev AP, Taylor SS, Ten Eyck LF. A helix scaffold for the assembly of active protein kinases. Proc Natl Acad Sci U S A. 2008;105: 14377–14382. 10.1073/pnas.0807988105 18787129 PMC2533684

[26] Ten Eyck LF, Taylor SS, Kornev AP. Conserved spatial patterns across the protein kinase family. Biochim Biophys Acta. 2008;1784: 238–243. 18067871 10.1016/j.bbapap.2007.11.002

[27] Hantschel O. Structure, regulation, signaling, and targeting of Abl kinases in cancer. Genes Cancer. 2012;3: 436–446. 10.1177/1947601912458584 23226581 PMC3513796

[28] Panjarian S, Iacob RE, Chen S, Engen JR, Smithgall TE. Structure and dynamic regulation of Abl kinases. J Biol Chem. 2013;288: 5443–5450. 10.1074/jbc.R112.438382 23316053 PMC3581414

[29] Reddy EP, Aggarwal AK. The ins and outs of Bcr-Abl inhibition. Genes Cancer. 2012; 3:447–454. 10.1177/1947601912462126 23226582 PMC3513788

[30] Lamontanara AJ, Gencer EB, Kuzyk O, Hantschel O. Mechanisms of resistance to Bcr-Abl and other kinase inhibitors. Biochim Biophys Acta. 2013; 1834:1449–1459. 10.1016/j.bbapap.2012.12.009 23277196

[31] Weisberg E, Manley PW, Breitenstein W, Brüggen J, Cowan-Jacob SW, Ray A, et al Characterization of AMN107, a selective inhibitor of native and mutant Bcr-Abl. Cancer Cell. 2005; 7: 129–141. 15710326 10.1016/j.ccr.2005.01.007

[32] O'Hare T, Walters DK, Stoffregen EP, Jia T, Manley PW, Mestan J, et al In vitro activity of Bcr-Abl inhibitors AMN107 and BMS-354825 against clinically relevant imatinib resistant Abl kinase domain mutants. Cancer Res. 2005; 65: 4500–4505. 15930265 10.1158/0008-5472.CAN-05-0259

[33] O'Hare T, Shakespeare WC, Zhu X, Eide CA, Rivera VM, Wang F, et al AP24534, a pan-BCR-ABL inhibitor for chronic myeloid leukemia, potently inhibits the T315I mutant and overcomes mutation-based resistance. Cancer Cell. 2009;16: 401–412. 10.1016/j.ccr.2009.09.028 19878872 PMC2804470

[34] Zhou T, Commodore L, Huang WS, Wang Y, Thomas M, Keats J, et al Structural mechanism of the Pan-BCR-ABL inhibitor ponatinib (AP24534): lessons for overcoming kinase inhibitor resistance. Chem Biol Drug Des. 2011;77: 1–11. 10.1111/j.1747-0285.2010.01054.x 21118377

[35] Carter TA, Wodicka LM, Shah NP, Velasco AM, Fabian MA, Treiber DK, et al Inhibition of drug-resistant mutants of ABL, KIT, and EGF receptor kinases. Proc Natl Acad Sci U S A. 2005;102: 11011–11016. 16046538 10.1073/pnas.0504952102 PMC1180625

[36] Fabian MA, Biggs WH 3rd, Treiber DK, Atteridge CE, Azimioara MD, Benedetti MG, et al A small molecule-kinase interaction map for clinical kinase inhibitors. Nat Biotechnol. 2005;23: 329–336. 15711537 10.1038/nbt1068

[37] Bantscheff M, Eberhard D, Abraham Y, Bastuck S, Boesche M, Hobson S, et al Quantitative chemical proteomics reveals mechanisms of action of clinical ABL kinase inhibitors. Nat Biotechnol. 2007;25: 1035–1044. 17721511 10.1038/nbt1328

[38] Rix U, Hantschel O, Dürnberger G, Remsing Rix LL, Planyavsky M, Fernbach NV, et al Chemical proteomic profiles of the BCR-ABL inhibitors imatinib, nilotinib, and dasatinib reveal novel kinase and nonkinase targets. Blood. 2007;110: 4055–4063. 17720881 10.1182/blood-2007-07-102061

[39] Hantschel O, Rix U, Schmidt U, Bürckstümmer T, Kneidinger M, Schütze G, et al The Btk tyrosine kinase is a major target of the Bcr-Abl inhibitor dasatinib. Proc Natl Acad Sci U S A. 2007;104: 13283–13288. 17684099 10.1073/pnas.0702654104 PMC1940229

[40] Getlik M, Grütter C, Simard JR, Klüter S, Rabiller M, Rode HB, et al Hybrid compound design to overcome the gatekeeper T338M mutation in c-Src. J Med Chem. 2009;52: 3915–3926. 10.1021/jm9002928 19462975

[41] Williams NK, Lucet IS, Klinken SP, Ingley E, Rossjohn J. Crystal structures of the Lyn protein tyrosine kinase domain in its apo- and inhibitor-bound state. J Biol Chem. 2009;284:284–291. 10.1074/jbc.M807850200 18984583

[42] Farenc C, Celie PH, Tensen CP, de Esch IJ, Siegal G. Crystal structure of the EphA4 protein tyrosine kinase domain in the apo- and dasatinib-bound state. FEBS Lett. 2011;585: 3593–3599. 10.1016/j.febslet.2011.10.028 22036717

[43] Muckelbauer J, Sack JS, Ahmed N, Burke J, Chang CY, Gao M, et al X-ray crystal structure of bone marrow kinase in the x chromosome: a Tec family kinase. Chem Biol Drug Des. 2011;78: 739–748. 10.1111/j.1747-0285.2011.01230.x 21883956

[44] Marcotte DJ, Liu YT, Arduini RM, Hession CA, Miatkowski K, Wildes CP, et al Structures of human Bruton's tyrosine kinase in active and inactive conformations suggest a mechanism of activation for TEC family kinases. Protein Sci. 2010;19: 429–439. 10.1002/pro.321 20052711 PMC2866269

[45] Vajpai N, Strauss A, Fendrich G, Cowan-Jacob SW, Manley PW, Grzesiek S, et al Solution conformations and dynamics of ABL kinase-inhibitor complexes determined by NMR substantiate the different binding modes of imatinib/nilotinib and dasatinib. J Biol Chem. 2008;283: 18292–18302. 10.1074/jbc.M801337200 18434310

[46] Wodicka LM, Ciceri P, Davis MI, Hunt JP, Floyd M, Salerno S, et al Activation state-dependent binding of small molecule kinase inhibitors: structural insights from biochemistry. Chem Biol. 2010;17: 1241–1249. 10.1016/j.chembiol.2010.09.010 21095574

[47] Puttini M, Coluccia AM, Boschelli F, Cleris L, Marchesi E, Donella-Deana A, et al In vitro and in vivo activity of SKI-606, a novel Src-Abl inhibitor, against imatinib-resistant Bcr-Abl+ neoplastic cells. Cancer Res. 2006;66: 11314–11322. 17114238 10.1158/0008-5472.CAN-06-1199

[48] Levinson NM, Boxer SG. Structural and spectroscopic analysis of the kinase inhibitor bosutinib and an isomer of bosutinib binding to the Abl tyrosine kinase domain. PLoS One. 2012;7: e29828 10.1371/journal.pone.0029828 22493660 PMC3320885

[49] Levinson NM, Boxer SG. A conserved water-mediated hydrogen bond network defines bosutinib's kinase selectivity. Nat Chem Biol. 2014;10: 127–132. 10.1038/nchembio.1404 24292070 PMC3947016

[50] Karaman MW, Herrgard S, Treiber DK, Gallant P, Atteridge CE, Campbell BT, et al A quantitative analysis of kinase inhibitor selectivity. Nat Biotechnol. 2008; 26: 127–132. 10.1038/nbt1358 18183025

[51] Davis MI, Hunt JP, Herrgard S, Ciceri P, Wodicka LM, Pallares G, et al Comprehensive analysis of kinase inhibitor selectivity. Nat Biotechnol. 2011;29: 1046–1051. 10.1038/nbt.1990 22037378

[52] Anastassiadis T, Deacon SW, Devarajan K, Ma H, Peterson JR. Comprehensive assay of kinase catalytic activity reveals features of kinase inhibitor selectivity. Nat Biotechnol. 2011;29: 1039–1045. 10.1038/nbt.2017 22037377 PMC3230241

[53] Giansanti P, Preisinger C, Huber KV, Gridling M, Superti-Furga G, Bennett KL, et al Evaluating the promiscuous nature of tyrosine kinase inhibitors assessed in A431 epidermoid carcinoma cells by both chemical- and phosphoproteomics. ACS Chem Biol. 2014;9: 1490–1498. 10.1021/cb500116c 24804581

[54] Hari SB, Perera BG, Ranjitkar P, Seeliger MA, Maly DJ. Conformation-selective inhibitors reveal differences in the activation and phosphate-binding loops of the tyrosine kinases Abl and Src. ACS Chem Biol. 2013;8: 2734–2743. 10.1021/cb400663k 24106839 PMC3880807

[55] Zhao Z, Wu H, Wang L, Liu Y, Knapp S, Liu Q, et al Exploration of type II binding mode: A privileged approach for kinase inhibitor focused drug discovery? ACS Chem Biol. 2014; 9: 1230–1241. 10.1021/cb500129t 24730530 PMC4068218

[56] Redaelli S, Piazza R, Rostagno R, Magistroni V, Perini P, Marega M, et al Activity of bosutinib, dasatinib, and nilotinib against 18 imatinib-resistant BCR/ABL mutants. J Clin Oncol. 2009;27: 469–471. 10.1200/JCO.2008.19.8853 19075254

[57] Cortes J, Jabbour E, Kantarjian H, Yin CC, Shan J, O'Brien S, et al Dynamics of BCR-ABL kinase domain mutations in chronic myeloid leukemia after sequential treatment with multiple tyrosine kinase inhibitors. Blood. 2007;110: 4005–4011. 17785585 10.1182/blood-2007-03-080838

[58] Jabbour E, Jones D, Kantarjian HM, O'Brien S, Tam C, Koller C, et al Long-term outcome of patients with chronic myeloid leukemia treated with second-generation tyrosine kinase inhibitors after imatinib failure is predicted by the in vitro sensitivity of BCR-ABL kinase domain mutations. Blood. 2009;114: 2037–2043. 10.1182/blood-2009-01-197715 19567878 PMC4186638

[59] Hughes T, Saglio G, Branford S, Soverini S, Kim DW, Müller MC, et al Impact of baseline BCR-ABL mutations on response to nilotinib in patients with chronic myeloid leukemia in chronic phase. J Clin Oncol. 2009;27: 4204–4210. 10.1200/JCO.2009.21.8230 19652056 PMC4979230

[60] Müller MC, Cortes JE, Kim DW, Druker BJ, Erben P, Pasquini R, et al Dasatinib treatment of chronic phase chronic myeloid leukemia: analysis of responses according to pre-existing BCR-ABL mutations. Blood. 2009;114: 4944–4953. 10.1182/blood-2009-04-214221 19779040 PMC4916940

[61] Branford S, Melo JV, Hughes TP. Selecting optimal second-line tyrosine kinase inhibitor therapy for chronic myeloid leukemia patients after imatinib failure: does the BCR-ABL mutation status really matter? Blood. 2009;114: 5426–5435. 10.1182/blood-2009-08-215939 19880502

[62] O'Hare T, Pollock R, Stoffregen EP, Keats JA, Abdullah OM, Moseson EM, et al Inhibition of wild-type and mutant Bcr-Abl by AP23464, a potent ATP-based oncogenic protein kinase inhibitor: implications for CML. Blood. 2004;104: 2532–2539. 15256422 10.1182/blood-2004-05-1851

[63] Cassuto O, Dufies M, Jacquel A, Robert G, Ginet C, Dubois A, et al All tyrosine kinase inhibitor-resistant chronic myelogenous cells are highly sensitive to ponatinib. Oncotarget. 2012;3: 1557–1565. 23238683 10.18632/oncotarget.692 PMC3681494

[64] Redaelli S, Mologni L, Rostagno R, Piazza R, Magistroni V, Ceccon M, et al Three novel patient-derived BCR/ABL mutants show different sensitivity to second and third generation tyrosine kinase inhibitors. Am J Hematol. 2012;87: E125–E128. 10.1002/ajh.23338 23044928

[65] Buffa P, Romano C, Pandini A, Massimino M, Tirrò E, Di Raimondo F, et al BCR-ABL residues interacting with ponatinib are critical to preserve the tumorigenic potential of the oncoprotein. FASEB J. 2014;28: 1221–1236. 10.1096/fj.13-236992 24297701

[66] Zabriskie MS, Eide CA, Tantravahi SK, Vellore NA, Estrada J, Nicolini FE, et al BCR-ABL1 compound mutations combining key kinase domain positions confer clinical resistance to ponatinib in Ph chromosome-positive leukemia. Cancer Cell. 2014;26: 428–442. 10.1016/j.ccr.2014.07.006 25132497 PMC4160372

[67] Soverini S, Rosti G, Iacobucci I, Baccarani M, Martinelli G. Choosing the best second-line tyrosine kinase inhibitor in imatinib-resistant chronic myeloid leukemia patients harboring Bcr-Abl kinase domain mutations: how reliable is the IC_50_? Oncologist. 2011;16: 868–876. 10.1634/theoncologist.2010-0388 21632458 PMC3228229

[68] Soverini S, Hochhaus A, Nicolini FE, Gruber F, Lange T, Saglio G, et al BCR-ABL kinase domain mutation analysis in chronic myeloid leukemia patients treated with tyrosine kinase inhibitors: recommendations from an expert panel on behalf of European LeukemiaNet. Blood. 2011;118: 1208–1215. 10.1182/blood-2010-12-326405 21562040

[69] Soverini S, De Benedittis C, Papayannidis C, Paolini S, Venturi C, Iacobucci I, et al Drug resistance and BCR-ABL kinase domain mutations in Philadelphia chromosome-positive acute lymphoblastic leukemia from the imatinib to the second-generation tyrosine kinase inhibitor era: The main changes are in the type of mutations, but not in the frequency of mutation involvement. Cancer. 2014;120: 1002–1009. 10.1002/cncr.28522 24382642

[70] Pricl S, Fermeglia M, Ferrone M, Tamborini E. T315I-mutated Bcr-Abl in chronic myeloid leukemia and imatinib: insights from a computational study. Mol Cancer Ther. 2005;4: 1167–1174. 16093432 10.1158/1535-7163.MCT-05-0101

[71] Verkhivker GM. In silico profiling of tyrosine kinases binding specificity and drug resistance using Monte Carlo simulations with the ensembles of protein kinase crystal structures. Biopolymers. 2007;85: 333–348. 17167796 10.1002/bip.20656

[72] Lee TS, Potts SJ, Kantarjian H, Cortes J, Giles F, Albitar M. Molecular basis explanation for imatinib resistance of BCR-ABL due to T315I and P-loop mutations from molecular dynamics simulations. Cancer. 2008;112: 1744–1753. 10.1002/cncr.23355 18338744

[73] Shan Y, Seeliger MA, Eastwood MP, Frank F, Xu H, Jensen MØ, et al A conserved protonation-dependent switch controls drug binding in the Abl kinase. Proc Natl Acad Sci U S A. 2009;106: 139–144. 10.1073/pnas.0811223106 19109437 PMC2610013

[74] Dixit A, Verkhivker GM. Hierarchical modeling of activation mechanisms in the ABL and EGFR kinase domains: thermodynamic and mechanistic catalysts of kinase activation by cancer mutations PLoS Comput Biol. 2009;5: e1000487 10.1371/journal.pcbi.1000487 19714203 PMC2722018

[75] Dixit A, Verkhivker GM. Computational modeling of allosteric communication reveals organizing principles of mutation-induced signaling in ABL and EGFR kinases. PLoS Comput Biol. 2011;7: e1002179 10.1371/journal.pcbi.1002179 21998569 PMC3188506

[76] Aleksandrov A, Simonson T. A molecular mechanics model for Imatinib and Imatinib:kinase binding. J Comput Chem. 2010; 31:1550–1560. 10.1002/jcc.21442 20020482

[77] Aleksandrov A, Simonson T. Molecular dynamics simulations show that conformational selection governs the binding preferences of Imatinib for several tyrosine kinases. J Biol Chem. 2010;285: 13807–13815. 10.1074/jbc.M110.109660 20200154 PMC2859544

[78] Lovera S, Sutto L, Boubeva R, Scapozza L, Dölker N, Gervasio FL. The different flexibility of c-Src and c-Abl kinases regulates the accessibility of a druggable inactive conformation. J Am Chem Soc. 2012;134: 2496–2499. 10.1021/ja210751t 22280319

[79] Lin YL, Meng Y, Jiang W, Roux B. Explaining why Gleevec is a specific and potent inhibitor of Abl kinase. Proc Natl Acad Sci U S A. 2013;110: 1664–1669. 10.1073/pnas.1214330110 23319661 PMC3562763

[80] Lin YL, Roux B. Computational analysis of the binding specificity of Gleevec to Abl, c-Kit, Lck, and c-Src tyrosine kinases. J Am Chem Soc. 2013;135: 14741–14753. 10.1021/ja405939x 24001034 PMC4026022

[81] Lin YL, Meng Y, Huang L, Roux B. Computational study of Gleevec and G6G reveals molecular determinants of kinase inhibitor selectivity. J Am Chem Soc. 2014;136: 14753–14762. 10.1021/ja504146x 25243930 PMC4210138

[82] Laurini E, Posocco P, Fermeglia M, Gibbons DL, Quintás-Cardama A, Pricl S. Through the open door: Preferential binding of dasatinib to the active form of BCR-ABL unveiled by in silico experiments. Mol Oncol. 2013;7: 968–975. 10.1016/j.molonc.2013.06.001 23816609 PMC5528448

[83] Gibbons DL, Pricl S, Posocco P, Laurini E, Fermeglia M, Sun H, et al Molecular dynamics reveal BCR-ABL1 polymutants as a unique mechanism of resistance to PAN-BCR-ABL1 kinase inhibitor therapy. Proc Natl Acad Sci U S A. 2014;111: 3550–3555. 10.1073/pnas.1321173111 24550512 PMC3948238

[84] Tanneeru K, Guruprasad L. Ponatinib is a pan-BCR-ABL kinase inhibitor: MD simulations and SIE study. PLoS One. 2013; 8:e78556 10.1371/journal.pone.0078556 24236021 PMC3827254

[85] Massova I, Kollman PA. Computational alanine scanning to probe protein−protein interactions: a novel approach to evaluate binding free energies. J Am Chem Soc. 1999; 121: 8133–8143.

[86] Huo S, Massova I, Kollman PA. Computational alanine scanning of the 1:1 human growth hormone-receptor complex. J Comput Chem. 2002;23: 15–27. 11913381 10.1002/jcc.1153

[87] Wang J, Wolf RM, Caldwell JW, Kollman PA, Case DA. Development and testing of a general amber force field. J Comput Chem. 2004;25: 1157–1174. 15116359 10.1002/jcc.20035

[88] Srinivasan J, Cheatham TE, Cieplak P, Kollman PA, Case DA. Continuum solvent studies of the stability of DNA, RNA and phosphoramidate-DNA helices. J Am Chem Soc 1998;120:9401–9409.

[89] Kollman PA, Massova I, Reyes C, Kuhn B, Huo S, Chong L, et al Calculating structures and free energies of complex molecules: combining molecular mechanics and continuum models. Acc Chem Res. 2000;33: 889–897. 11123888 10.1021/ar000033j

[90] Vendruscolo MN, Dokholyan V, Paci E, Karplus M. Small-world view of the amino acids that play a key role in protein folding. Phys Rev E Stat Nonlin Soft Matter Phys. 2002;65: 061910 12188762 10.1103/PhysRevE.65.061910

[91] Dokholyan NV, Li L, Ding F, Shakhnovich EI. Topological determinants of protein folding. Proc Natl Acad Sci USA. 2002;99: 8637–8641. 12084924 10.1073/pnas.122076099 PMC124342

[92] Greene LH, Higman VA. Uncovering network systems within protein structures. J Mol Biol. 2003;334: 781–791. 14636602 10.1016/j.jmb.2003.08.061

[93] Atilgan AR, Akan P, Baysal C. Small-world communication of residues and significance for protein dynamics. Biophys J. 2004; 86: 85–91. 14695252 10.1016/S0006-3495(04)74086-2 PMC1303839

[94] del Sol A, O'Meara P. Small-world network approach to identify key residues in protein-protein interaction. Proteins. 2005;58: 672–682. 15617065 10.1002/prot.20348

[95] del Sol A, Fujihashi H, O'Meara P. Topology of small-world networks of protein-protein complex structures. Bioinformatics. 2005;21: 1311–1315. 15659419 10.1093/bioinformatics/bti167

[96] Amitai G, Shemesh A, Sitbon E, Shklar M, Netanely D, Venger I, et al Network analysis of protein structures identifies functional residues. J Mol Biol. 2004; 344: 1135–1146. 15544817 10.1016/j.jmb.2004.10.055

[97] Hu Z, Bowen D, Southerland WM, del Sol A, Pan Y, Nussinov R, et al Ligand binding and circular permutation modify residue interaction network in DHFR. PLoS Comput Biol. 2007;3: e117 17571919 10.1371/journal.pcbi.0030117 PMC1892607

[98] del Sol A, Fujihashi H, Amoros D, Nussinov R. Residue centrality, functionally important residues, and active site shape: analysis of enzyme and non-enzyme families. Protein Sci. 2006; 15: 2120–2128. 16882992 10.1110/ps.062249106 PMC2242611

[99] del Sol A, Fujihashi H, Amoros D, Nussinov R. Residues crucial for maintaining short paths in network communication mediate signaling in proteins. Mol Sys Biol. 2006;2: 2006.0019. 10.1038/msb4100063 PMC1681495 16738564

[100] Korber BT, Farber RM, Wolpert DH, Lapedes AS. Covariation of mutations in the V3 loop of human immunodeficiency virus type 1 envelope protein: an information theoretic analysis. Proc Natl Acad Sci U S A. 1993;90: 7176–7180. 8346232 10.1073/pnas.90.15.7176 PMC47099

[101] Lockless SW, Ranganathan R. Evolutionarily conserved pathways of energetic connectivity in protein families. Science. 1999;286: 295–299. 10514373 10.1126/science.286.5438.295

[102] Suel GM, Lockless SW, Wall MA, Ranganathan R. Evolutionarily conserved networks of residues mediate allosteric communication in proteins. Nat Struct Biol. 2003;10: 59–69. 12483203 10.1038/nsb881

[103] Halabi N, Rivoire O, Leibler S, Ranganathan R. Protein sectors: evolutionary units of three-dimensional structure. Cell. 2009;138: 774–786. 10.1016/j.cell.2009.07.038 19703402 PMC3210731

[104] McLaughlin RN, Poelwijk FJ, Raman A, Gosal WS, Ranganathan R. The spatial architecture of protein function and adaptation. Nature. 2012;491: 138–142. 10.1038/nature11500 23041932 PMC3991786

[105] Buslje CM, Santos J, Delfino JM, Nielsen M. Correction for phylogeny, small number of observations and data redundancy improves the identification of coevolving amino acid pairs using mutual information. Bioinformatics. 2009; 25: 1125–1131. 10.1093/bioinformatics/btp135 19276150 PMC2672635

[106] Aguilar D, Oliva B, Marino Buslje C. Mapping the mutual information network of enzymatic families in the protein structure to unveil functional features. PLoS One. 2012;7: e41430 10.1371/journal.pone.0041430 22848494 PMC3405127

[107] de Juan D, Pazos F, Valencia A. Emerging methods in protein co-evolution. Nat Rev Genet. 2013;14: 249–261. 10.1038/nrg3414 23458856

[108] Socolich M, Lockless SW, Russ WP, Lee H, Gardner KH, Ranganathan R. Evolutionary information for specifying a protein fold. Nature. 2005;437: 512–518. 16177782 10.1038/nature03991

[109] Morcos F, Pagnani A, Lunt B, Bertolino A, Marks DS, Sander C, et al Direct-coupling analysis of residue coevolution captures native contacts across many protein families. Proc Natl Acad Sci U S A. 2011;108:E1293–E1301. 10.1073/pnas.1111471108 22106262 PMC3241805

[110] Wang J, Zhao Y, Wang Y, Huang J. Molecular dynamics simulations and statistical coupling analysis reveal functional coevolution network of oncogenic mutations in the CDKN2A-CDK6 complex. FEBS Lett. 2013;587: 136–141. 10.1016/j.febslet.2012.11.001 23178718

[111] Hoffman NG, Schiffer CA, Swanstrom R. Covariation of amino acid positions in HIV-1 protease. Virology. 2003;314: 536–0548. 14554082 10.1016/s0042-6822(03)00484-7

[112] Kowarsch A, Fuchs A, Frishman D, Pagel P. Correlated mutations: a hallmark of phenotypic amino acid substitutions. PLoS Comput Biol. 2010;6: e1000923 10.1371/journal.pcbi.1000923 20862353 PMC2940720

[113] Yeang CH, Haussler D. Detecting coevolution in and among protein domains. PLoS Comput Biol. 2007;3: e211 17983264 10.1371/journal.pcbi.0030211 PMC2098842

[114] Lee BC, Park K, Kim D. Analysis of the residue-residue coevolution network and the functionally important residues in proteins. Proteins. 2008;72:863–872. 10.1002/prot.21972 18275083

[115] Chakrabarti S, Panchenko AR. Coevolution in defining the functional specificity. Proteins. 2009;75: 231–240. 10.1002/prot.22239 18831050 PMC2649964

[116] Chakrabarti S, Panchenko AR. Structural and functional roles of coevolved sites in proteins. PLoS One. 2010;5: e8591 10.1371/journal.pone.0008591 20066038 PMC2797611

[117] Zhao Y, Wang Y, Gao Y, Li G, Huang J. Integrated analysis of residue coevolution and protein structures capture key protein sectors in HIV-1 proteins. PLoS One. 2015;10: e0117506 10.1371/journal.pone.0117506 25671429 PMC4324911

[118] Xu F, Du P, Shen H, Hu H, Wu Q, Xie J, et al Correlated mutation analysis on the catalytic domains of serine/threonine protein kinases. PLoS One. 2009;4: e5913 10.1371/journal.pone.0005913 19526051 PMC2690836

[119] Hsu YH, Traugh JA. Reciprocally coupled residues crucial for protein kinase Pak2 activity calculated by statistical coupling analysis. PLoS One. 2010;5:e 9455 10.1371/journal.pone.0009455 20209159 PMC2830475

[120] Marino Buslje C, Teppa E, Di Doménico T, Delfino JM, Nielsen M. Networks of high mutual information define the structural proximity of catalytic sites: implications for catalytic residue identification. PLoS Comput Biol. 2010;6: e1000978 10.1371/journal.pcbi.1000978 21079665 PMC2973806

[121] Teppa E, Wilkins AD, Nielsen M, Buslje CM. Disentangling evolutionary signals: conservation, specificity determining positions and coevolution. Implication for catalytic residue prediction. BMC Bioinformatics. 2012;13: 235 10.1186/1471-2105-13-235 22978315 PMC3515339

[122] Jeon J, Nam HJ, Choi YS, Yang JS, Hwang J, Kim S. Molecular evolution of protein conformational changes revealed by a network of evolutionarily coupled residues. Mol Biol Evol. 2011;28: 2675–2685. 10.1093/molbev/msr094 21470969

[123] Liu Y, Bahar I. Sequence evolution correlates with structural dynamics. Mol Biol Evol. 2012;29:2253–2263. 10.1093/molbev/mss097 22427707 PMC3424413

[124] Amadei A, Linssen AB, Berendsen HJ. Essential dynamics of proteins. Proteins. 1993; 17: 412–425. 8108382 10.1002/prot.340170408

[125] Iacob RE, Zhang J, Gray NS, Engen JR. Allosteric interactions between the myristate- and ATP-site of the Abl kinase. PLoS One. 2011;6: e15929 10.1371/journal.pone.0015929 21264348 PMC3018526

[126] Sobolev V, Sorokine A, Prilusky J, Abola EE, Edelman M. Automated analysis of interatomic contacts in proteins. Bioinformatics. 1999; 15:327–332. 10320401 10.1093/bioinformatics/15.4.327

[127] Marsh JA, Teichmann SA. Relative solvent accessible surface area predicts protein conformational changes upon binding. Structure. 2011; 19:859–867. 10.1016/j.str.2011.03.010 21645856 PMC3145976

[128] Marsh JA. Buried and accessible surface area control intrinsic protein flexibility. J Mol Biol. 2013;425: 3250–3263. 10.1016/j.jmb.2013.06.019 23811058

[129] Fraczkiewicz R, Braun W. Exact and efficient analytical calculation of the accessible surface areas and their gradients for macromolecules. J Comput Chem. 1998;19: 319–333.

[130] Simonetti FL, Teppa E, Chernomoretz A, Nielsen M, Marino Buslje C. MISTIC: Mutual information server to infer coevolution. Nucleic Acids Res. 2013; 41:W8–W14. 10.1093/nar/gkt427 23716641 PMC3692073

[131] Muratore KE, Seeliger MA, Wang Z, Fomina D, Neiswinger J, Havranek JJ, et al Comparative analysis of mutant tyrosine kinase chemical rescue. Biochemistry. 2009; 48:3378–3386. 10.1021/bi900057g 19260709 PMC2714740

[132] McMurrough TA, Dickson RJ, Thibert SM, Gloor GB, Edgell DR. Control of catalytic efficiency by a coevolving network of catalytic and noncatalytic residues. Proc Natl Acad Sci U S A. 2014;111: E2376–E2383. 10.1073/pnas.1322352111 24912189 PMC4060692

[133] Azam M, Seeliger MA, Gray NS, Kuriyan J, Daley GQ. Activation of tyrosine kinases by mutation of the gatekeeper threonine. Nat Struct Mol Biol. 2008; 15:1109–1118. 10.1038/nsmb.1486 18794843 PMC2575426

[134] Sol AD, Sol, Tsai CJ, Ma B, Nussinov R. The origin of allosteric functional modulation: multiple pre-existing pathways. Structure. 2009; 17: 1042–1050. 10.1016/j.str.2009.06.008 19679084 PMC2749652

[135] Tsai CJ, Sol AD, Nussinov R. Protein allostery, signal transmission and dynamics: a classification scheme of allosteric mechanisms. Mol Biosyst. 2009; 5: 207–216. 10.1039/b819720b 19225609 PMC2898650

[136] Csermely P, Palotai R, Nussinov R. Induced fit, conformational selection and independent dynamic segments: an extended view of binding events. Trends Biochem Sci. 2010; 35:539–546. 10.1016/j.tibs.2010.04.009 20541943 PMC3018770

[137] Kitano H. A robustness-based approach to systems-oriented drug design. Nat Rev Drug Discov. 2007;6: 202–210. 17318209 10.1038/nrd2195

[138] Beygelzimer A, Grinstein G, Linsker R, Rish I. Improving network robustness by edge modification. Physica A: Statistical Mechanics and its Applications. 2005;357: 593–612.

[139] Louzada VHP, Daolio F, Herrmann HJ, Tomassini M. Smart rewiring for network robustness. Journal of Complex Networks. 2013;1: 150–159.

[140] Yang Y, Li Z, Chen Y, Zhang X, Wang S. Improving the robustness of complex networks with preserving community structure. PLoS One. 2015;10: e0116551 10.1371/journal.pone.0116551 25674786 PMC4326464

[141] Schneider CM, Moreira AA, Andrade JS Jr, Havlin S, Herrmann HJ. Mitigation of malicious attacks on networks. Proc Natl Acad Sci U S A. 2011;108: 3838–3841. 10.1073/pnas.1009440108 21368159 PMC3053993

[142] Csermely P. Structure and dynamics of core/periphery networks. Journal of Complex Networks. 2013;1: 93–123.

[143] del Sol A, Balling R, Hood L, Galas D. Diseases as network perturbations. Curr Opin Biotechnol. 2010;21:566–5671. 10.1016/j.copbio.2010.07.010 20709523

[144] Pei J, Yin N, Ma X, Lai L. Systems biology brings new dimensions for structure-based drug design. J Am Chem Soc. 2014;136:11556–11565. 10.1021/ja504810z 25061983

[145] Csermely P, Korcsmáros T, Kiss HJ, London G, Nussinov R. Structure and dynamics of molecular networks: a novel paradigm of drug discovery: a comprehensive review. Pharmacol Ther. 2013;138:333–408. 10.1016/j.pharmthera.2013.01.016 23384594 PMC3647006

[146] Szilágyi A, Nussinov R, Csermely P. Allo-network drugs: extension of the allosteric drug concept to protein- protein interaction and signaling networks. Curr Top Med Chem. 2013; 13:64–77. 23409766 10.2174/1568026611313010007

[147] Knight ZA, Lin H, Shokat KM. Targeting the cancer kinome through polypharmacology. Nat Rev Cancer. 2010; 10: 130–137. 10.1038/nrc2787 20094047 PMC2880454

[148] Boran ADW, Iyengar R. Systems approaches to polypharmacology and drug discovery. Curr Opin Drug Discov Devel. 2010; 13:297–309. 20443163 PMC3068535

[149] Xie L, Xie L, Kinnings SL, Bourne PE. Novel computational approaches to polypharmacology as a means to define responses to individual drugs. Annu Rev Pharmacol Toxicol. 2012;52: 361–379. 10.1146/annurev-pharmtox-010611-134630 22017683

[150] Berman HM, Westbrook J, Feng Z, Gilliland G, Bhat TN, Weissig H, et al The Protein Data Bank. Nucleic Acids Res. 2000; 28:235–242. 10592235 10.1093/nar/28.1.235 PMC102472

[151] Marti-Renom MA, Stuart A, Fiser A, Sánchez R, Melo A, Sali A. Comparative protein structure modeling of genes and genomes. Annu Rev Biophys Biomol Struct. 2000; 29: 291–325. 10940251 10.1146/annurev.biophys.29.1.291

[152] Fiser A, Do RK, Sali A. Modeling of loops in protein structures. Protein Sci. 2000;9: 1753–1773. 11045621 10.1110/ps.9.9.1753 PMC2144714

[153] Fernandez-Fuentes N, Zhai J, Fiser A. ArchPRED: a template based loop structure prediction server. Nucleic Acids Res. 2006;34:W173–W176. 16844985 10.1093/nar/gkl113 PMC1538831

[154] Phillips JC, Braun R, Wang W, Gumbart J, Tajkhorshid E, Villa E, et al Scalable molecular dynamics with NAMD. J Comput Chem. 2005; 26: 1781–1802. 16222654 10.1002/jcc.20289 PMC2486339

[155] MacKerell AD Jr, Bashford D, Bellott M, Dunbrack RL Jr, Evanseck JD, Field MJ, et al All-atom empirical potential for molecular modeling and dynamics studies of proteins. J Phys Chem B. 1998; 102:3586–3616. 10.1021/jp973084f 24889800

[156] MacKerell AD Jr, Banavali N, Foloppe N. Development and current status of the CHARMM force field for nucleic acids. Biopolymers. 2001;56: 257–265. 10.1002/1097-0282(2000)56:4<257::AID-BIP10029>3.0.CO;2-W 11754339

[157] James KA, Verkhivker GM. Structure-based network analysis of activation mechanisms in the ErbB family of receptor tyrosine kinases: the regulatory spine residues are global mediators of structural stability and allosteric interactions. PLoS One. 2014;9: e113488 10.1371/journal.pone.0113488 25427151 PMC4245119

[158] Andersen HC. Molecular dynamics simulations at constant pressure and/or temperature. J Chem Phys. 1980; 72: 2384–2393.

[159] Nose S, Klein ML. Constant pressure molecular dynamics for molecular systems. Mol Phys. 1983; 50: 1055–1076.

[160] Hoover WG. Canonical dynamics: Equilibration phase-space distributions. Phys Rev A. 1985;31: 1695–1697. 9895674 10.1103/physreva.31.1695

[161] Essmann U, Perera L, Berkowitz ML, Darden T, Lee H, Pedersen LG. A smooth particle mesh Ewald method. J Chem Phys. 1995;103: 8577–8593.

[162] Allen MP, Tildesley DJ. Computer simulation of liquids Oxford: Oxford University Press; 1987.

[163] Ryckaert JP, Ciccotti G, Berendsen HJC. Numerical integration of the cartesian equations of motion of a system with constraints: Molecular dynamics of n-Alkanes. J Comput Phys. 1977;23: 327–341.

[164] Frisch MJ, Trucks GW, Schlegel HB, Scuseria GE, Robb M A, Cheeseman JR, et al Gaussian 03, Revision C.02, Gaussian, Inc.: Wallingford CT; 2004.

[165] Bayly CI, Cieplak P, Cornell W, Kollman PA. A well-behaved electrostatic potential based method using charge restraints for deriving atomic charges: The RESP model. J Phys Chem. 1993; 97:10269–10280.

[166] Humphrey W, Dalke A, Schulten K. VMD: visual molecular dynamics. J Mol Graph. 1996; 14:33–38. 8744570 10.1016/0263-7855(96)00018-5

[167] Mayne CG, Saam J, Schulten K, Tajkhorshid E, Gumbart JC. Rapid parameterization of small molecules using the Force Field Toolkit. J Comput Chem. 2013; 34:2757–2770. 10.1002/jcc.23422 24000174 PMC3874408

[168] Jorgensen WL, Chandrasekhar J, Madura JD, Impey RW, Klein ML. Comparison of simple potential functions for simulating liquid water. J Chem Phys. 1983; 79:926–935.

[169] Vanommeslaeghe K, Hatcher E, Acharya C, Kundu S, Zhong S, Shim J, et al CHARMM general force field: A force field for drug-like molecules compatible with the CHARMM all-atom additive biological force fields. J Comput Chem. 2010; 31:671–690. 10.1002/jcc.21367 19575467 PMC2888302

[170] Vanommeslaeghe K, MacKerell AD Jr. Automation of the CHARMM General Force Field (CGenFF) I: bond perception and atom typing. J Chem Inf Model. 2012; 52:3144–3154. 10.1021/ci300363c 23146088 PMC3528824

[171] Vanommeslaeghe K, Raman EP, MacKerell AD Jr. Automation of the CHARMM General Force Field (CGenFF) II: assignment of bonded parameters and partial atomic charges. J Chem Inf Model. 2012; 52:3155–3168. 10.1021/ci3003649 23145473 PMC3528813

[172] Yesselman JD, Price DJ, Knight JL, Brooks CL 3rd. MATCH: an atom-typing toolset for molecular mechanics force fields. J Comput Chem. 2012; 33:189–202. 10.1002/jcc.21963 22042689 PMC3228871

[173] Koukos PI, Glykos NM. Grcarma: A fully automated task-oriented interface for the analysis of molecular dynamics trajectories. J Comput Chem. 2013;34:2310–2312. 24159629 10.1002/jcc.23381

[174] Eyal E, Yang LW, Bahar I. Anisotropic network model: systematic evaluation and a new web interface, Bioinformatics. 2006;22:2619–2627. 16928735 10.1093/bioinformatics/btl448

[175] Moreira IS, Fernandes PA, Ramos MJ. Computational alanine scanning mutagenesis—an improved methodological approach. J Comput Chem. 2007;28: 644–654. 17195156 10.1002/jcc.20566

[176] Kannan N, Vishveshwara S. Identification of side-chain clusters in protein structures by a graph spectral method. J Mol Biol. 1999; 292:441–464. 10493887 10.1006/jmbi.1999.3058

[177] Brinda KV, Vishveshwara S. A network representation of protein structures: implications for protein stability. Biophys J. 2005; 89: 4159–4170. 16150969 10.1529/biophysj.105.064485 PMC1366981

[178] Sethi A, Eargle J, Black AA, Luthey-Schulten Z. Dynamical networks in tRNA:protein complexes. Proc Natl Acad Sci USA. 2009;106: 6620–6625. 10.1073/pnas.0810961106 19351898 PMC2672494

[179] Floyd RW. Algorithm 97: Shortest Path. Commun ACM. 1962;5: 345.

[180] Punta M, Coggill PC, Eberhardt RY, Mistry J, Tate J, Boursnell C, et al The Pfam protein families database. Nucleic Acids Res. 2012; 40:D290–D301. 10.1093/nar/gkr1065 22127870 PMC3245129

[181] Finn RD, Bateman A, Clements J, Coggill P, Eberhardt RY, Eddy SR, et al Pfam: the protein families database. Nucleic Acids Res. 2014; 42 D222–D230. 10.1093/nar/gkt1223 24288371 PMC3965110

[182] Finn RD, Miller BL, Clements J, Bateman A. iPfam: a database of protein family and domain interactions found in the Protein Data Bank. Nucleic Acids Res. 2014;42:D364–D373. 10.1093/nar/gkt1210 24297255 PMC3965099

[183] Wu CH, Apweiler R, Bairoch A, Natale DA, Barker WC, Boeckmann B, et al The Universal Protein Resource (UniProt): an expanding universe of protein information. Nucleic Acids Res. 2006;34: D187–D191. 16381842 10.1093/nar/gkj161 PMC1347523

[184] Consortium UniProt. UniProt: a hub for protein information. Nucleic Acids Res. 2015;43:D204–D212. 10.1093/nar/gku989 25348405 PMC4384041

