# Supplementary figures and images for: Molecular Determinants Underlying Binding Specificities of the ABL Kinase Inhibitors: Combining Alanine Scanning of Binding Hot Spots with Network Analysis of Residue Interactions and Coevolution
Source: PLoS One. 2015 Jun 15;10(6):e0130203. doi: 10.1371/journal.pone.0130203 (PMC4468085; doi:10.1371/journal.pone.0130203)

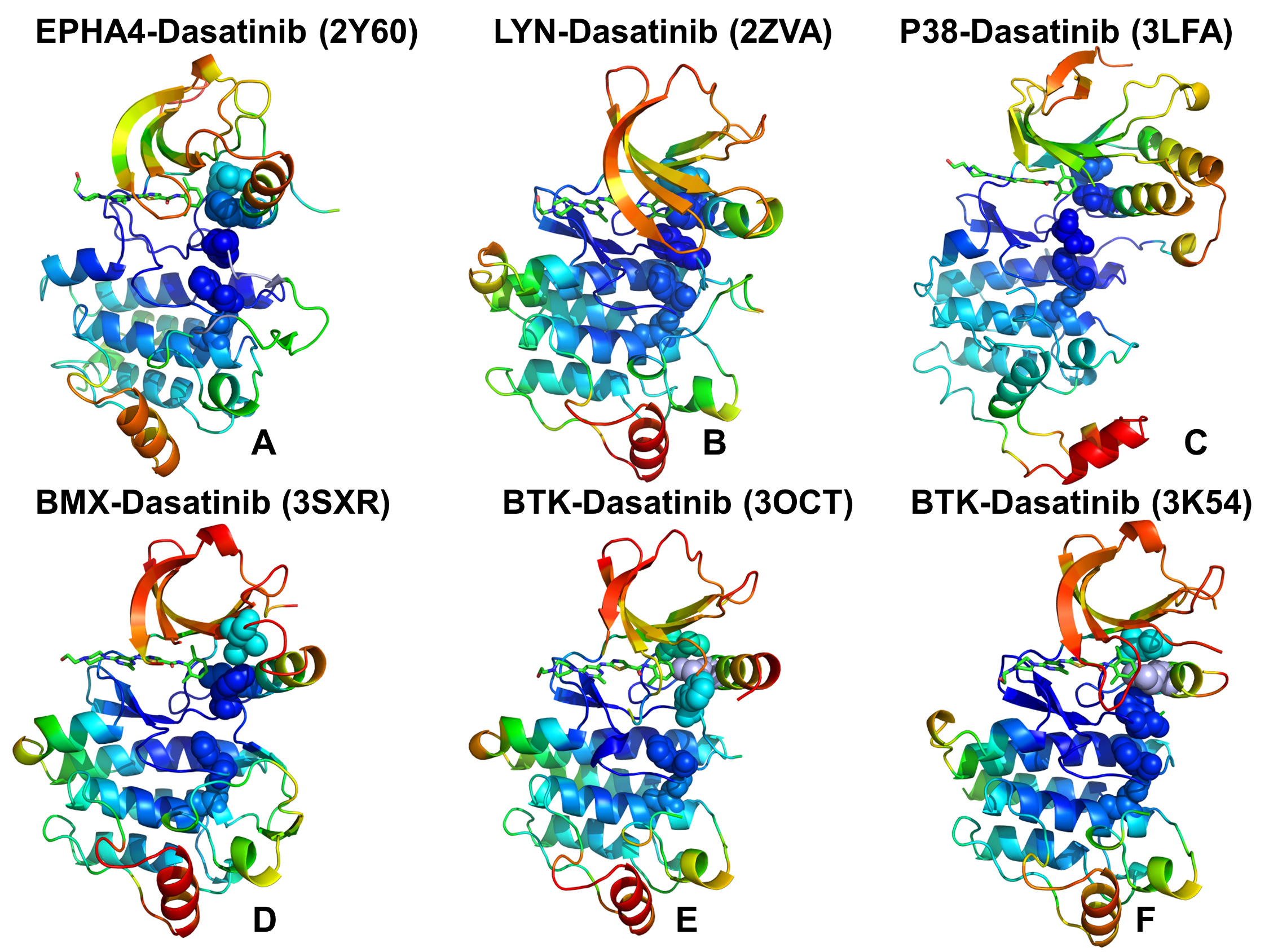

Supplement: S1 Fig — Conformational dynamics profiles are shown for the crystal structures of Dasatinib complexes with EPHA4 (A); LYN (B), P38 (C), BMX (D), BTK (E,F). An active DFG-in conformation is adopted in Dasatinib complexes with EPHA4 (pdb id 2Y6O), LYN (pdb id 2ZVA), P38 (pdb id 3LFA), and BTK kinases (pdb id 3K54). Dasatinib binds with the inactive structures of BMX (pdb id 3SXR) and BTK kinases (pdb id 3OCT). In the complex with an inactive BTK conformation (pdb id 3OCT) an intermediate DFG position is adopted which is between the fully DFG-in and DFG-out conformations. Conformational dynamics profiles were computed by projecting MD trajectories onto the essential space of the three lowest frequency modes. The color gradient from blue to red indicates the decreasing structural rigidity (or increasing conformational mobility) of the protein residues and refers to an average value over the backbone atoms in each residue. The R-spine residues are annotated in spheres and colored according to their degree of structural stability. Dasatinib binding modes are shown in sticks and atom-based color-coded. The Pymol program was used for visualization of the protein kinase structures (The PyMOL Molecular Graphics System, Version 1.2r3pre, Schrödinger, and LLC). (TIF) [file pone.0130203.s001.tif]

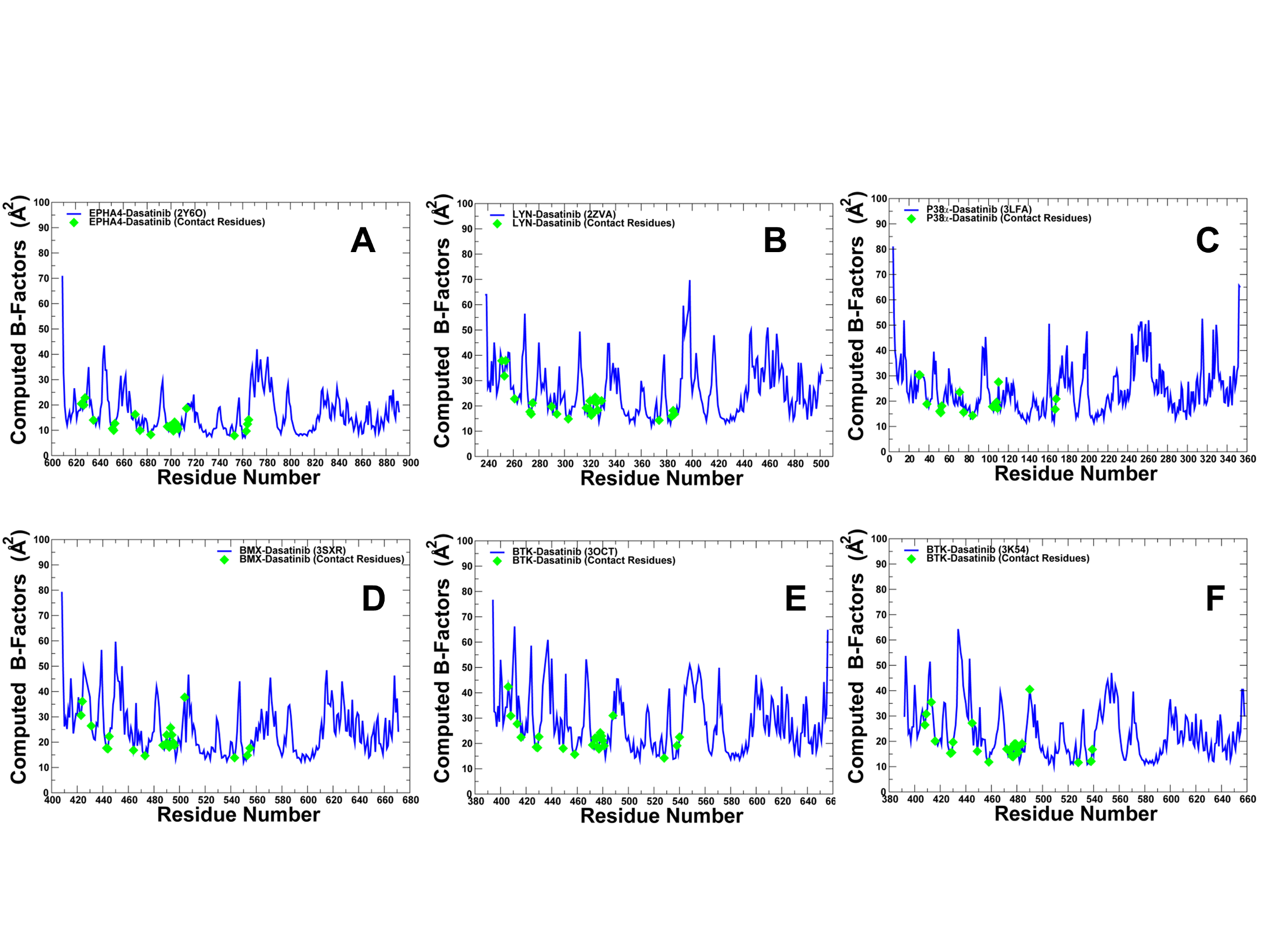

Supplement: S2 Fig — The computed B-factors obtained from MD simulations of Dasatinib complexes with EPHA4 (A); LYN (B), P38 (C), BMX (D), BTK (E,F). The fluctuations of the inhibitor-interacting residues are highlighted (green diamonds) indicating stability of the binding site residues in simulations. (TIF) [file pone.0130203.s002.tif]

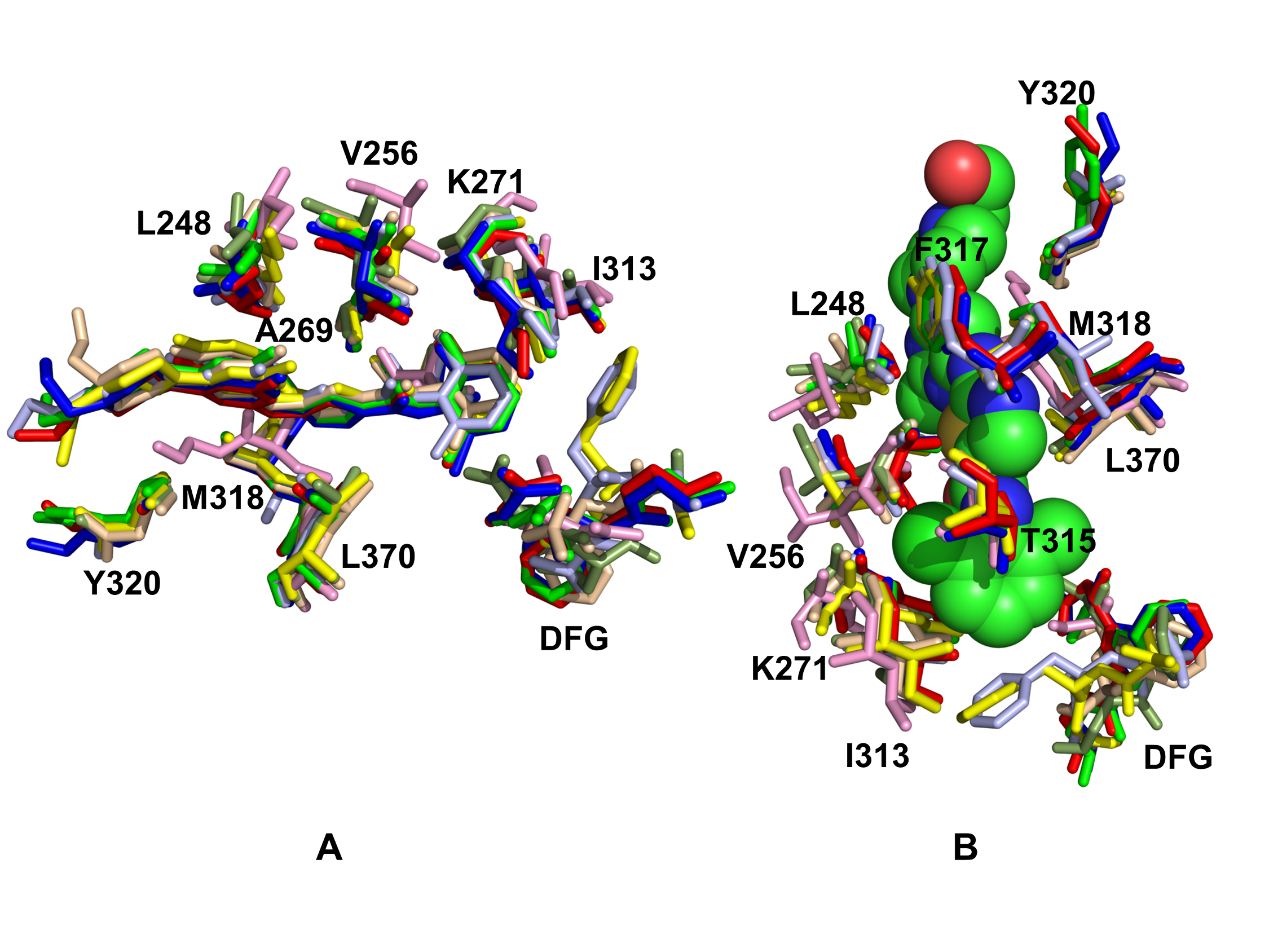

Supplement: S3 Fig — Structural alignment of the ensemble-average positions for the binding site residues are shown for Dasatinib complexes with ABL, SRC, EPHA4, LYN, P38, BMX, and BTK kinases. The annotated residues correspond to the ABL residue numbering in the Dasatinib-ABL complex (pdb id 2GQG). The front view (A) and top view (B) show structural stability of these residues (most notably T315, F317, M318, and L370) across all complexes and indicate some positional variability of the DFG motif. (TIF) [file pone.0130203.s003.tif]

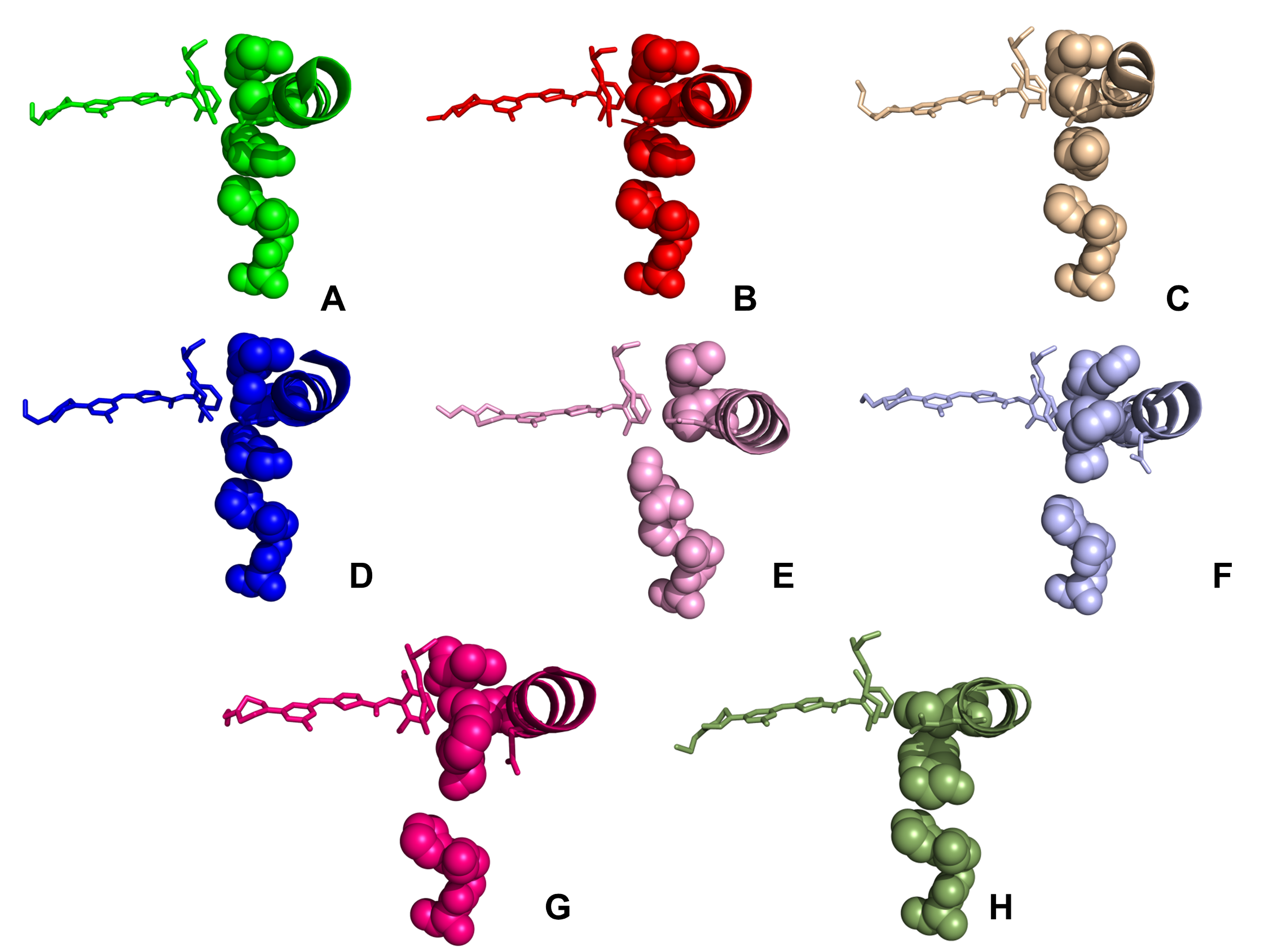

Supplement: S4 Fig — The ensemble-average binding mode of Dasatinib and functional regions (αC-helix, R-spine, K-E catalytic pair) from MD simulations of Dasatinib complexes with EPHA4 (A); LYN (B), P38 (C), BMX (D), BTK (E,F). In Dasatinib complexes with the DFG-out inactive conformations of BMX (D) and BTK (E), the R-spine is partially disassembled, the αC-helix moved away from the catalytically competent position, and the characteristic salt bridge K-E is broken. The binding mode of Dasatinib remained stable in MD simulations of the crystal structures in all Dasatinib-kinase complexes. The R-spine residues are annotated in spheres, the αC-helix is shown in ribbons, and the binding mode of Dasatinib is shown in sticks. The residues forming a catalytic K-E salt bridge in the active site are shown in sticks. (TIF) [file pone.0130203.s004.tif]

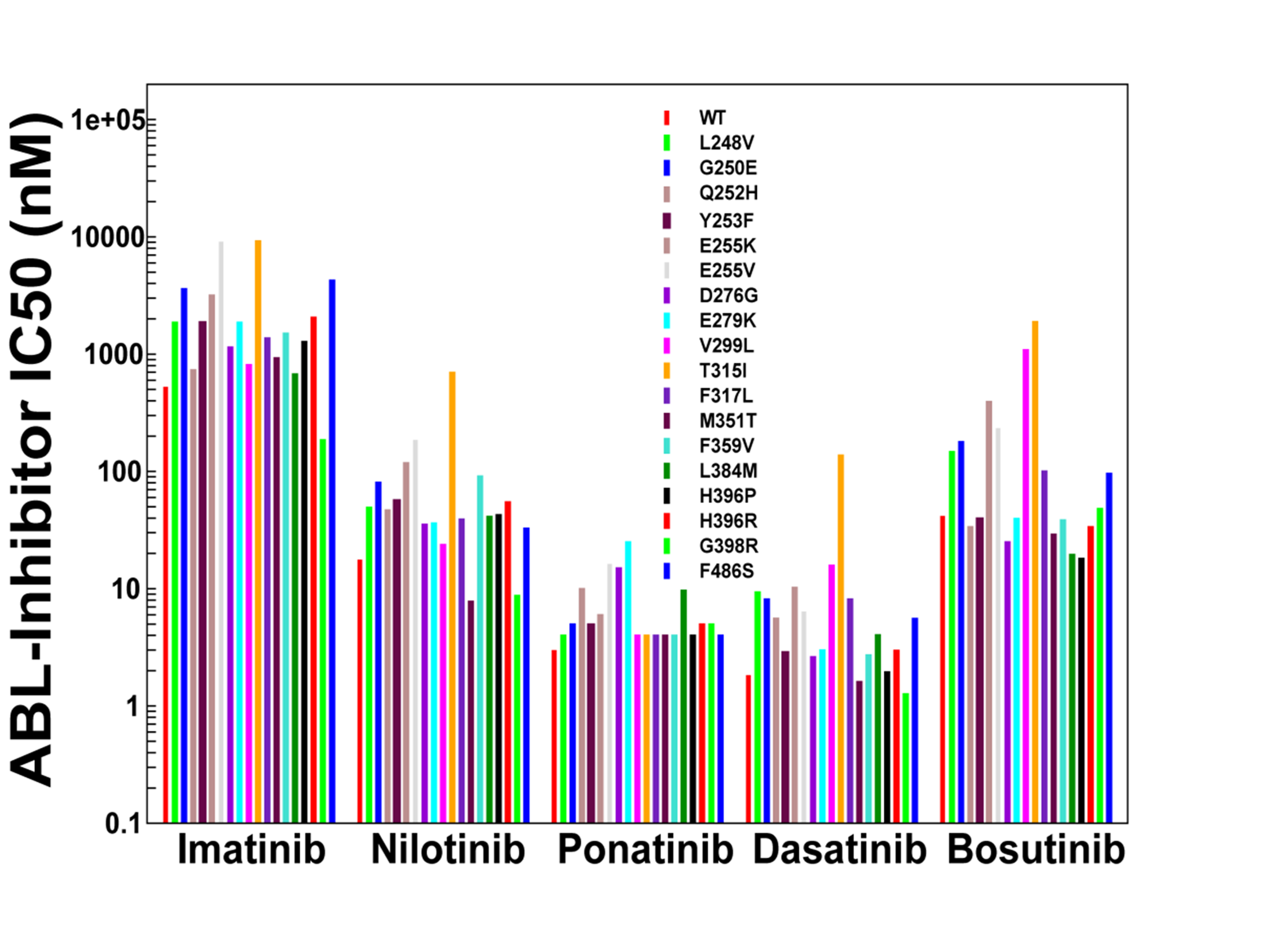

Supplement: S5 Fig — The experimental IC50 values of kinase inhibitors (Imatinib, Nilotinib, Ponatinib, Dasatinib, Bosutinib) against ABL kinase are collected from biochemical and clinical studies [67–69]. These experimental studies confirmed a range of mutations (shown in different colors) where changes in the inhibition constants correlate with the degree of drug resistance. The logarithmic scale for IC50 values is employed. (TIF) [file pone.0130203.s005.tif]

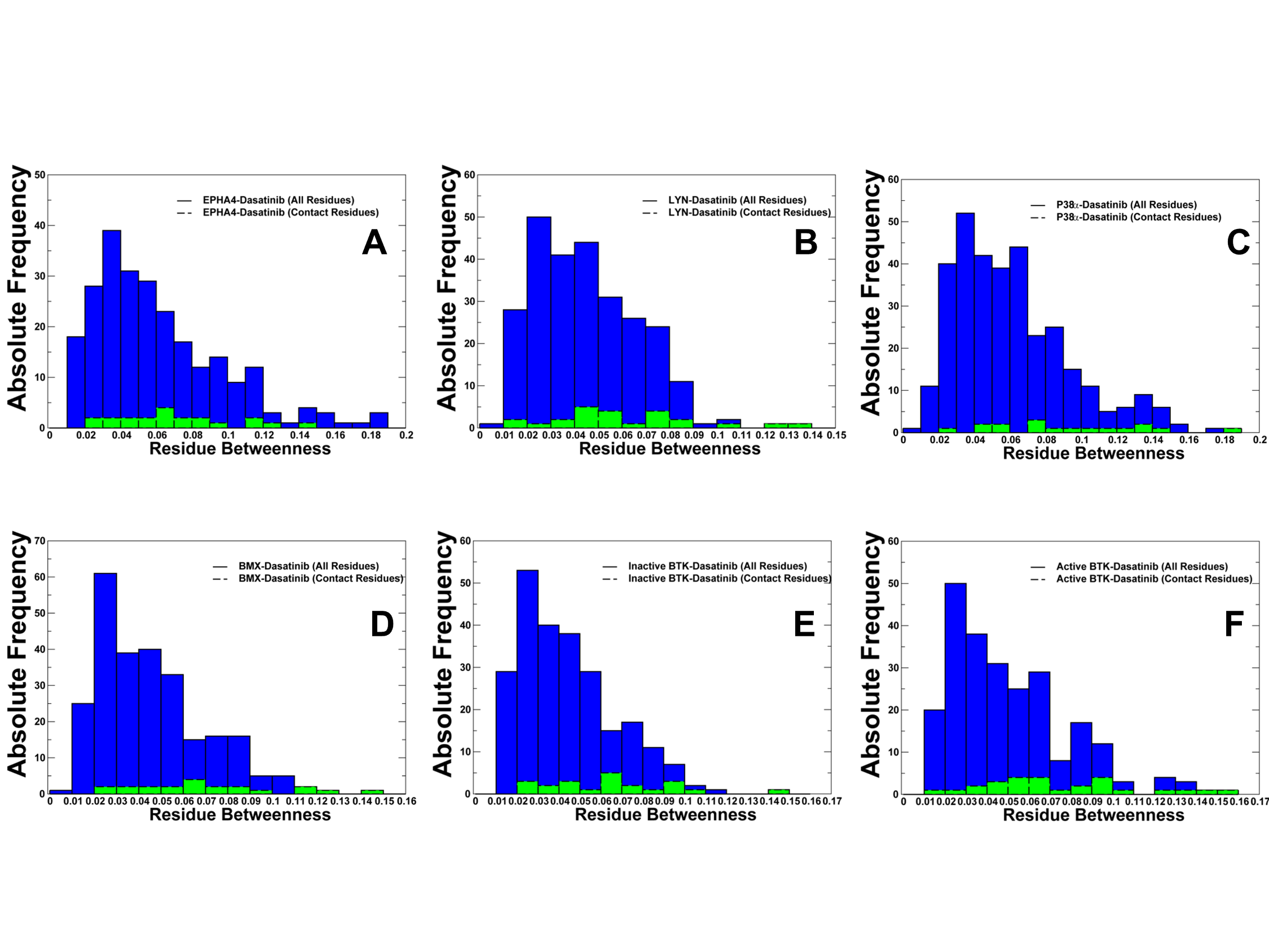

Supplement: S6 Fig — The residue-based betweenness distributions of Dasatinib complexes with EPHA4 (A), LYN (B), P38 (C), BMX (D), BTK (E,F). The distributions are obtained by averaging the results of MD simulations. The overall distributions are shown in blue and the distribution corresponding to the inhibitor-interacting residues is shown in green. (TIF) [file pone.0130203.s006.tif]
